# Palaeontological evidence reveals convergent evolution of intervertebral joint types in amniotes

**DOI:** 10.1038/s41598-020-70751-2

**Published:** 2020-08-24

**Authors:** Tanja Wintrich, Martin Scaal, Christine Böhmer, Rico Schellhorn, Ilja Kogan, Aaron van der Reest, P. Martin Sander

**Affiliations:** 1 Section Paleontology, Institute of Geosciences, University of Bonn, Nussallee 8, 53115 Bonn, Germany; 2 Institute of Anatomy, University of Bonn, Nussallee 10, 53115 Bonn, Germany; 3 Institute of Anatomy II, University of Cologne, Joseph-Stelzmann-Str. 9, 50937 Cologne, Germany; 4 UMR 7179 CNRS, Département Adaptations du Vivant, Muséum National D’Histoire Naturelle, case postale 55, 57 rue Cuvier, 75231 Paris Cedex 05, France; 5 Department of Palaeontology and Stratigraphy, Geological Institute, TU Bergakademie Freiberg, Bernhard-von-Cotta-Str. 2, 09596 Freiberg, Germany; 6 Institute of Geology and Petroleum Technologies, Kazan Federal University, Kremlyovskaya Str. 4/5, 420008 Kazan, Russia; 7 Department of Biological Sciences, University of Alberta, Edmonton, AB T6G 2E9 Canada; 8 Dinosaur Institute, Natural History Museum Los Angeles County, 900 Exposition Boulevard, Los Angeles, CA 90007 USA

**Keywords:** Anatomy, Developmental biology, Cartilage development, Morphogenesis, Evolution, Coevolution, Evolutionary developmental biology, Evolutionary theory, Palaeontology

## Abstract

The intervertebral disc (IVD) has long been considered unique to mammals. Palaeohistological sampling of 17 mostly extinct clades across the amniote tree revealed preservation of different intervertebral soft tissue types (cartilage, probable notochord) seen in extant reptiles. The distribution of the fossilised tissues allowed us to infer the soft part anatomy of the joint. Surprisingly, we also found evidence for an IVD in fossil reptiles, including non-avian dinosaurs, ichthyosaurs, plesiosaurs, and marine crocodiles. Based on the fossil dataset, we traced the evolution of the amniote intervertebral joint through ancestral character state reconstruction. The IVD evolved at least twice, in mammals and in extinct diapsid reptiles. From this reptilian IVD, extant reptile groups and some non-avian dinosaurs independently evolved a synovial ball-and-socket joint. The unique birds dorsal intervertebral joint evolved from this dinosaur joint. The tuatara and some geckos reverted to the ancestral persisting notochord.

## Introduction

Morphology of the vertebral column has provided zoologists with key anatomical characters for groups of amniotes^1^. This includes the intervertebral disc (IVD), a soft tissue feature, which has been considered to be unique to mammals^2–4^. However, although the importance of soft tissue analysis in fossils is clearly recognised today^5–7^, investigations on soft tissue in the vertebral column, namely of joints and intervertebral spaces, have not been done^5^. Neither has the evolution of the intervertebral joint been reconstructed using phylogenetic methods such as ancestral state reconstruction (ASR)^8^.

The intervertebral disc (IVD) is a fibrocartilaginous synarthrotic joint connecting the vertebral centra of mammals. It provides intervertebral flexibility, allowing for a wide range of movements and is involved in shock absorbance and transmission of mechanical forces^9,10^. The IVD is thus of eminent biomechanical and clinical importance in the human vertebral column and has been studied from multiple perspectives in biological, medical and veterinary science^11–13^. The crucial function of the IVD is to withstand forces acting on the axial skeleton, and gradual failure of IVD function from degenerative processes often leads to pathological symptoms^14^.

The mammalian IVD is composed of two distinct parts, the nucleus pulposus (NP) and the annulus fibrosus (AF). The NP is the hydrophilic proteoglycan-rich gelatinous core of the IVD. Surrounding the NP is the AF, a lamellate ring of spirally arranged layers of collagen I and fibrocartilage. The joint formed by the IVD is further stabilized by intervertebral ligaments that insert in the periosteal cortex of the adjacent vertebral centra.

Due to the lack of soft tissue preservation in fossils, however, the evolutionary origins of the IVD among Amniota (true land animals) have been poorly understood^10^. As increasing numbers of fossil amniote specimens with preserved soft tissues are discovered, analyses of these fossils have the potential of modifying our view of the evolution of the IVD. Since a joint is an integrated system of bone and other connective tissues, the bone histology of the amniote centrum is important as well. Thus, the AF is anchored to the peripheral part of the two centra via fibrocartilage and direct insertion of collagen fibres into the bone^15^. The NP, on the other hand, has no direct contact with the bony surface of the centrum, being separated by a layer of hyaline cartilage (cartilaginous endplate, see SI for details). The NP is developmentally derived from notochordal cells^9–11^.

In comparative anatomy, the notion prevails that reptiles lack an IVD because of their procoelous and opisthocoelous vertebral centrum morphology^1,3,4,16^, forming a synovial ball-and-socket joint. Such a joint has a thin layer of hyaline cartilage covering the bony articular surface, with a synovial fluid-filled space separating the two articular surfaces that are peripherally connected by the joint capsule of connective tissue. Among extant reptiles, the only exceptions to this type of joint are the tuatara *Sphenodon punctatus*^17,18^ and some gekkonid lizards^19^. In the fossil record, however, we see that generalised centrum morphology does not indicate ball-and-socket joints^1^ with hyaline cartilage. This agrees with higher-level amniote phylogeny that reveals the basal condition of vertebral morphology to be an amphicoelous vertebral centrum with a notochordal canal^1,10^, as already seen in the amniote sistergroup, Diadectomorpha^1^ (Fig. 2). The colonisation of diverse terrestrial environments, including the conquest of the arboreal and aerial habitats, were accompanied by substantial changes in the morphology of the vertebrae, but also in the articulations between these segments where motion occurs^1^. Romer^1^ described a general evolutionary progression from amphicoelous notochordal via amphicoelous non-notochordal to platycoelous during the transition from aquatic to terrestrial environments and hypothesized that the ‘conjoined hollows created by amphicoelous vertebral centra were filled by modified notochordal material or fibrous tissue’, without raising the issue of the IVD and its evolution.

Except for the well-defined IVD and synovial joints, inconsistencies in terminology describing inferred intervertebral tissues (e.g., intervertebral cartilage, intervertebral tissue, and intervertebral pad) have caused some confusion when referring to tissues and joint anatomy of fossils. Furthermore, if we turn to developmental biology, mammals and birds (or archosauromorphs) are in focus of the research, not reptiles. However, regardless of vertebral centrum shape of extinct Amniota, there must have been connective soft tissues which allowed three-dimensional movement of the vertebral column. In the palaeontological literature, there are few mentions about IVDs in fossils at all such as the studies by Witzmann et al.^20^ and Hopley^21^. The latter describes a spinal pathology in a plesiosaur vertebra suggesting a disc prolapse and consisting of a Schmorl´s node, which is a pathologic intrusion of the NP into the bony endplate. However, Hopley^21^ does not discuss the presence of the Schmorl´s node as evidence for IVDs in plesiosaurs but simply assumes their presence. Rothschild & Berman^22^ describe the preservation of the intervertebral space in the caudal vertebrae of a sauropod dinosaur. Finally, Pérez García and Gascó^23^ interpret a disk-shaped fossil from the Jurassic of Spain as an ossified IVD of a marine reptile. However, the very brief description is insufficient for evaluating this interpretation.

Here, we identify the different tissue types present in the intervertebral space of fossil amniotes based on palaeohistological investigations of bone and preserved soft tissues (Table 1). Based on the rationale that soft tissues associated with the skeleton might also be preservable, especially in articulated specimens, we histologically sampled diverse fossil taxa across the amniote tree for preserved intervertebral soft tissues (Table S1). If possible, we sampled one or several dorsal vertebrae in articulation. We restricted the analysis to the dorsal vertebral column because it shows the least morphological and functional variation (turtles being the exception) compared to the neck and the tail columns, thus improving comparability across taxa.

**Table 1:** Summary of results and description of character states for ancestral state reconstruction.

Char. State	Centrum shape	Fossil articular surface morphology and fossil hard and soft tissues	Anatomical structures and tissues in living animal	Joint type	Occurrence in this study
0	Amphicoelous, notochordal	I. Notochord foramen (Figs. 3a, S3, S4, S7)	I. Persisting notochord, expanded in intervertebral space (Fig. 1a)	Plesiomorphic amniote	Stem amniotes, basal synapsids, basal reptiles, *Sphenodon*
		II. Central concave area covered by thin chondrocyte layer or bone (Figs. 3a, S2, S4d)	II. Area of expanded notochord, possibly incipient *nucleus pulposus*, separated by cartilage from bone (Fig. 1e)		
		III. Irregularly shaped and loosely packed bodies the size of large chondrocytes or translucent, coarsely crystalline matter in the intervertebral space (Figs. 3a, S7)	III. Possible notochordal or incipient *nucleus pulposus* cells (Fig. 1d)		
		IV. Peripheral convex area with chondrocyte files (serial cartilage) and intervening bone spicules (Figs. S1b–d, S3b–d, S4b–d, S7c)	IV. Fibrocartilage of *annulus fibrosus* inserting into serial cartilage and bony spicules (Fig. 1b)		
		IV. Sharpey’s fibres peripheral to articular surface in periosteal bone (Fig. S4b)	IV. Joint capsule (Fig. 1c)		
1	Amphicoelous, non-notochordal	I. Notochord foramen absent (Figs. S8a, S10a, S11a)	I. Notochord reduced or absent	Intervertebral disc	hupehsuchians, ichthyosaurs, placodonts
		II. Central concave area of articular surface with thin layer of irregular chondrocytes (Figs. 3d, S10d, S12c)	II. Globular *nucleus pulposus* separated by cartilage from bone (Fig. 1e)		
		III. Irregularly shaped and loosely packed bodies or grains larger than chondrocytes in the intervertebral space (Figs. 3c, S9b, S11, S12)	III. Possible *nucleus pulposus* cells or notochord cells (Fig. 1d)		
		IV. Peripheral convex area of articular surface with chondrocyte files (serial cartilage) and intervening bone spicules (Figs. 3e, S8b, S9b, c, S10b, S12a, b)	IV. Fibrocartilage of *annulus fibrosus* inserting into serial cartilage and bony spicules in articular surface periphery (Fig. 1b)		
		V. Sharpey’s fibres peripheral to articular surface in periosteal bone	V. Joint capsule		
2	Platycoelous	I. Notochord foramen absent (Figs. S19a, S20a, S22a, S23a)	I. Notochord absent (Fig. S5a)	Intervertebral disc	Mammals, eosauropterygians, most archosauromorphs, including most dinosaurs
		II. Flat central area of articular surface with irregular chondrocyte cover, usually thin (Figs. S19, S20c, S22c, S24a)	II. Lens-shaped *nucleus pulposus* separated by cartilage from underlying bone (Figs. 2a, b, S5b)		
		III. Irregularly shaped and loosely packed bodies the size of large chondrocytes in the intervertebral space (Fig. S22b)	III. Possible *nucleus pulposus* cells		
		IV. Peripheral area of articular surface with peripherally inclined chondrocyte files (serial cartilage) and bone spicules (Figs. 3b, 5i–l, S6b,c, S20b, S22d, S24b,c, S25b–e)	IV. Fibrocartilage of *annulus fibrosus* inserting into serial cartilage and bony spicules in articular surface periphery (Figs. 2c, 5e–h, S5c)		
		V. Sharpey’s fibres peripheral to articular surface in periosteal bone	V. Joint capsule		
3	Procoelous /opistho-coelous	I. Notochord foramen absent (Figs. S15a, S17a)	I. Notochord absent (Figs. 5a, S16a)	Synovial ball-and-socket	Squamates, eusuchians
		II. Convex/concave joint surface with unorganized chondrocytes or serial cartilage with files normal to joint surface overlying bone, no bony spicules (Figs. 2e,f, 5b–d), S17b,c, S17b,c)	II. Convex/concave joint surface covered by hyaline cartilage bordering on fluid-filled joint space encased by joint capsule (Figs. 2d, 5a–d, S16b,c)		
		III. Sharpey’s fibres peripheral to articular surface in periosteal bone	III. Joint capsule (Figs. 5a, S16b)		
4	Procoelous	I. Notochord foramen absent (Fig. S28a)	I. Notochord absent	Fibrous, bird-type	Dromaeosaurs, birds
		II. Convex/concave surface with irregular, scattered chondrocytes in bone with fibre insertions (Figs. S26b–d, S27b,c, S28b,c)	II. Convex/concave joint surface covered by irregular, thin cartilage, connective tissue fibers inserting into bone of joint surface		
		III. Sharpey’s fibres peripheral to articular surface in periosteal bone	III. Joint capsule		

**Figure 1:**
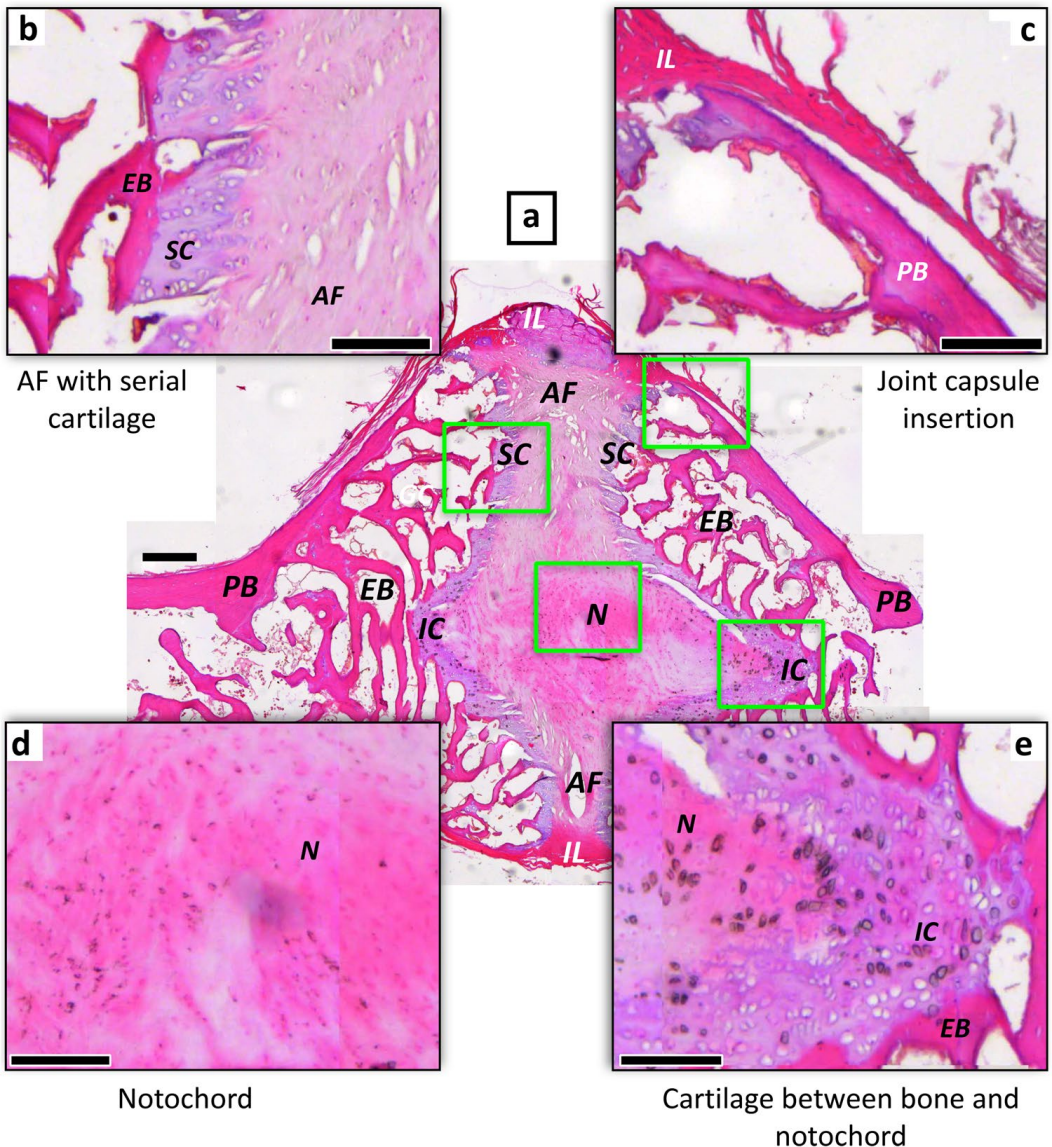
Histology of the plesiomorphic amniote joint with persisting notochord of *Sphenodon punctatus* NHMW 8108. The intervertebral tissues and the two articulating mid-dorsal centra illustrate the relationship between mineralised (fossilisable) tissues and soft tissues. (**a**) Oblique sagittal microtome section stained with hematoxylin. Note that the notochord appears discontinuous in the image because of the suboptimal plane of section. (**b**) Enlargement of annulus fibrosus insertion into endochondral bone of articular surface via serial cartilage. (**c**) Enlargement of interspinal ligament of the joint capsule inserting into periosteal bone of the centrum peripheral surface. (**d**) Enlargement of notochordal tissue in intervertebral space. (**e**) Enlargement of contact between notochordal tissue and endochondral bone of articular surface of centrum with intervening irregular cartilage. Scale bar in (**a**) represents 100 µm, and scale bars in (**b**–**e**) represent 40 µm. *AF* annulus fibrosus, *EB* endochondral bone, *IC* irregular cartilage, *IL* intervertebral ligament, *N* notochordal tissue, *PB* periosteal bone, *SC* serial cartilage.

Relationship between vertebral centrum shape, observed fossilised tissues (bony tissues, altered cartilaginous tissues, and altered soft tissues), inferred anatomical structures, tissue and cell types, inferred joint type, and systematic occurrence in this study. This table is complimentary to Fig. 1. Other pertinent figures are referenced as well. The lack of a figure reference does not mean that the feature is not present, it simply means that it is not illustrated. *Char. state* character state.

For the reader to be able to understand our results without delving too deeply into the “Methods” section, we here offer a brief review of the linkage between the fossil hard and soft tissues and the reconstruction of intervertebral joint anatomy, including the five types of intervertebral joints encountered in this study (Fig. 1, Table 1). In general, any vertebral centrum and any long bone in the amniote skeleton, fossil or extant, ossifies from two domains, the endochondral domain and the periosteal domain. In former, the bone tissue has a cartilage precursor, hence the term “replacement bone”. In the latter, bone tissue is deposited directly by an osteoblast epithelium, the periost.

The anterior and posterior articular faces of any vertebral centrum are largely formed by endochondral bone, whereas the vertebral surface in between is formed by periosteal bone (Fig. 1). Any cartilage pertains to the endochondral domain (hence the name), and any ligaments and tendons inserts into the periosteal domain via Sharpey’s fibres (Fig. 1). Different kinds of vertebral centrum shapes originate from the interplay of the rates and directions of tissue formation in the two domains.

Beginning with the plesiomorphic amniote joint type, still seen in *Sphenodon* today (Fig. 1), the persistence of the notochord in fossil samples is indicated by the presence of a notochord foramen and sometimes by fossilised notochordal tissue. This joint already possessed a joint capsule (Fig. 1; like all other joints with the possible exception of the fibrous bird-type joint). The presence and location of the joint capsule is indicated by Sharpey’s fibres inserting into the periosteal bone around the margin of the articular surface. The plesiomorphic amniote joint also possessed an AF. This is indicated by serial cartilage consisting of files of fossil chondrocytes in the peripheral area of the articular surface, often with interspersed bone spicules with inserting fibres of the fibrocartilage of the AF (Fig. 1b). A thin layer of fossil chondrocytes with an irregular arrangement in the center of the articular surface as well as possible fossil notochordal cells indicate the former presence of an expanded notochord or an incipient NP (Fig. 1e).

The former presence of an IVD is indicated by the lack of a notochordal foramen, the former presence of an AF (see above) and sometimes the preservation of possible NP tissue. The size and shape of the former AF and NP are indicated by the distribution of irregular cartilage vs. serial cartilage on the articular surface as well as by the peripheral inclination of the chondrocyte files in the serial cartilage (Fig. 1). In amphicoelous vertebrae, the NP probably was spherical in shape, and in platycoelous vertebrae, it was lens-shaped, as in humans today. The amphicoelous joint with an IVD is extinct.

**Figure 2:**
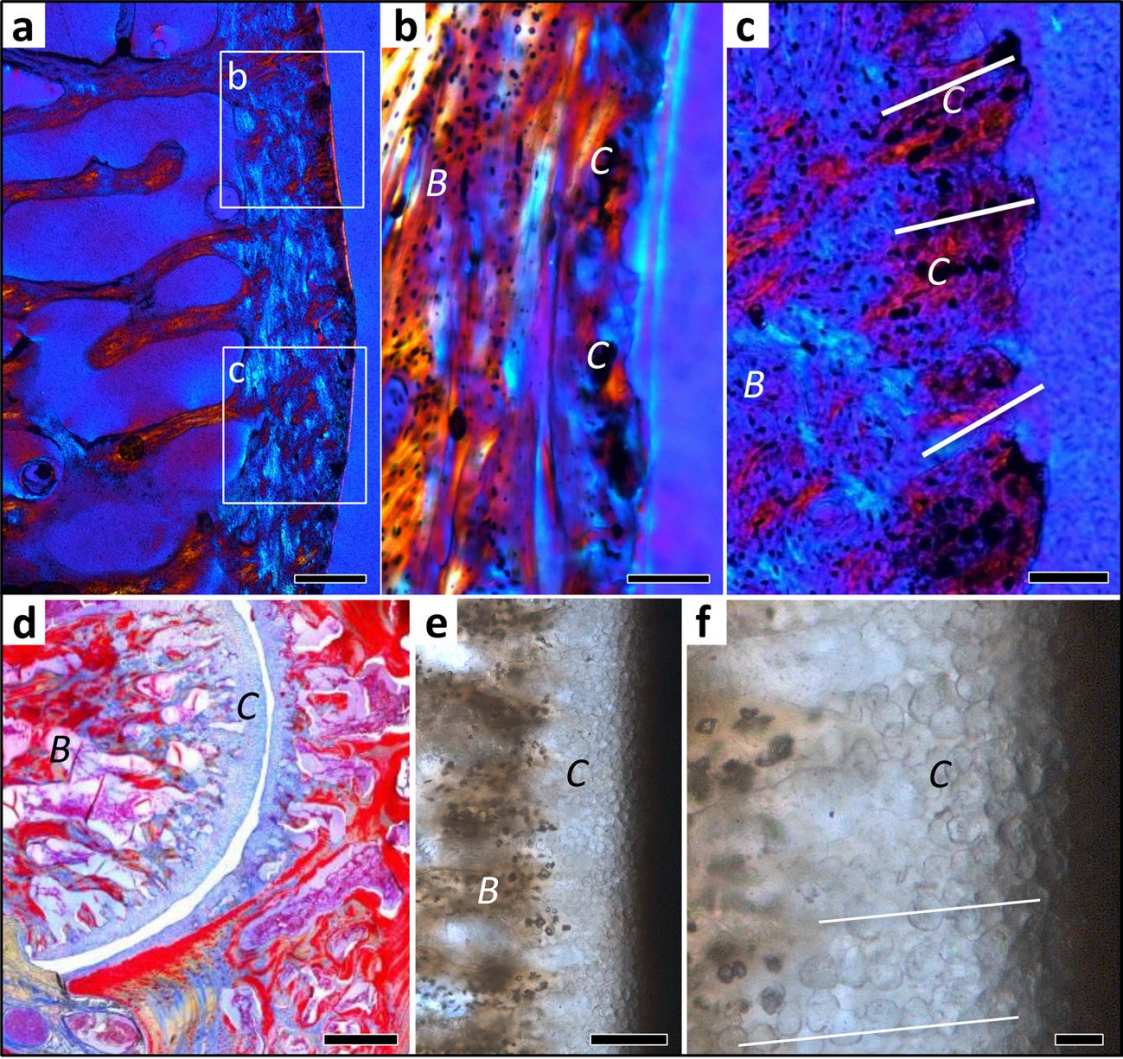
Histology of mammalian and squamate intervertebral spaces. (**a**) Extant *Phoca vitulina* IGPB M 60, sagittal ground section of dorsal vertebral centrum showing part of the bony endplate in cross-polarised light with lambda filter. Note that the colors in this and the following two images do not result from histological staining but from polarised light. (**b**) Enlargement of area b in (**a**) showing a thin layer of cartilage. Note the irregular arrangement of the chondrocyte lacunae. This tissue is overlain by the nucleus pulposus (NP) of the IVD in life. (**c**) Enlargement of area c in (**a**) showing cartilage chondrocytes in inclined files (white lines) embedded in fibrous bony tissue. This represents the insertion of the annulus fibrosus (AF) of the IVD. (**d**) Extant *Python* sp. IGPB R 662, transverse microtome section of the synovial joint connecting the dorsal vertebral centrum. This joint is formed by hyaline cartilage and a thin intervertebral space filled with synovial fluid in life. (**e**) Fossil *Mosasaurus missouriensis* IGPB Goldfuß 1230, close up of the joint surface in sagittal section of a dorsal vertebral centrum. Note the globular structures arranged in files representing fossilised hyaline cartilage. (**f**) Enlargement of (**e**), white lines highlight some files. Scale bars in (**a**, **d**) represent 500 µm, scale bars in (**b**, **c**, **e**) represent 100 µm, and scale bar in (**f**) represents 20 µm. *B* bone tissue, *C* cartilage.

Beyond the tight fit of the articular surfaces in a socket and ball, the former presence of a synovial ball-and-socket joint lined with articular cartilage is indicated by the even layer of fossil serial cartilage on the bony articular surface. The fossil chondrocyte files in this layer are oriented normal to the articular surface and do not diverge peripherally (Fig. 2d−f). In well preserved specimens, the entire layer of articular cartilage and even the joint cavity formerly filled by synovial fluid can be observed (Fig. S15a,b).

Finally, the former presence of a fibrous bird-type joint is indicated by the close fit of the articular surfaces in a socket and ball or in two saddles, but the fossil articular surface is mainly bony with fine fibre insertions and only a thin layer of irregular and widely spaced fossil chondrocytes (Figs. S26–S28).

## Results

**Figure 3:**
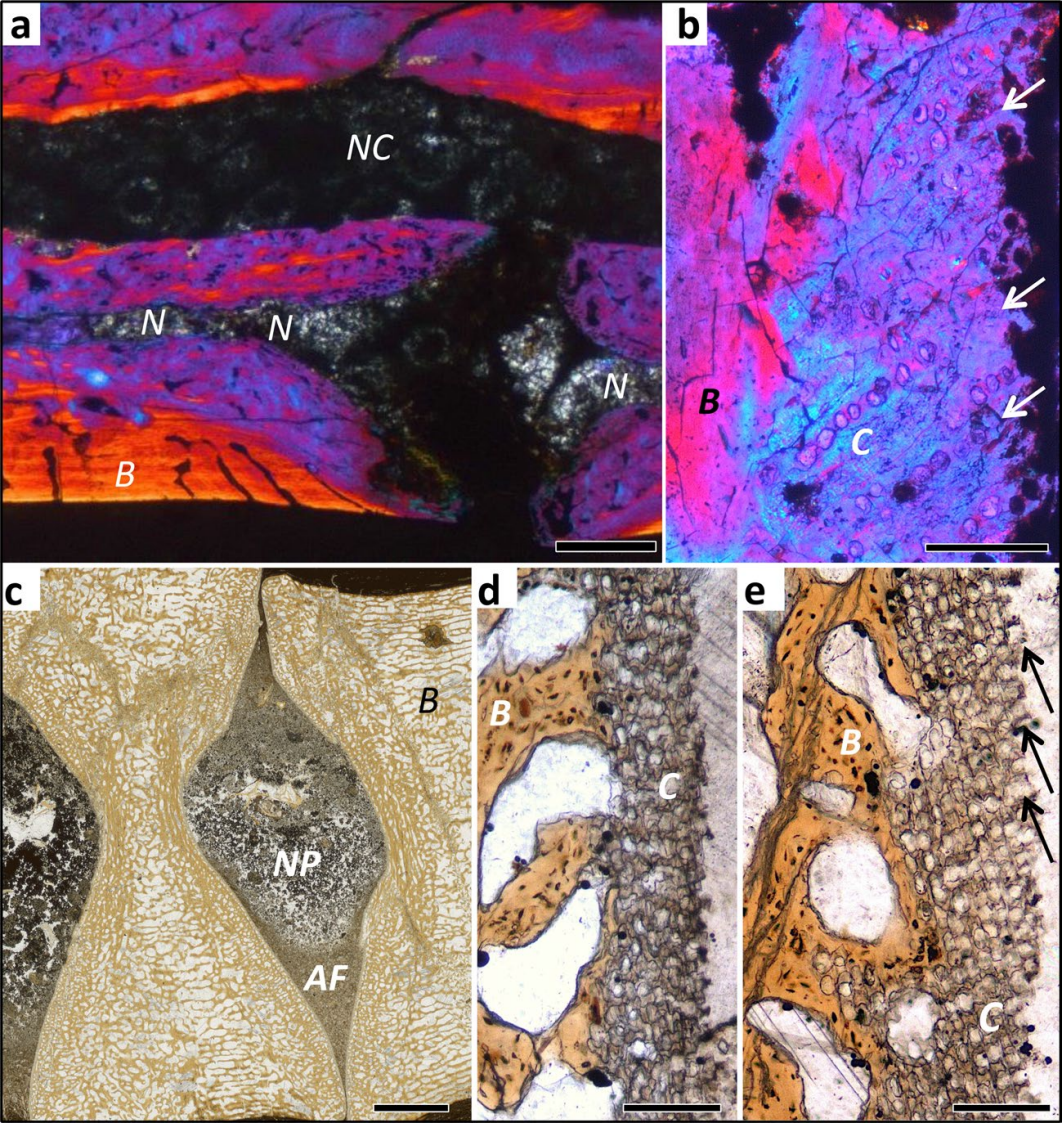
Histology of mesosaur, ichthyosaur and dinosaur intervertebral spaces, all fossil ground sections. (**a**) *Stereosternum tumidum* IGPB R 622, sagittal section of two articulated dorsal vertebral centra with intervertebral space, showing the notochordal amphicoelous shape and the persisting notochord. Image is in cross-polarised light with lambda filter. (**b**) Hadrosauridae indet. UALVP 59650. Close up of the articular surface showing obliquely arranged mineralised fibres in between poorly defined files of chondrocyte lacunae (arrows). Image is in cross-polarised light with lambda filter. (**c**) *Stenopterygius* sp. IGPB R 661 sagittal section of two articulated centra showing the amphicoelous shape. Note the differentiation of the content of the intervertebral space into a coarse into a fine fraction, probably representing the nucleus pulposus (NP) and the annulus fibrosus (AF). (**d**) Enlargement of the concave part of the articular surface, showing a thin layer of irregularly arranged chondrocyte lacunae, underlying the nucleus pulposus (NP). (**e**) Enlargement of the convex part of the articular surface, showing obliquely arranged files of chondrocyte lacunae, representing the insertion of the annulus fibrosus (AF) (arrows). Scale bar in (**a**) represents 500 µm, scale bar in (**b**) represents 100 µm, scale bar in (**c**) represents 2 mm, scale bars in (**d**, **e**) represent 100 µm. *AF* annulus fibrosus, *B* bone tissue, *C* cartilage, *N* notochord, *NC* neural canal, *NP* nucleus pulposus.

Using polarised light microscopy, we observed cartilage formed in the endochondral domain in all vertebral samples but in various altered states, of different kinds, and different distributions (Figs. 2, 3, 5; see “Methods” section). We also observed the fibrous insertion into the periosteal bone of the connective tissue of the joint capsule (of synovial joints) and the insertions of intervertebral ligaments. The distribution and arrangement of bone tissue and preserved hypertrophied cartilage, hyaline cartilage and fibrocartilage allows constraining the type of intervertebral joint and, in the case of the IVD, the size and location of the AF and NP. These were inferred from the distribution of outwardly inclined files of hypertrophied cartilage cells (Figs. 2a–c, 3c–e, see “Methods” section).

Especially in marine reptiles from black shales, we found soft tissue preservation across the intervertebral space (see “Methods” section), allowing further inferences about the nature of the joint connecting adjacent centra (Figs. 3c, S11, S19, S20). We also sampled extant taxa, in particular *Sphenodon*, for comparison with fossil soft tissues and to clarify contradictory interpretations in the literature on the nature of the intervertebral joints in this taxon (see Fig. S14a,b and SI). Turtles were excluded from the histological analysis because of the highly modified dorsals in the crown turtles, lacking intervertebral joints, and the lack of samples from stem turtles.

Based on the detailed descriptions of the histological samples (Supplementary Text), we are now able to review the relationships between vertebral centrum shape, observed fossilised and fresh tissues, inferred joint tissue types, and inferred joint type (Table 1, Fig. S29). The observed fossil tissues in the histological sections are bony tissues, altered cartilaginous tissues, and preserved soft tissues. We recognised five basic joint types in our sample (Table 1). (1) Amphicoelous, notochordal centra show a concave area with thin cartilage of irregularly arranged chondrocytes or smooth bone and a convex area with generally short chondrocyte files arranged in longitudinal direction with irregular bone spicules in between (Figs. S1–S4, S7, S14). We infer that these vertebral centra had an intervertebral joint consisting of an outer ring of fibrocartilage, best homologised with the AF, and an expanded notochord in the central region, as is observed in *Sphenodon* (Figs. 1, S14). This appears to be the plesiomorphic condition for amniotes which gave rise to all other joint types^10^. (2) Amphicoelous, non-notochordal centra show a concave area with thin or thick (several cells deep) irregular cartilage (but never bone), an outer area with chondrocyte files arranged in longitudinal direction with irregular bone spicules in between or long diverging files with an increasing angle of divergence (hupehsuchians, ichthyosaurs, placodonts) (Figs. 3c–e, S8–S13, S18). We infer that the taxa that show these features had a proper IVD with a NP of notochordal origin, supported by the preservation of an altered NP in some ichthyosaurs. Importantly, this type of morphology and joint is extinct. (3) Platycoelous centra, including those of extant mammals, show a central area with irregular cartilage and an outer area with diverging cartilage files and bone spicules (Figs. 2a–c, 3b, 5e–l, S5, S6, S19–S26). These two areas demarcate the AF and NP of the mammalian IVD, and we infer that fossil taxa with these features also had an IVD. (4) Procoelous and opisthocoelous vertebral centra that show a distinct layer of hyaline cartilage arranged in files normal to the bony surface (Figs. 2d–f, 5a–d, S15–S17), suggest the presence of a synovial joint with narrow joint cavity filled with synovial fluid, as observed in most extant squamates. (5) Procoelous and opisthocoelous vertebral centra of fossil taxa that show only irregular, thin (one or a few cells deep) cartilage and bone with fibre insertions (Figs. S27 and S28) suggest the presence of a fibrocartilage joint as in extant birds. We found evidence for intervertebral ligaments inserting in the periosteal bone in all types of joints.

**Figure 4:**
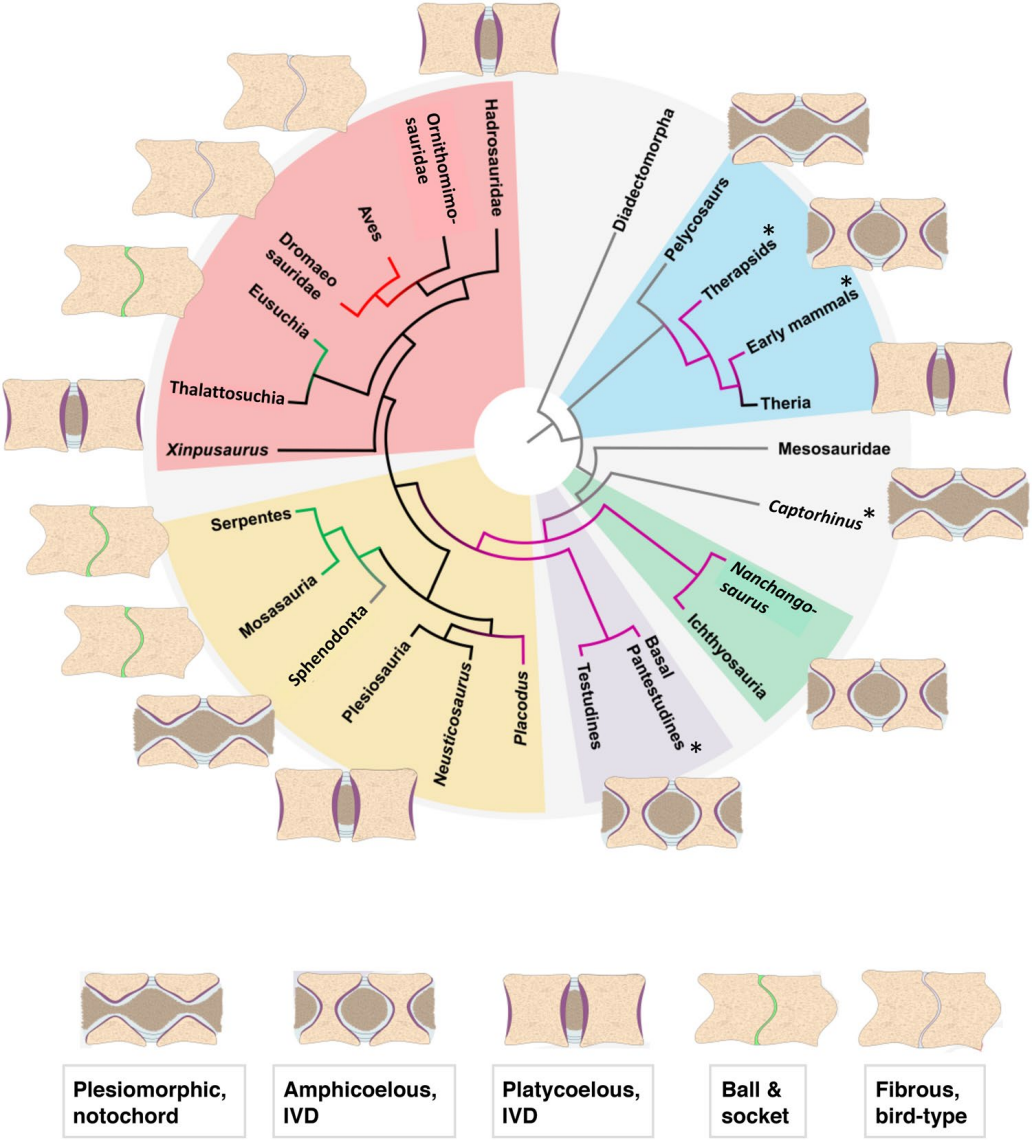
Ancestral state reconstruction using parsimony of the different types of dorsal intervertebral joints in the phylogeny of the higher clades of amniotes (see “Methods” and SM for details). The schematic drawings of the intervertebral joints are explained in the text. Grey branches indicate persisting notochord. Purple branches indicate an intervertebral disc between amphicoelous centra. Black branches indicate an intervertebral disc between platycoelous centra. Green branches indicate a synovial joint with hyaline cartilage in between procoelous or opisthocoelous centra. Red branches indicate a fibrous cartilage joint. Key to tissue colors: beige, bone; purple, cartilaginous endplate; light blue, AF; brown, NP; green, articular cartilage of synovial joint; grey, fibrous joint cartilage. Key to clade colors: light blue, Synapsida; light green, Ichthyosauria; light purple, Testudines; light yellow, Lepidosauromorpha; light red, Archosauromorpha. Asterisks indicate fossil taxa for which intervertebral soft tissue anatomy was inferred based on morphological and histological descriptions in the literature.

We then performed ancestral character state reconstructions (ASR)^8^ for a consensus amniote phylogeny, allowing us to trace the evolution of the amniote intervertebral joint (Fig. 4). Both parsimony (Fig. 4) and maximum likelihood-based ASR agree (Fig. S29). Looking at the tissue level, we explain the pathway of amniote vertebral joint evolution envisaged by Romer^1^ by combining fossil connective tissue evidence with principles of bone and cartilage formation and growth. The basal amniote joint, as exemplified by the iconic sail-backed synapsid *Dimetrodon* and the stem amniote *Diadectes*, must have contained a notochord constricted by the vertebral centrum and expanded in the space between the centra (Fig. 4). The vertebral centrum was a ring of bone of largely periosteal origin with a thin endochondral layer, modified from the condition in basal tetrapods^24^. In the joint, two such rings of bone were connected by a ring of fibrocartilage forming an AF, as seen in *Sphenodon* (Figs. 1, S14) and modern geckos^19^. The fibrocartilage inserted in the thin endochondral domain of each centrum and was covered on the outside by a ligamentous joint capsule consisting of intervertebral ligaments and inserting into the periosteal bone, as is seen in *Sphenodon* today (Figs. 1, S14). The AF and the intervertebral ligaments are the plesiomorphic condition for the holospondylous centra of amniotes. Next, the ring of bone closed up in its centre, resulting in a round aggregation of notochordal cells, the incipient NP. Because this stage is extinct, we cannot be sure of the exact nature of this incipient NP, i.e. whether it was the same structure as the NP in extant taxa or consisted of the remaining isolated pieces of the notochord. To a certain extent, this is a semantic issue. However, we found a probable altered NP preserved in ichthyosaur fossils from the Middle Triassic and Early Jurassic (Figs. 3c, S9, S11). The NP was separated from the endochondral bone surface by a layer of hyaline cartilage, the cartilaginous endplate (Figs. 3c–e, S11). At this stage, there still was little growth in the bony endplate, as indicated by the retention of the amphicoelous centrum. Closure of the notochordal canal happened convergently twice, once in therapsids and once in the reptile lineage, in early diapsids such as ichthyosaurs^25^ (Fig. 4).

From this stage evolved a platycoelous or slightly amphicoelous centrum with an IVD by greatly increased cartilage cell proliferation in the endochondral domain and subsequent endochondral ossification, filling in the space formerly occupied by notochordal tissue and fully developing it into an NP. The periphery of the articular surfaces remained connected by fibrocartilage of the AF. This evolutionary transition happened at least twice, in therian mammals and in higher diapsids (Fig. 4). Alternatively, the platycoelous centrum with an IVD evolved independently in eosauropterygians (a clade of marine reptiles to which plesiosaurs belong), and in archosauromorphs (Fig. 4), in addition to mammals. The platycoelous centrum with an IVD was then retained in most archosauromorphs, including basal crocodiliforms and most dinosaurs. Modern crocodiles evolved a synovial ball-and-socket joint convergently with lepidosaurs (Fig. 4). The evolutionary transition to the ball-and-socket joint of extant reptiles involved the reduction of the NP and the AF, i.e., the cartilage and notochordal components of the joint, and the development of a synovial space. The transition also involved strong anteroposterior asymmetry, with the formation of endochondral bone suppressed in the socket part of the joint and hypertrophied in the ball part, as seen in mosasaurs and snakes in this study (Figs. S15–S17, see SI).

The dinosaurs included in our study also retained the platycoelous centrum with the IVD except for the most derived non-avian dinosaurs, i.e., dromaeosaurs. In these, cartilage is greatly reduced, suggesting that the dorsal vertebral centra of these dinosaurs had the same fibrous connection as in extant birds.

## Discussion

Histological investigations of fossil hard tissues have become a powerful tool in palaeontology^26^, but in general they have been limited to bone and dental tissues. It is known that different soft tissue components of bone, such as osteons, bone collagen fibres, osteoblasts, and even blood vessels may preserve in deep time^27,28^. Apart from the bone, integumentary soft tissues may be preserved^5,29^ and, very rarely, internal organs^30,31^. It has long been understood that the anoxic conditions leading to the deposition of black shales permits the preservation of such soft parts, primarily of the integument, meaning that the potential exists that non-mineralised connective tissues will preserve. Hence, ideally, the articulated segments of vertebral column that we studied were still at least partially embedded in the matrix. This is important to be able to compare potentially preserved soft parts in the intervertebral spaces with the host sediment.

### Patterns of cartilage fossilisation

Cartilage fossilises well if it is mineralised, i.e., if the living chondrocytes mineralise in spherulitic arrangements of apatite crystallites, as in the case of calcified cartilage of chondrichthyans. However, fossilisation of cartilage has been little studied, and any evidence of cartilage in a fossil traditionally has been subsumed by palaeontologist unter the term "calcified cartilage”, even if the tissue in question was not mineralised in life (e.g., Ref.^32^). There is true calcified cartilage in extinct amniotes, i.e., serial cartilage that was not replaced by bone but was fully mineralised in the living animal. An example is provided by some pachypleurosaurs, small Triassic sauropterygians, in which these tissues served to increase skeletal mass^33,34^. The term calcified cartilage, however, is also used for the zone of hypertrophy of chondrocytes in the growth plate of amniote long bones^35^. This cartilage is replaced completely by endochondral bone during the growth phase of the animal, and its presence suggests immaturity. Whereas the hypertrophy zone may show increased mineralisation^35^, favoring fossilisation, the chondrocytes do not show the characteristic spherulitic radial crystallite arrangement of true calcified cartilage. We note that true calcified cartilage was not encountered in this study.

### Patterns in intervertebral joint evolution

The convergent evolution of the IVD in synapsids and reptiles by closure of the notochordal canal and evolution of an NP from the notochord had remained unrecognised for two reasons: First, the general belief was that amphicoelous vertebrae did not house an IVD based on the only extant amphicoelous amniotes, i.e., *Sphenodon* and some geckos. We now recognise the presence of an IVD in extinct amphicoelous reptiles, e.g., in ichthyosaurs, hupehsuchians, probably non-mammalian therapsids, and possibly stem tutles (Fig. 4). The condition in these taxa is different from the plesiomorphic condition of a continuous notochord. The second reason is that reptile clades that had evolved an IVD went extinct at the end of the Cretaceous, after giving rise to lineages with synovial ball-and-socket joints and bird-type intervertebral joints (not proper IVDs with a NP). However, we consider it unlikely that these types of joints were the decisive factor in survival of this mass extinction. Synovial ball-and-socket joints also evolved in Jurassic sauropodomorphs dinosaurs^36^ and in some non-avian theropod dinosaurs^37^, all of which went extinct at the end of the Cretaceous. Note that in some recent studies^3,38^, the term ‘avian IVD’ or similar is used. This has created confusion because in these same studies, it is stated or illustrated that the avian ‘IVD’ lacks a NP and is not homologous to the IVD proper. Given the complex distribution of the different types of intervertebral joints on the amniote tree, further studies on well-preserved fossils in combination with soft tissue histology of extant taxa and developmental studies hold great potential^10^. The evolutionary scenario presented here results in hypotheses that are testable using classical embryology and developmental genetics.

## Conclusions

Preserved soft tissues, bone histology and articular surface morphology inform on the nature of the intervertebral articulation in fossil amniotes. Currently, this understanding is restricted to dorsal vertebrae, and the approach should be extended to cervical vertebrae in particular. Remains of articular cartilage are regularly present in fossils and can be observed in palaeohistological thin sections. The shape and organisation of the chondrocytes can be used to infer presence and size of an AF and NP. Occasionally, remains of the notochord and the NP also are encountered in such sections. The preserved tissues lead us to infer that amphicoelous, notochordal vertebrae had an intervertebral joint consisting of a continuous notochord surrounded by a peripheral AF. This is the plesiomorphic amniote type and must have been present in stem amniotes, basal synapsids, and basal reptiles. It is also seen *Sphenodon* and some geckos today. We also infer that amphicoelous, non-notochordal vertebrae were connected by an intervertebral disc with a NP surrounded by an AF. This was the situation in hupehsuchians, ichthyosaurs, placodonts, probably non-mammalian synapsids, and possibly stem turtles. Fossil platycoelous vertebrae possessed a proper IVD resembling that of extant mammals. Among reptiles, a proper IVD must have been present in eosauropterygians and most archosauromorphs, including most non-avian dinosaurs. However, this reptilian IVD went extinct at the end of the Cretaceous with the extinction of non-avian dinosaurs and plesiosaurs Finally, ancestral state reconstruction on a consensus phylogeny of amniotes using maximum parsimony and maximum likelihood algorithms in the software Mesquite indicates that the intervertebral disc evolved convergently at least twice, once on the mammalian stem line and once in early diapsids. Similarly, a ball-and-socket joint evolved at least twice convergently.

## Methods

### Acquisition of histological and morphological data

For this study, we histologically sampled 22 specimens representing 19 taxa of different amniote clades which include mammalians, non-mammalian synapsids, and reptiles (including non-avian dinosaurs and birds) (see Table S1). We used histological ground sections (for fossil taxa), histological demineralised sections (for extant taxa) and morphological data, i.e., the shape of the bony articular surface of the vertebral centra (see Table 1). For the stem reptile *Captorhinus*, we used the histological images in Ref.^32^. For the morphological part, we checked the general applicability of our observations with information from the rich literature on amniote vertebral centrum morphology, including another three taxa for which morphological (non-mammalian therapsids, basal mammals) and also histological information (*Alligator*) is available. Extant turtles are a special case because they lack mobile intervertebral joints between the dorsal vertebrae which are integrated into the carapace. The stem turtles *Pappochelys*, *Odontochelys*, and *Eorhynchochelys* lack a carapace and have non-notochordal amphicoelous vertebrae^39–41^. No histological data are available for these taxa, but we included the published morphological information in some analyses.

### Sampling and histology of fossil and extant non-decalcified material

Two types of fossil material were sampled for this study. First, segments of the dorsal vertebral column of articulated skeletons as well as isolated but articulated segments of at least two centra. In the latter, we focused on specimen in which the vertebrae are preserved in close articulation. These specimens originally also pertained to articulated skeletons that were collected in an incomplete state, however. The incomplete state presumably is due to loss to weathering before discovery. The second type is isolated vertebral centra that were sampled if articulated column segments were not available.

Articulated column segments are primarily found in conservation deposits such as black shales (e.g., the Posidonienschiefer Formation of southern Germany), but isolated vertebrae derive from a host of different sediment types. The segments of vertebral column and isolated vertebrae were sectioned as exactly in the sagittal plane as possible. In the case of longer segments (e.g., *Mesosaurus*, *Stenopterygius*, *Neusticosaurus*, etc.), there was some degree of curvature of the vertebral column, but we attempted to intersect at least two adjacent centra in the exact sagittal plane. The half of the segment that represented the closest approximation to the sagittal plane was then processed into a petrographic thin section following standard methods^26^. Thin sections were ground to a thickness of 50–80 µm, depending on the degree of dark staining. Thin sections were then examined with a standard polarising microscope, either a Leica DLMP or a Zeiss Axio Imager und normal and cross-polarised light with and without a lambda filter. Images were taken with a Leica digital camera and processed with ImageAccess EasyLab 7 software.

**Figure 5:**
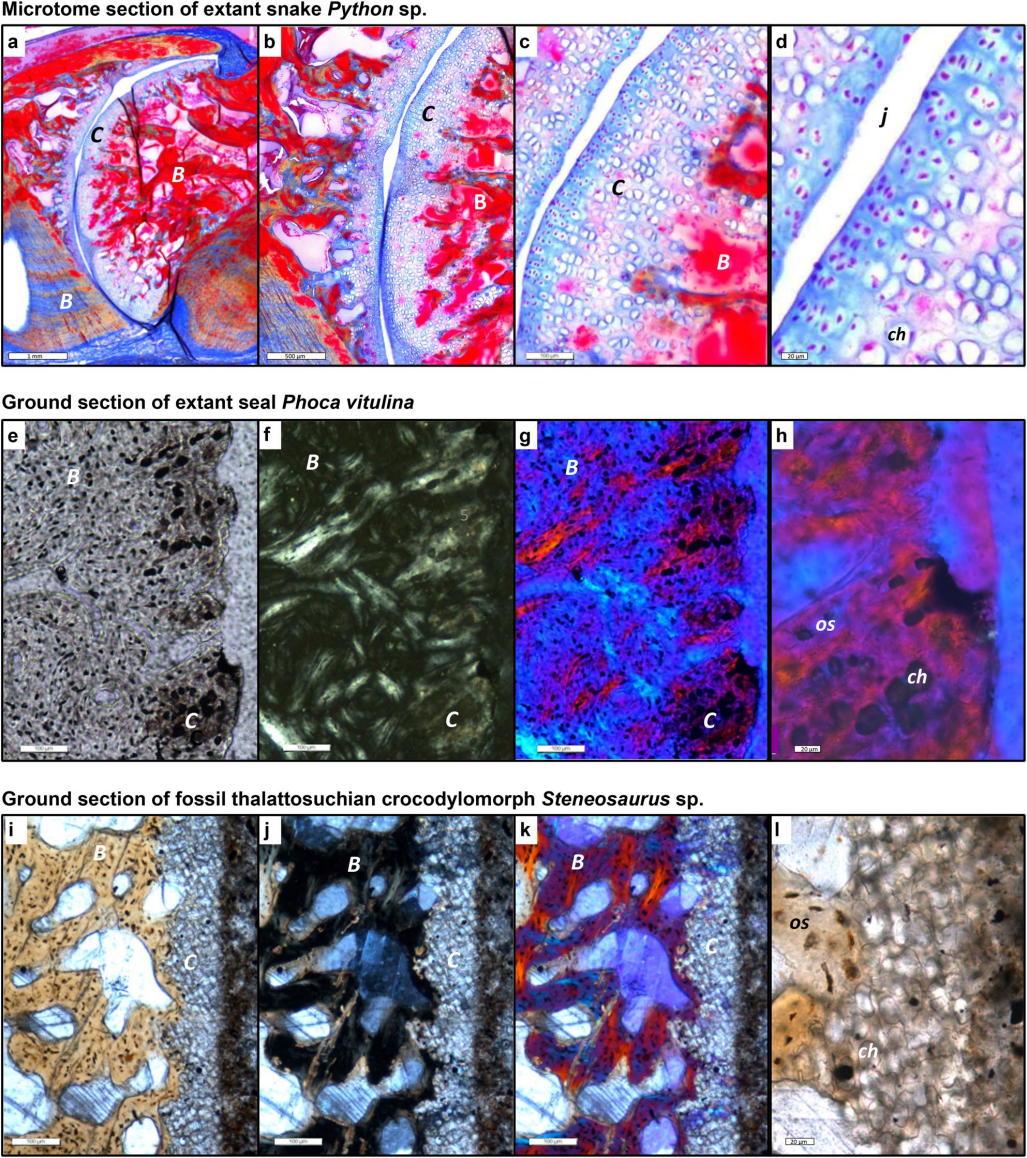
Intervertebral articular cartilage in extant and fossil amniotes and different types of sectioning and light microscopy. (**a**–**d**) Microtome section stained with Azan of extant joint with cartilage of extant snake *Python* sp. IGPB R 662, synovial ball-and-socket joint at increasing magnifications in normal transmitted light. Blue is cartilage and other connective tissues, red is bone. Note the serial cartilage, i.e., the arrangement of the cartilage cells in files, especially in (**c**). (**d**) Cartilage layer with differentiating (left) and hypertrophied (right) cartilage cells, note the size increase from left to right and the globular shape of the hypertrophied chondrocytes, as in (**h**) and (**l**). (**e**–**h**) Ground section of extant seal *Phoca vitulina* IGPB M 60, joint with IVD, peripheral area of articular surface. (**e**) Section in normal transmitted light. (**f**) Section in cross-polarised light. (**g**) Cross-polarised light and lambda filter. (**h**) Close-up of **g**. (**i**–**l**) Ground section of fossil thalattosuchian crocodilomorph *Steneosaurus* IGPB R 663, joint with IVD, peripheral area of articular surface. (**i**) Section in normal light. (**j**) Section in cross-polarised light. (**k**) Cross polarised light and lambda filter. (**l**) Close-up of (**i**). Note that the fossil cartilage does not show the same birefringence as the bone. *B* bone tissue, *C* cartilage, *ch* chondrocyte, *j* joint space, *os* osteocyte.

Dental tissues and bone fossilises very well at the histological level, and observations using light microscopy are directly transferrable from fossil to extant comparative material^26,35^. Histological descriptions of fossil hard tissues accordingly use the same terminology as for extant amniotes, and this terminology is discussed in detail by Francillon-Vieillot et al.^35^. In normal light, all types of bone tissue (periosteal, endochondral, secondary) show a brownish stain of the matrix developed during fossilisation^28^. Osteocyte lacunae are generally visible in this bone matrix. Remains of cartilaginous tissues, on the other hand, generally lack the brownish stain, and are whitish or light greyish translucent (Fig. 5). In polarised light, bone is easily distinguished from any kind of cartilage as well because of the birefringence of the bone apatite crystallites. Cartilage and other soft tissue remains lack birefringence or show the birefringence of the templating mineral, e.g., calcite (Fig. 5).

Although extant material is typically studied in decalcified microtome sections, ground sections (petrographic thin sectioning) of fresh bone can be studied as well. The advantage is a better identification of the bone tissue types because decalcification destroys the birefringence of the bone tissue. In addition, the observational relationship between bone, cartilage, and connective tissue is the same as in fossils, offering an intermediate in the comparison between fossil ground sections and microtome sections. We studied some extant material using this technique (Table S1).

### Microtome histology of extant material

Two taxa (*Sphenodon* and *Python*) were successfully sampled by decalcified microtome section in the sagittal plane of the dorsal and caudal region of the vertebral column from consecutive vertebrae (Table S1). The *Sphenodon* specimen used for sectioning is from the original collection of Reischek made in 1890 for the NHMW. Because it has been stored in ethanol for more than a century, the material was extremely desiccated and tough and therefore difficult to section with the microtome. After several washes in ethanol, rehydration and refixation with 4% paraformaldehyde, the samples were demineralised for 50 days in 8% EDTA at pH 8. After re-transfer into ethanol, the samples were embedded in paraffin following standard procedures. For sectioning on a standard microtome at 10 µm, special blades for hard tissue were used (N35HR microblades, pfm medical). After mounting on silane-coated glass slides, the sections were treated by HE staining and Heidenhain Azan staining according to standard procedures.

The *Python* samples were generated from fresh frozen material from a historical collection at the IGPB. The muscular tissue was partially removed from the vertebral column without damaging the vertebral ligaments. After fixation for 7 days in 4% PFA, the samples were washed and demineralised for 33 days in 8% EDTA at pH 8. Whole-mount preparations were cut with a razor blade in the median plane. Samples for histological sections were embedded in paraffin, sectioned at 10 µm with a standard microtome and treated by HE staining and Heidenhain Azan staining according to standard procedures. Note that ground sections in polarised light may appear in a similar color scheme to stained microtome sections (e.g., Figs. 2, 5), but the meaning and origin of these colors is radically different.

### Preservation of cartilage and inferences on intervertebral tissues in fossils

Our ground histological sections of fossils allow different observations and inferences, depending on the quality of preservation, about the nature of the tissues occupying the space between the bony end plates of successive vertebrae (Fig. 1, Table 1). These inferences are based on the principles of vertebral centrum and joint differentiation and growth (see “Introduction” and SI). All of the isolated vertebral centra in our study preserve histological evidence for the nature of the cartilage covering the bony end plate, including cartilage preserved in different states of alteration by fossilisation and of lacunae that once housed the chondrocytes. Although frequently noted in palaeohistological descriptions (e.g., Ref.^32^), fossil cartilage has been little studied. The identification as fossil cartilage is based on the direct comparison with cartilage in extant animals. Just as fossilised bone tissue can be directly compared with living bone tissue in histological ground sections (e.g., Ref.^35^), so can fossilised cartilage (Figs. 2, 3, 5). Cartilage cells and extracellular matrix may either have become mineralised during fossilisation or leave empty spaces surrounded by bone matrix and later filled in by diagenetic minerals. Both, cell size and cell shape are readily comparable with those in fresh cartilage, and the different types of cartilage produced by the chondroblasts can be observed (Fig. 5).

Bone tissue and fossilised hypertrophied cartilage of the endplate also offer histological correlates of other intervertebral soft tissues (Fig. 1, Table 1). Thus, the direction and presence/absence of files of cartilage cells (chondrocytes) and anchoring fibres in the bone allow mapping of the AF and NP or notochordal tissue (Fig. 1). The arrangement of the chondrocytes in files results from the directed growth of the cartilage in rows by chondrocyte division, hence the term ‘serial cartilage’ (Table 1). This mapping approach is based on the numerous descriptions and illustrations in the literature and our own observations (e.g., Fig. 1). Human IVDs (e.g., Ref.^2^) and other mammalian IVDs (e.g., Ref.^42^, Fig. 2, and Ref.^43^, Fig. 7) are abundantly figured in the medical literature (although not always correctly labelled). Our own observations cover the histology of humans and other extant mammals (e.g., *Phoca vitulina,* see Figs. 5, S5, S6, SI) and reptiles (e.g., *Sphenodon punctatus*, see Fig. 1 and Fig. S14, and *Phython* sp.; see Figs. 2, 5, and Figs. S16). *Sphenodon* is particularly important because it represents the only extant example of the ancestral condition of the amniote intervertebral joint (Figs. 1, 4).

Our observations and literature search indicate that the hypertrophied cartilage cells are organized into files (serial cartilage) in the region of attachment of the annulus fibrosus (Fig. 1b, Table 1). The inclination of the files relative to the bony endplate (or articular surface) increases outwards, reflecting the orientation of the collagen fibres in the fibrocartilage of the AF (Fig. 1b). On the other hand, the central region of the joint, the location of the NP or notochordal tissue, is only underlain by loosely organized hypertrophied cartilage (Fig. 1e). Two types of anchoring fibres are also preserved in the fossils. In fact, they are commonly better visible in fossils than in ground and particularly microtome sections of extant vertebrae. The optical birefringence is not as well developed in fresh bone as in fossil bone, and not all stains of microtome sections pick up collagen fibres (e.g., they are not seen in the HE-stained *Sphenodon* sections (Fig. 1c). One fibre type is the classical Sharpey’s fibres that insert in the periosteal bone near the anterior and posterior end of the centra (Fig. 5a,b), indicating the location of the joint capsule formed by intervertebral ligaments (Figs. 1c, S16b). Note that The other types are fibres that insert in the endochondral bone and hypertrophied cartilage, anchoring the AF to the end plate (Figs. 1b, 5). Their distribution and angle of insertion thus also can be used to infer the size and structure of the AF (Fig. 1b, Table 1). For details, see the description of the individual specimens in the SI and Figs. S1–S28.

### Preservation of non-mineralised skeletal and connective tissues

It has long been understood that the anoxic conditions leading to the deposition of black shales permit the preservation of soft parts, primarily of the integument, meaning that the potential exists that non-mineralised connective tissues will preserve (e.g., Ref.^44^). We sectioned articulated segments of vertebral column from black shale settings which appear to preserve altered or templated joint connective tissues other than hyaline cartilage, i.e., the fibrocartilage of the AF and the cells of the NP. Hence, ideally, the articulated segments of vertebral column that we studied were still at least partially embedded in the matrix. This is important to be able to compare potentially preserved soft parts in the intervertebral spaces with the host sediment. For details, see the description of the individual specimens in the SI and Figs. S1–S28.

### Optimization of intervertebral articulation on the amniote tree

For the reconstruction of the evolution of the intervertebral articulation in amniotes, we used ancestral character state reconstruction (ASR)^8^. We built a consensus tree of the amniotes sampled based on the literature, including 24 tip taxa. The phylogeny is based in most parts on Müller et al.^45^ and Chen et al.^46^ and is consistent with a new analysis of mesosaur relationships^47^. The phylogenetic position of thalattosaurs, represented in our study by *Xinpusaurus*, varies between basal neodiapsid affinities^45^, basal lepidosauromorph affinities^48^, as part of a clade including hupehsuchians and ichthyosaurs^46^, and the traditional view of basal archosauromorph affinities which we expressed in Fig. 4 and Fig. S29a. We implemented this uncertainty in different ASRs but did not find any influence on the outcome to the overall analysis (Fig. S29).

Commonly turtles are considered archosauromorphs, if not archosaurs, a view based on molecular evidence, which does indeed point towards archosauromorph affinities^45^. However, current morphological phylogenetic analysis including transitional fossil overwhelmingly recover basal diapsid or lepidosauromorph affinities^39,41,50^. Note that these morphological results were obtained independently by three different teams, without any overlap in authorship in the last four years, postdating molecular analyses. This is why we place turtles as basal Diapsida (Fig. 4 and Fig. S29)^39,50^. Because the phylogenetic position of turtles remains controversial beyond their diapsid affinities and because no histological data are available for stem turtle vertebral centra, we ran the ASR both with and without turtles to test whether their inclusion nevertheless might have an effect on the outcome of the ASR. In the analysis including turtles, we placed them in the least nested position on the diapsid stem (Fig. 4), but more derived than Ichthyosauria, and we coded their vertebrae as non-notochordal amphicoelous^37–39^. We recognised five character states describing the different types of intervertebral joints based on our morphological and histological observations and inferences. These character states are as follows and are described in Table 1: 0, amphicoelous centrum with notochordal canal, continuous notochord; 1, non-notochordal amphicoelous centrum with IVD; 2, platycoelous centrum with IVD; 3, synovial ball-and-socket joint; 4, fibrous ball-and-socket joint. Data for non-mammalian Therapsida, early mammals, and Eusuchia were taken from the literature (Table S1). We then entered the consensus phylogeny and the states for the tip taxa into a NEXUS file (Supplementary NEXUS file 1) and analyzed it in Mesquite v. 3.0243^51^ using both maximum parsimony (MP) and maximum likelihood (ML). For the ML reconstruction, we used the one-parameter Markov k-state probability model. The inclusion of turtles had no effect on the analysis, and we excluded them from further consideration.

## Supplementary information

Supplementary file 1

Supplementary file 2
