# Supplementary material for: Palaeontological evidence reveals convergent evolution of intervertebral joint types in amniotes
Source: Sci Rep. 2020 Aug 24;10:14106. doi: 10.1038/s41598-020-70751-2 (PMC7445751; doi:10.1038/s41598-020-70751-2)
Supplement: Supplementary file 1 [file 41598_2020_70751_MOESM1_ESM.pdf]

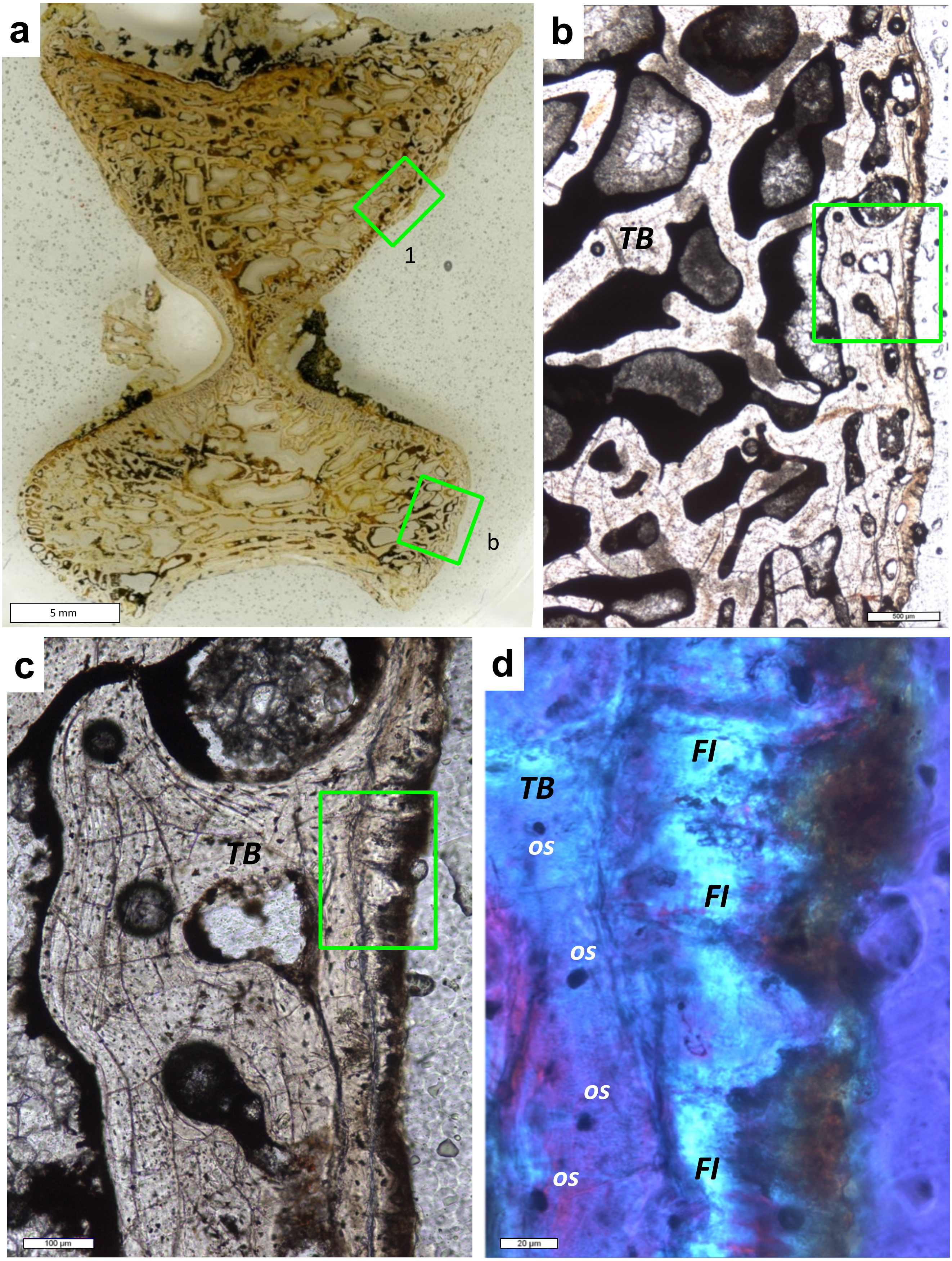

Figure S1. Histology of *Diadectes sideropelicus* IGPB A169, joint with persisting notochord. a Sagittal section of vertebral centrum, neural canal is to the top.1, location of Figure S2a. **b** Enlargement of peripheral part of articular surface in plane polarised light. **c** Closeup of **b**. **d** Close-up of **c** in cross-polarised light and lambda filter. Note the uneven surface and fibre insertions. FI, fibre insertions; os, osteocyte; TB, trabecular bone tissue.

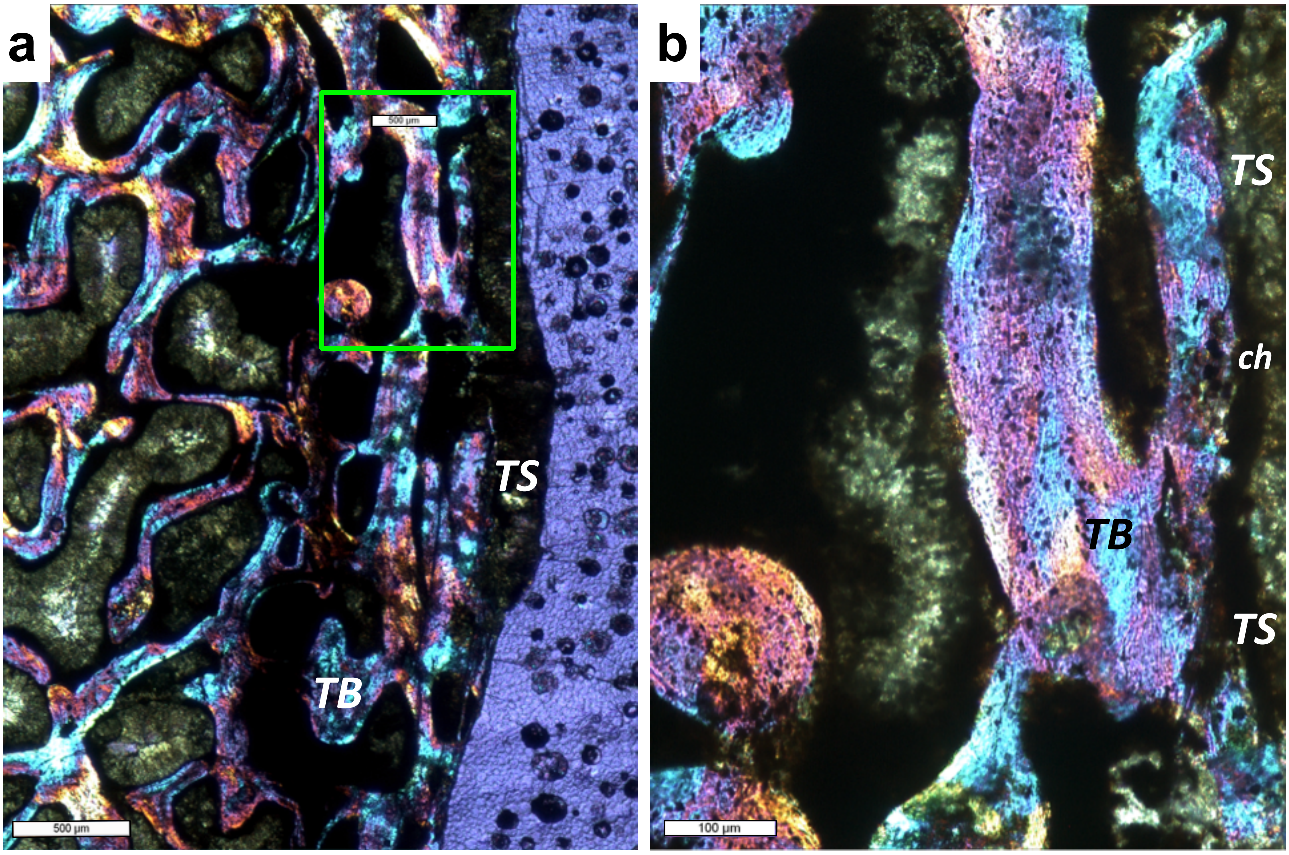

Figure S2. Histology of *Diadectes sideropelicus* IGPB A169 continued. a Inset 1 in Figure S1a, showing the inner part of the articular surface in cross-polarised light and lambda filter. **b** Closeup, note the smooth trabecular surface facing the intervertebral cavity, the scarcity of chondrocyte lacunae, and the lack of fibre insertions. ch, chondrocyte lacunae; TB, trabecular bone tissue; TS, trabecular surface.

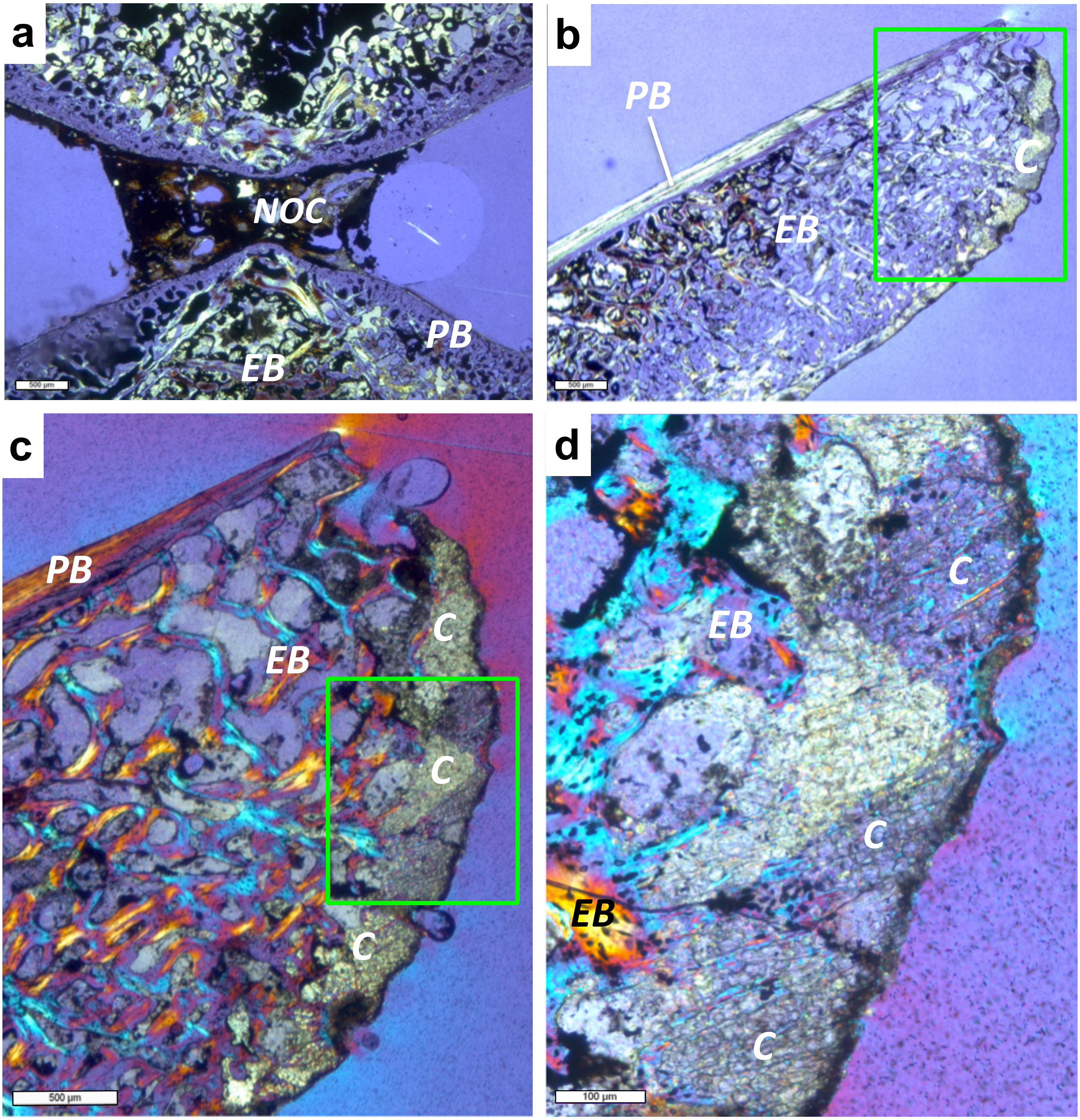

Figure S3. Histology of *Dimetrodon natalis* juvenile IGPB R 652a, joint with persisting notochord. a Sagittal section of the middle of the vertebral centrum, intersecting a large notochordal foramen. Neural canal is to the top. **b** Sagittal section of the outer part of the vertebral centrum showing a thick layer of fossilised cartilage on top of the endochondral trabeculae. **c** Closeup of **b**. **d** Closeup of **c**. Note the chondrocyte lacunae arranged in obliquely oriented files and the intervening ossified fibres (blue birefringent streaks in the cartilage). All images in cross-polarised light and lambda filter. C, cartilage; EB, endochondral bone; NOC, notochord canal; PB, periosteal bone tissue.

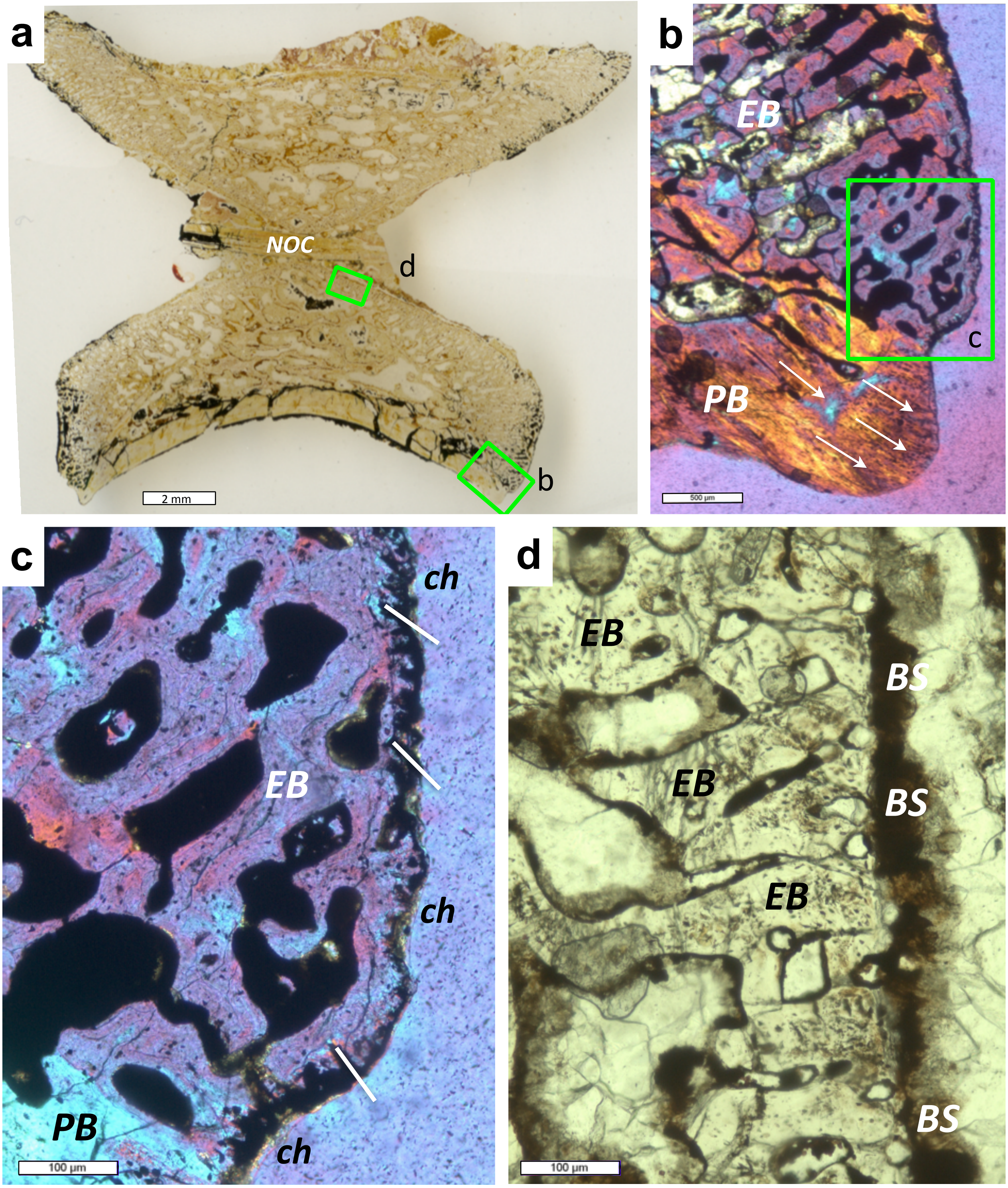

Figure S4. Histology of *Dimetrodon natalis* adult IGPB R 653a, joint with persisting notochord. a Sagittal section of vertebral centrum, neural canal is to the top. **b** Enlargement of peripheral part of articular surface (area b) in cross polarised light and lambda filter. **c** Closeup of **b**. Note the uneven surface and fibre insertions. **d** Close-up of area b in plane-polarised light. Note the smooth surface facing the intervertebral cavity and the lack of chondrocyte lacunae. Arrows denote Sharpey's fibres inserting in the periosteal bone, white lines indicate the direction of the files of chondrocytes in the serial cartilage of the endplate overlying the endochondral bone. BS, surface of endochondral bone; ch, chondrocyte lacunae; EB, endochondral bone; NOC, notochord canal; PB, periosteal bone tissue.

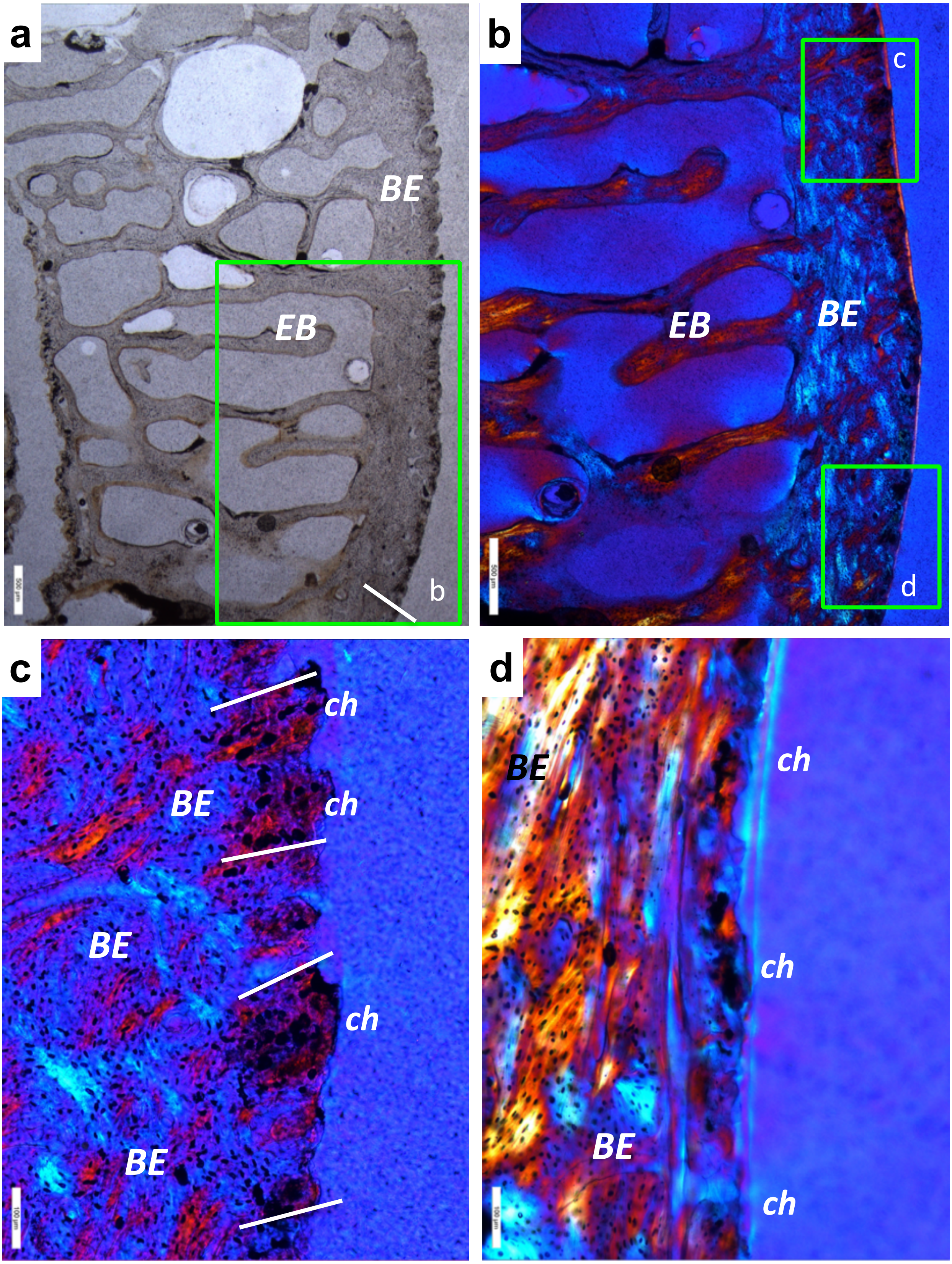
a
BE
EB
b
b
c
EB
BE
d
c
ch
BE
ch
BE
ch
BE
d
BE
ch
ch
BE
ch

Figure S5. Histology of *Phoca vitulina* IGPB M 60, joint with IVD. a Close-up from sagittal section of vertebral centrum, showing part of the bony epiphysis. **b** Enlargement of articular surface (area b) showing the transition from the smooth inner part of the articular surface to the peripheral rough surface. **c** Closeup of the rough peripheral surface. Note the oblique orientation of the files of chondrocyte lacunae (black) and the fibre insertions. White lines indicate the direction of the files of chondrocytes in the serial cartilage of the endplate overlying the endochondral bone. Files are separated by spicules of fibrous bone (high birefringence). **d** Close-up of the smooth inner surface. Note rarity and poor organisation of the chondrocyte lacunae. Image **a** in plane polarised light, image **b-d** in cross polarised light and lambda filter. BE, bony endplate of epiphysis consisting of endochondral bone; ch, chondrocyte lacunae; EB, endochondral bone; PB, periosteal bone tissue.

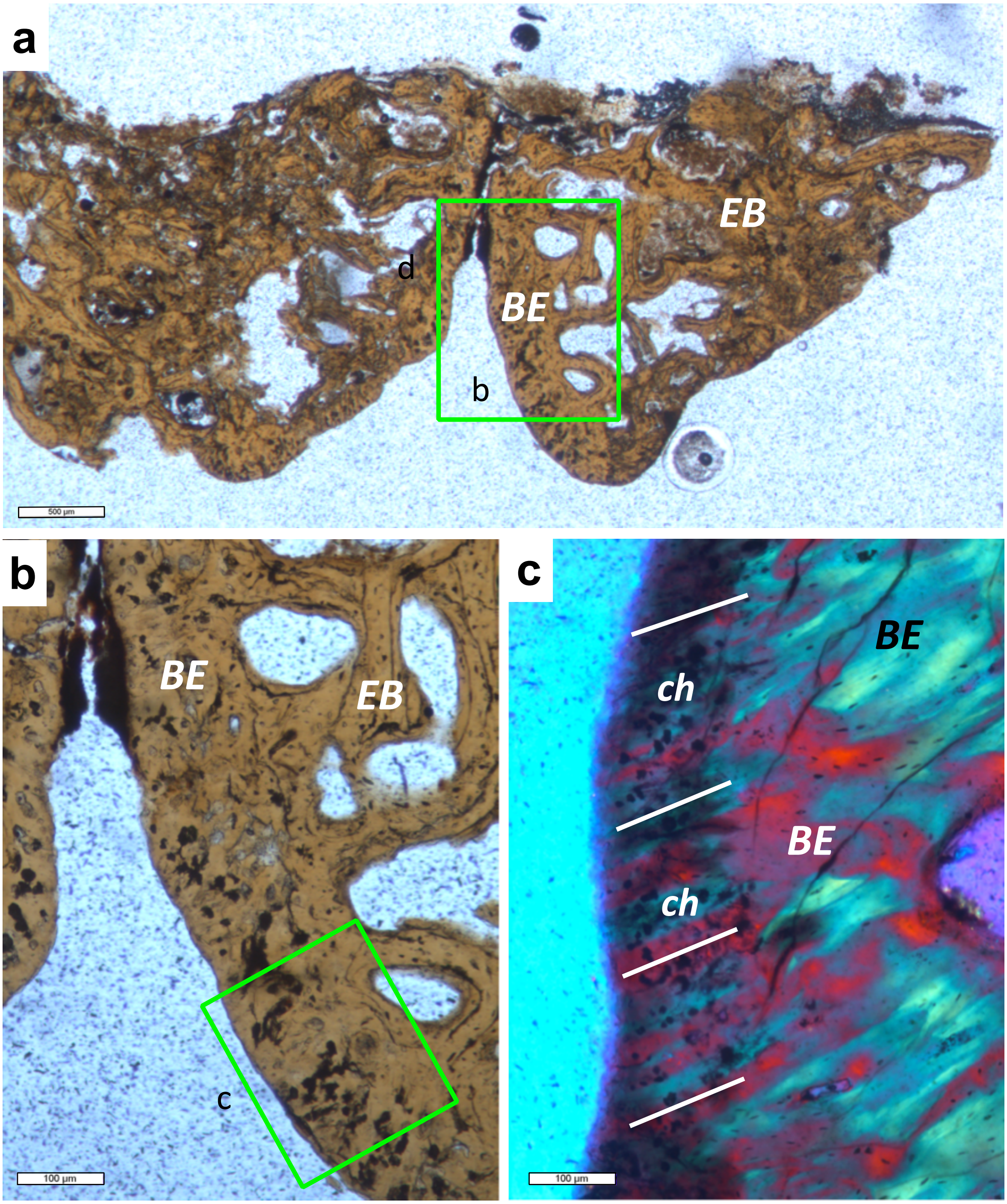

Figure S6. Histology of *Eurohippus* sp. HLMD ME 139, joint with IVD. a Sagittal section of the outer margin of the articular surface of two articulated vertebral centra in plane polarised light. **b** Enlargement of peripheral part of articular surface in plane polarised light. Note the outwardly directed files of chondrocyte lacunae. **c** Closeup in cross-polarised light and lambda filter. Note the fibre insertions between the chondrocyte files (white lines). BE, bony endplate of epiphysis consisting of endochondral bone; ch, chondrocyte lacunae; EB, endochondral bone; PB, periosteal bone tissue.

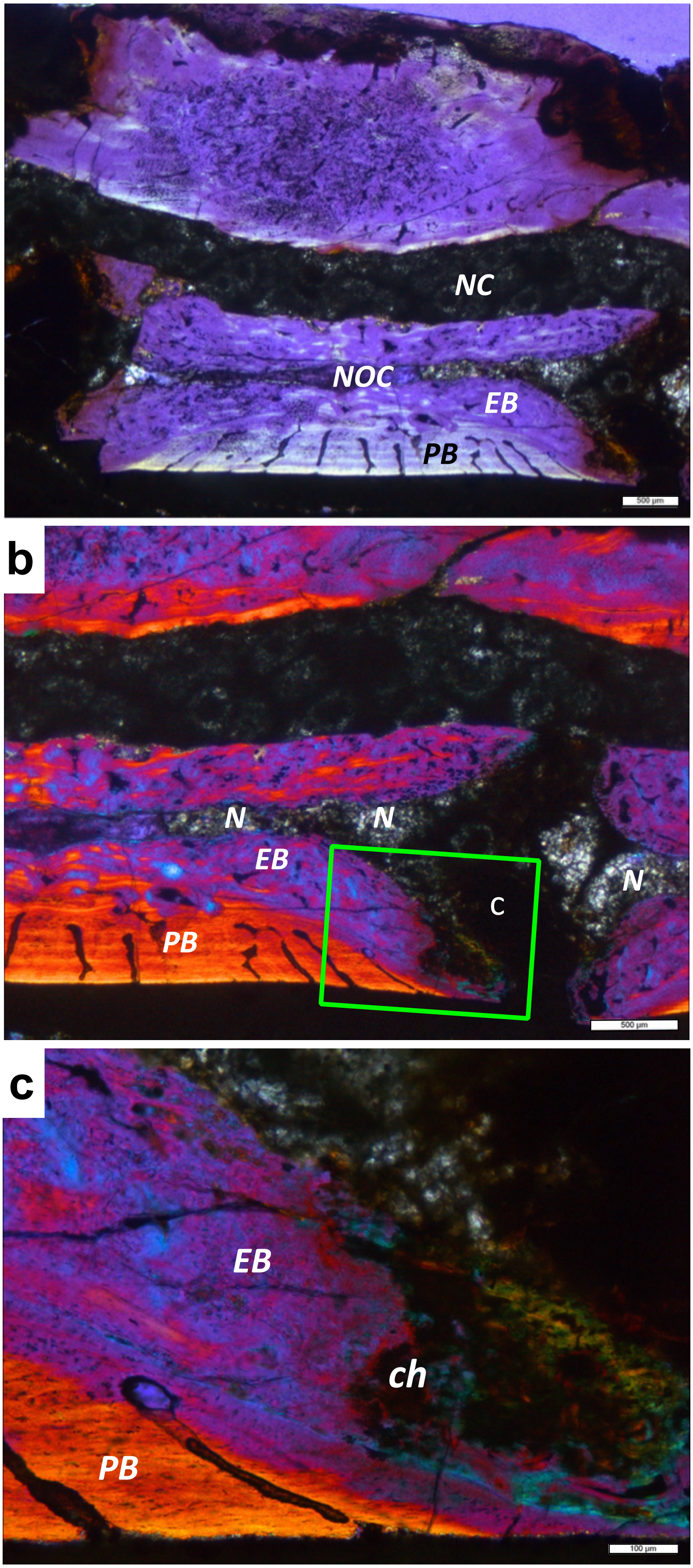

Figure S7. Histology of *Stereosternum tumidum* IGPB R622, joint with persisting notochord. a Sagittal section of a segment of vertebral column, neural canal is to the top. Image in plane polarised light. **b** of the joint area of two articulated vertebral centra in cross-polarised light and lambda filter. Note the continuous notochordal canal. **c** Closeup of **b**. Note the uneven surface and fibre insertions in the peripheral area. ch, chondrocyte lacunae; EB, endochondral bone; N, notochord; NC, neural canal; NOC, notochord canal; PB, periosteal bone tissue.

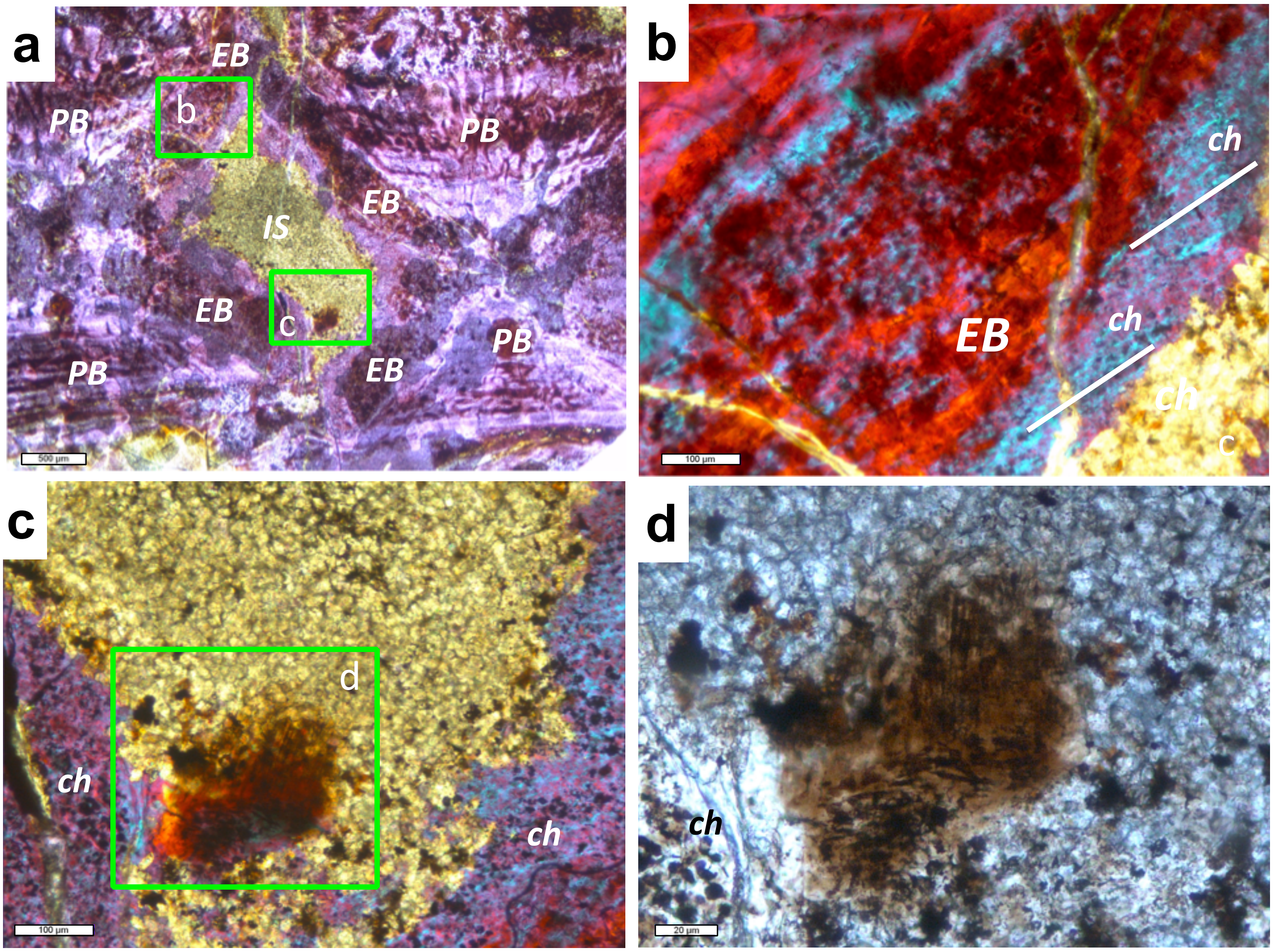

Figure S8. Histology of *Nanchangosaurus* HFUT YAN-10-02. **a** Sagittal section of two articulated vertebral centra in cross-polarised light with lambda filter. **b** Enlargement of "funnel" part of articular surface. Note the outwardly directed files of unmineralised chondrocyte lacunae (blue and pink) followed by a thick layer of endochondral bone with cartilage (mainly red) and periosteal bone (upper left). White lines indicate the direction of the files of chondrocytes and anchoring fibres in the serial cartilage overlying the endochondral bone. **c** Closeup of ventral part of articular space in cross-polarised light with lambda filter. Note the patch of brown fibrous tissue and the granular nature of the matrix. **d** Closeup of brown fibrous tissue and the granular matrix in plane polarised light. ch, chondrocyte lacunae; EB, endochondral bone; IS, intervertebral space; PB, periosteal bone tissue.

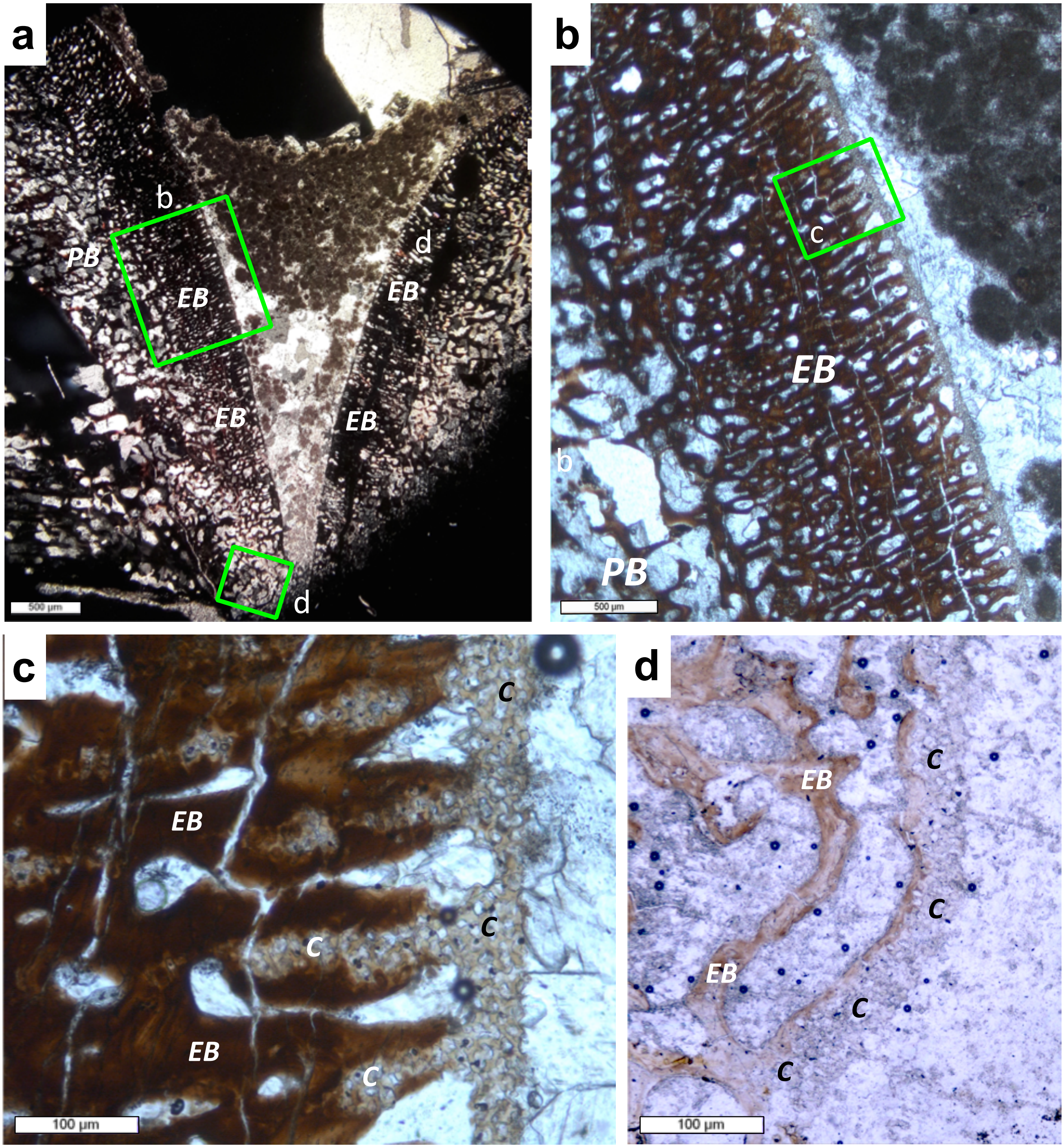

Figure S9. Histology of Cymbospondylidae new taxon A LACM DI 158109, joint with IVD. a Sagittal section of the ventral part of two articulated vertebral centra and intervening matter. Neural canal is to the top, image in cross-polarised light. **b** Enlargement of **a** showing layer of fossilised cartilage on top of the endochondral trabeculae and the globular bodies of the possible NP. Image in plane polarised light. **c** Closeup of **b**. Note the chondrocytes arranged in files (hypertrophy zone) and the replacement of the chondrocytes by endochondral bone. Image in plane polarised light. **d** Closeup of the margin of the articular surface. The arrangement of chondrocyte lacunae into files is indistinct. Image in plane polarised light. EB, endochondral bone; PB, periosteal bone tissue.

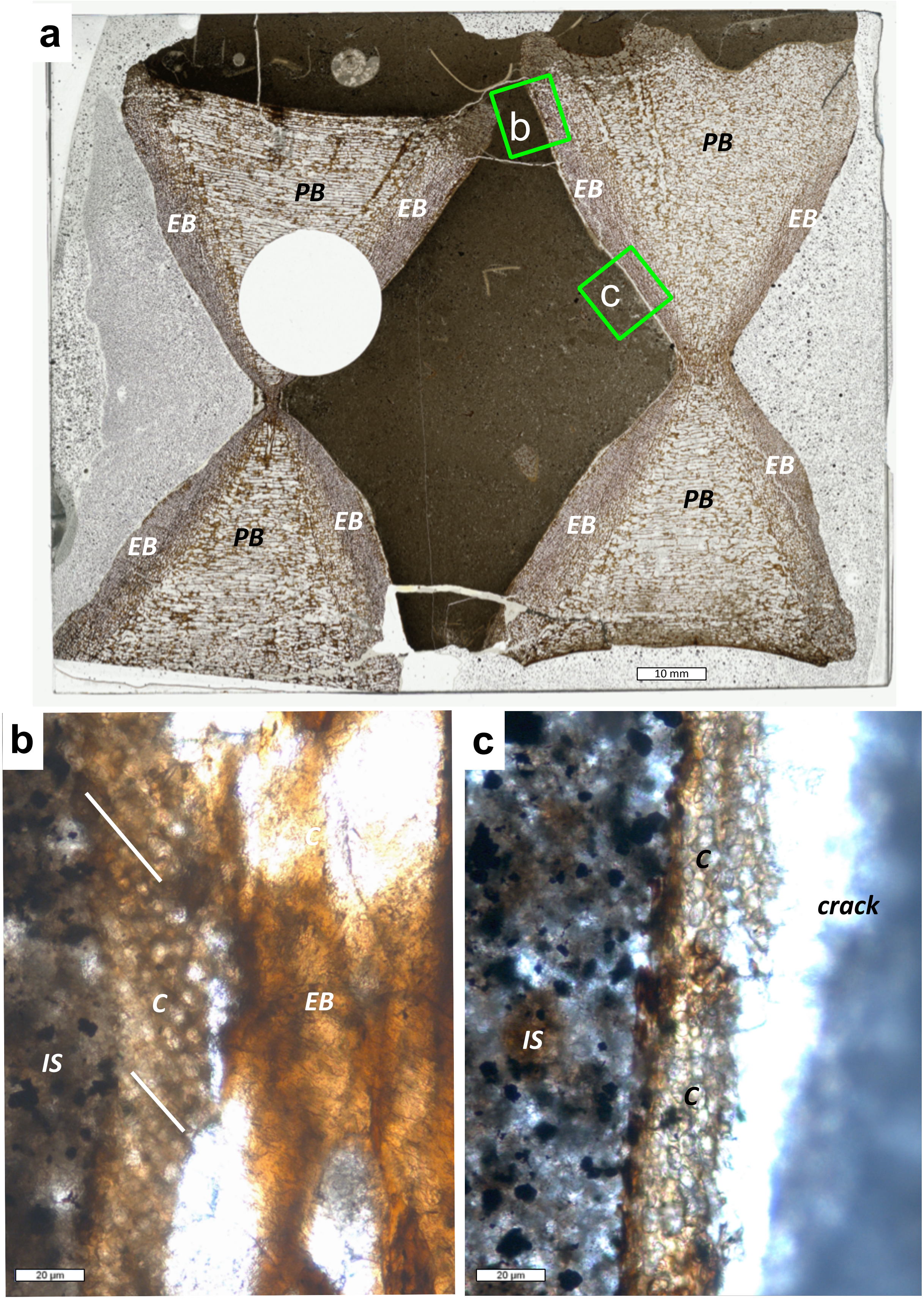

Figure S10. Histology of cf. *Cymbospondylus* IGPB R 660, joint with IVD. a Sagittal section of a segment of vertebral column, neural canal is to the top. Image in plane polarised light. Translucent circle resulted from coring of the bone. **b** Enlargement of peripheral part of articular surface (area b) in cross polarised light. Note the oblique orientation of the chondrocyte files (white lines). **c** Close-up of area c. The cartilage layer is separated from underlying endochondral bone (not visible) by a crack. Note the loose and irregular arrangement of the chondrocyte lacunae. Image in cross-polarised light. C, cartilage; EB, endochondral bone; IS, intervertebral space; PB, periosteal bone tissue.

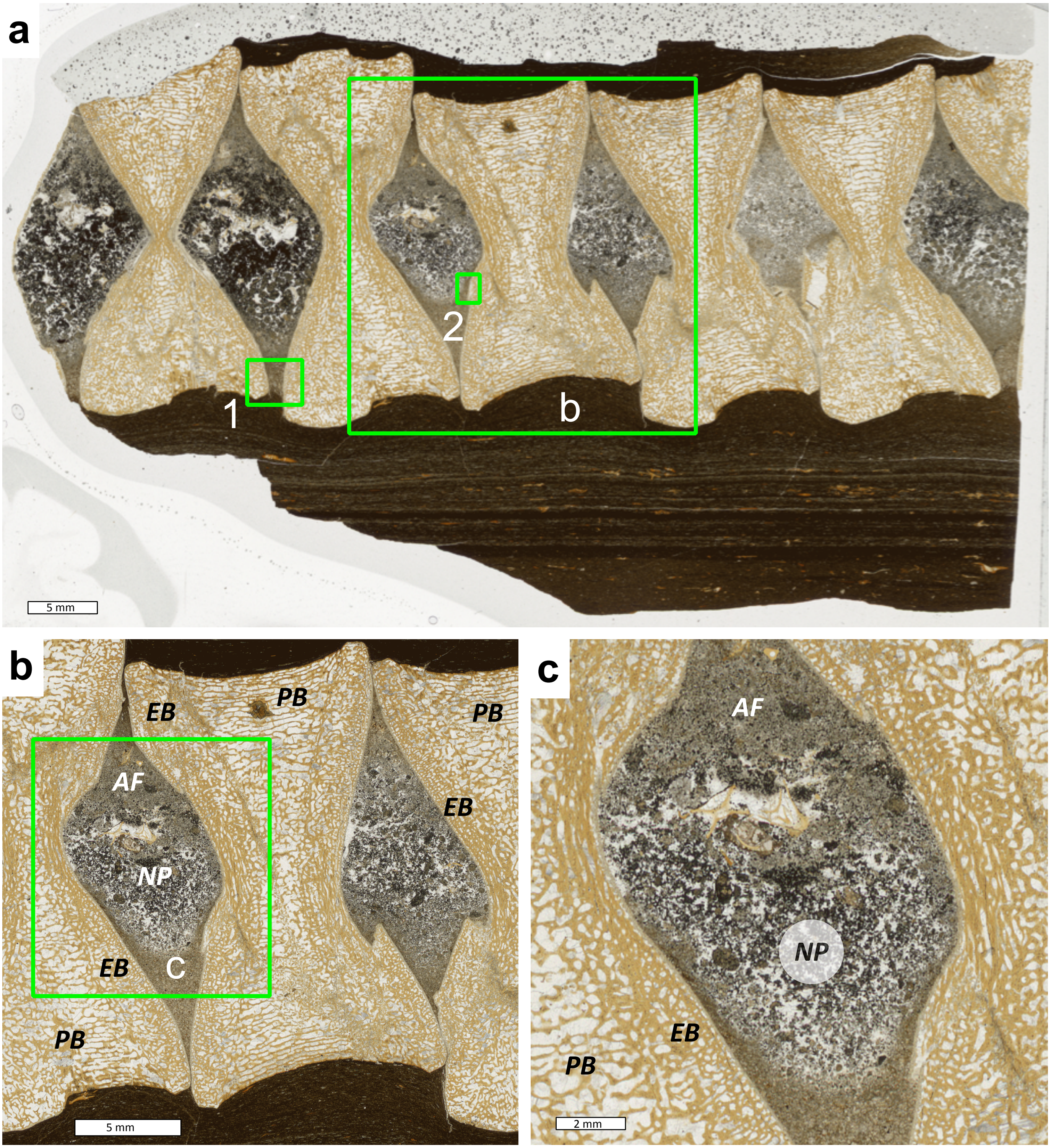

Figure S11. Histology of *Stenopterygius* sp. IGPB R 661, joint with IVD. a Sagittal section of a segment of vertebral column, neural canal is to the top. Image in plane polarised light. Note the laminated sediment below the vertebrae and the granular fill of the intervertebral spaces. **b** Enlargement of three articulating vertebrae. Note the apparent preservation of an AF and NP. **c** Close-up of the intervertebral space. All images are in plane-polarised light. AF, annulus fibrosus; EB, endochondral bone; NP, nucleus pulposus; PB, periosteal bone.

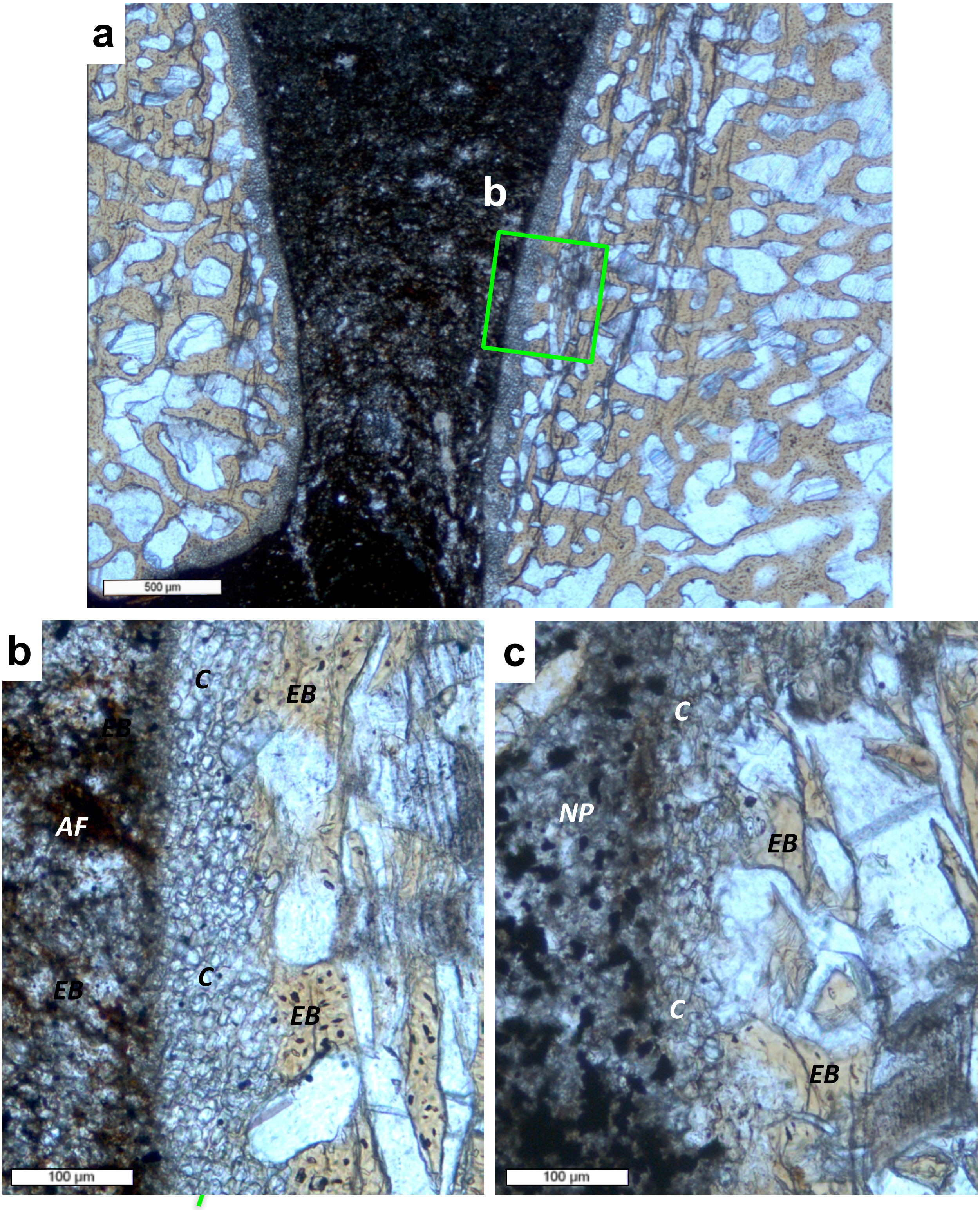

Figure S12. Histology of *Stenopterygius* sp. IGPB R 661 continued. a Close-up of the ventral peripheral part of two articulating vertebrae. See previous figure for location (area 1). **b** Enlargement of peripheral part of articular surface. Note the oblique orientation of the chondrocyte files (green arrows). **c** Close-up of inner articular surface, see previous figure for location (area 2). Note the loose and irregular arrangement of the chondrocyte lacunae. All images in cross-polarised light. AF, annulus fibrosus; C, cartilage; EB, endochondral bone; NP, nucleus pulposus; PB, periosteal bone.

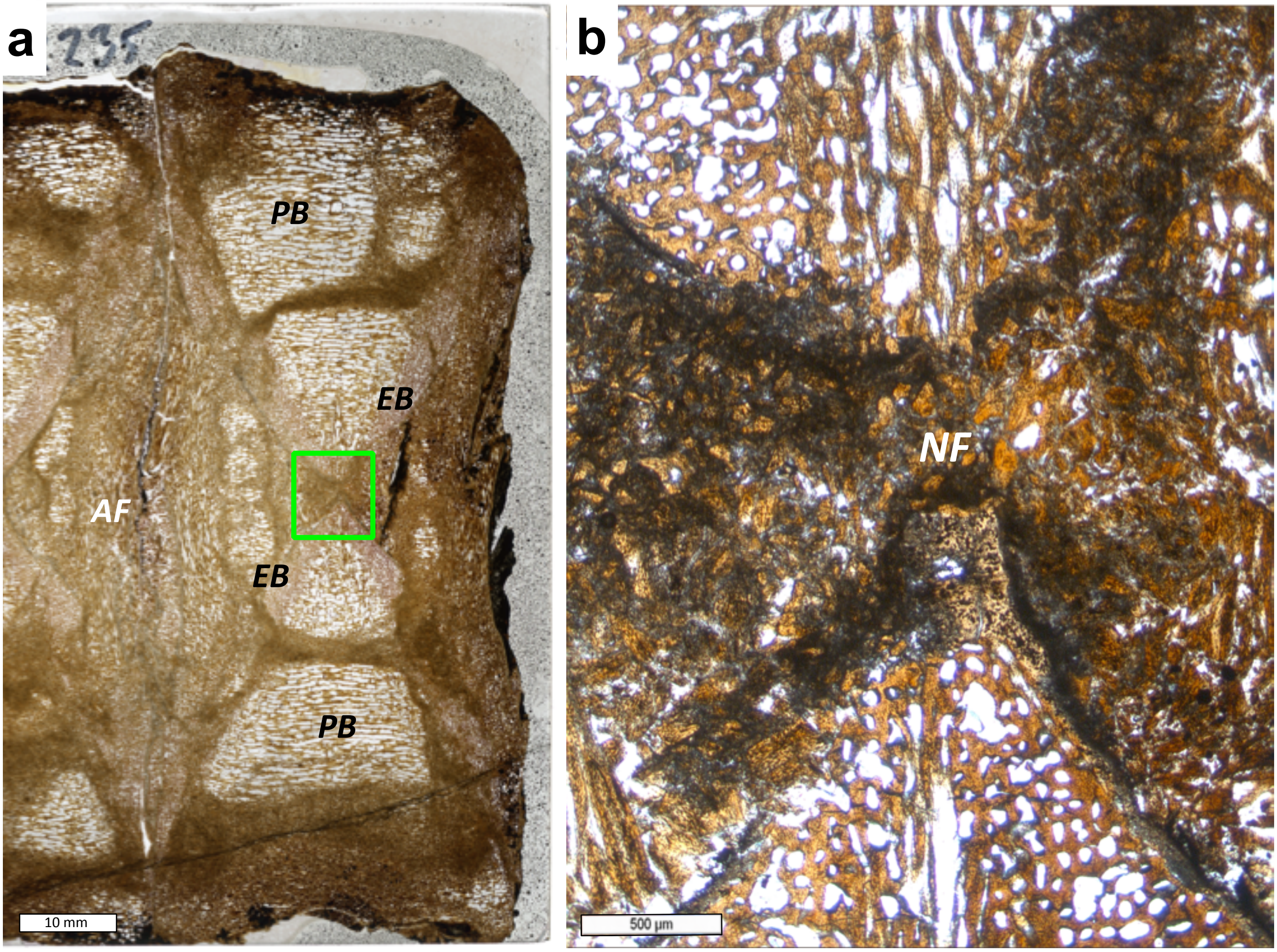

Figure S13. Histology of *Leptopterygius* sp. IGPB R235, joint with IVD and vestigial notochord. a Sagittal section of two articulated vertebral centra, neural canal is to the top. Translucent circle resulted from coring of the bone. **b** Enlargement of middle of the centrum. Note the small notochord foramen. Images in plane polarised light. EB, endochondral bone; NF, notochord foramen; PB, periosteal bone.

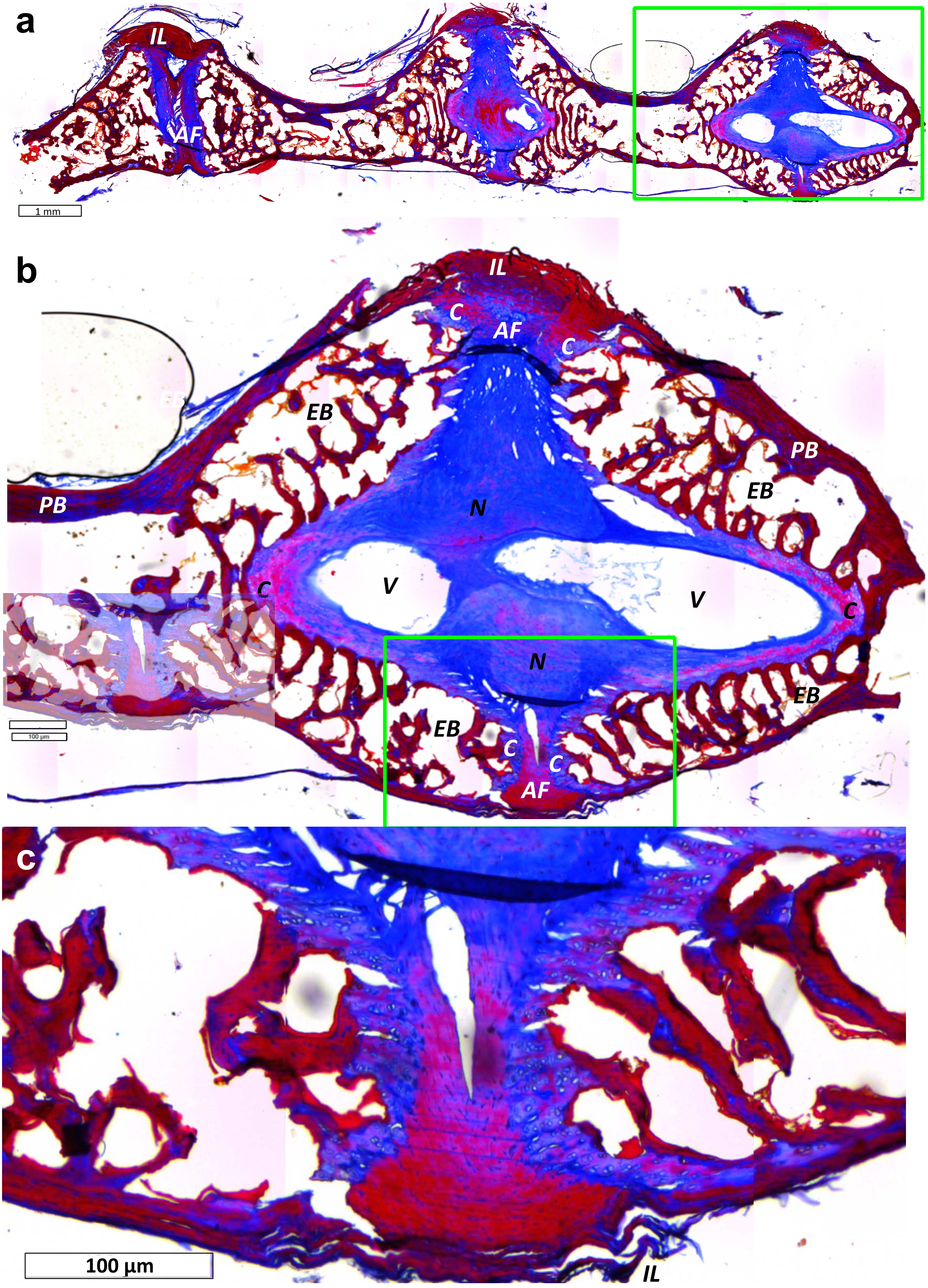
a
IL
AF
1 mm
b
IL
C
AF
C
EB
EB
PB
PB
EB
N
V
V
C
C
N
EB
EB
C
C
AF
100 µm
c
100 µm
IL

Figure S14 Histology of *Sphenodon punctatus* NHMW 8108, joint with persisting notochord. a Oblique sagittal microtome section stained with Azan of three articulated vertebral centra, neural canal is to the top. Red is bone and ligaments, cartilage stains pink and light blue, notochord stains darker blue. Note that the notochord appears discontinuous in this image because of the suboptimal planes of section. **b** Enlargement of the intervertebral joint of the centrum. Note the vesicle in the notochord. **c** Enlargement of the ventral part of the AF. AF, annulus fibrosus; C, cartilage; EB, endochondral bone trabeculae; IL, intervertebral ligament; N, notochord; PB, periosteal bone tissue; V, vesicle.

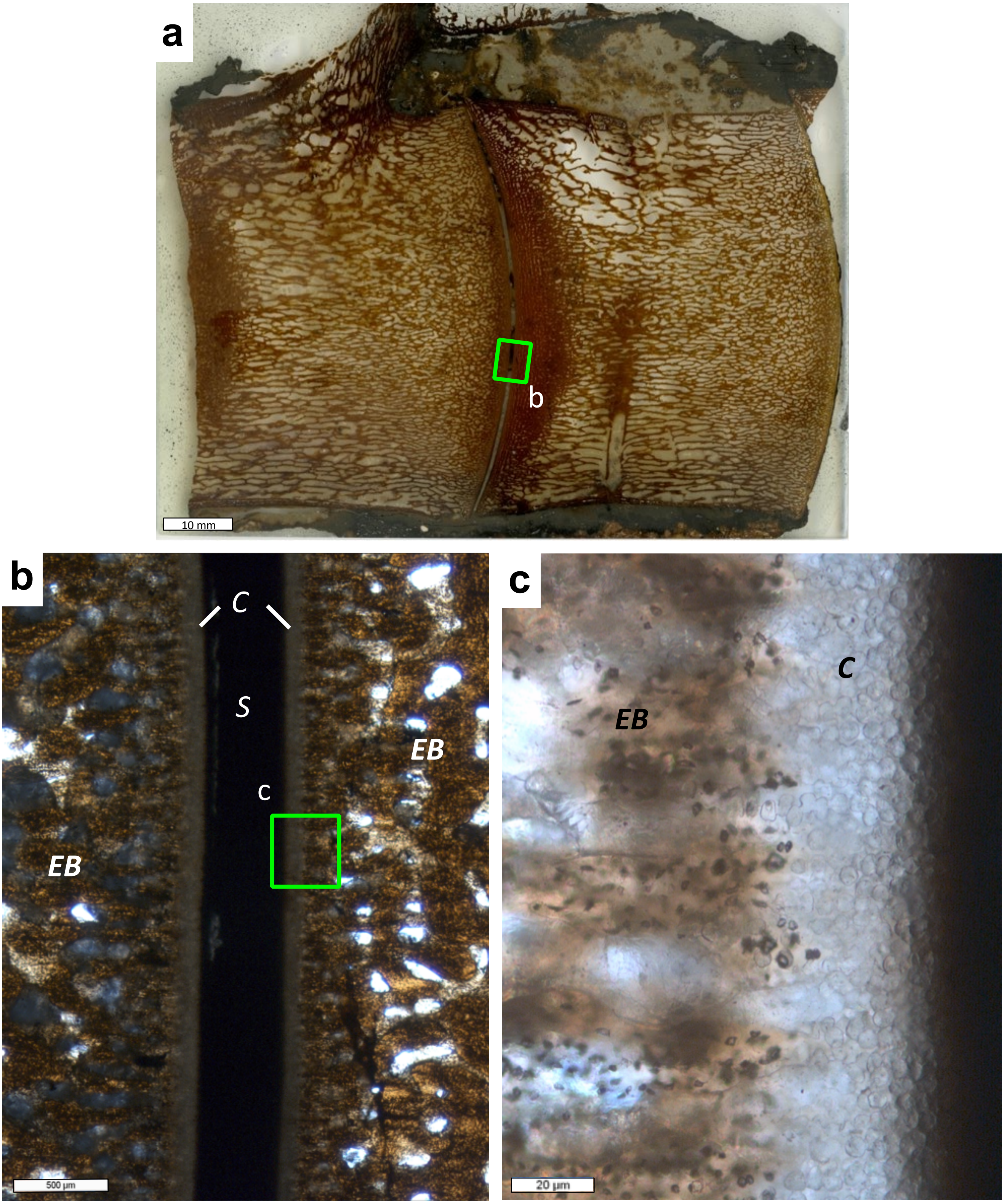

Figure S15. Histology of *Mosasaurus missouriensis* IGPB Goldfuß 1230, synovial ball-and-socket joint. **a** Sagittal section of two articulated vertebra, neural canal is to the top. Image in plane polarised light. **b** Enlargement of peripheral part of articular surface (area b) in cross-polarised light. Note the very narrow joint space and the smooth articular surface. **c** Close-up of area c. Note the well preserved hyaline cartilage with the chondrocyte lacunae are arranged in regular files perpendicular to the surface. Image in cross-polarised light. C, cartilage; EB, endochondral bone trabeculae; S, synovial space.

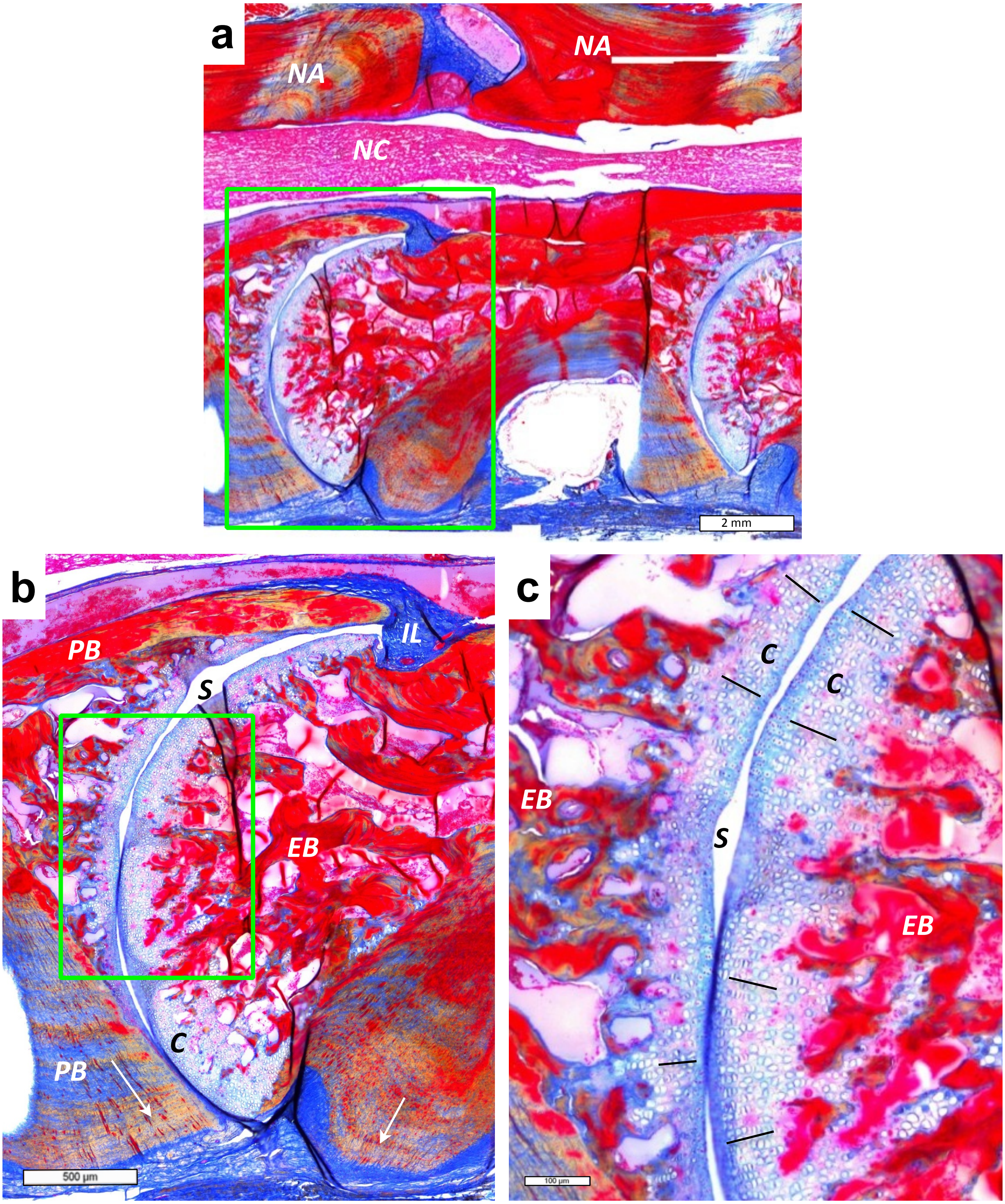

Figure S16. Histology of *Python* sp. IGPB R 662, synovial ball-and-socket joint. a Sagittal microtome section stained with Azan of three articulated vertebral centra, neural canal is to the top. Red is bone and ligaments, cartilage stains pink and light blue, ligament stains darker blue. **b** Enlargement of joint. Note the very narrow joint space, the smooth articular surface, and the Sharpey's fibres (white arrows) in the periosteal bone. **c** Close-up of **b**. Note the hyaline cartilage in which the chondrocyte lacunae are arranged in regular files perpendicular to the surface (black lines). C, cartilage; EB, endochondral bone trabeculae; IL, intervertebral ligament; NA, neural arch; NC, neural canal; PB, periosteal bone; S, synovial joint space.

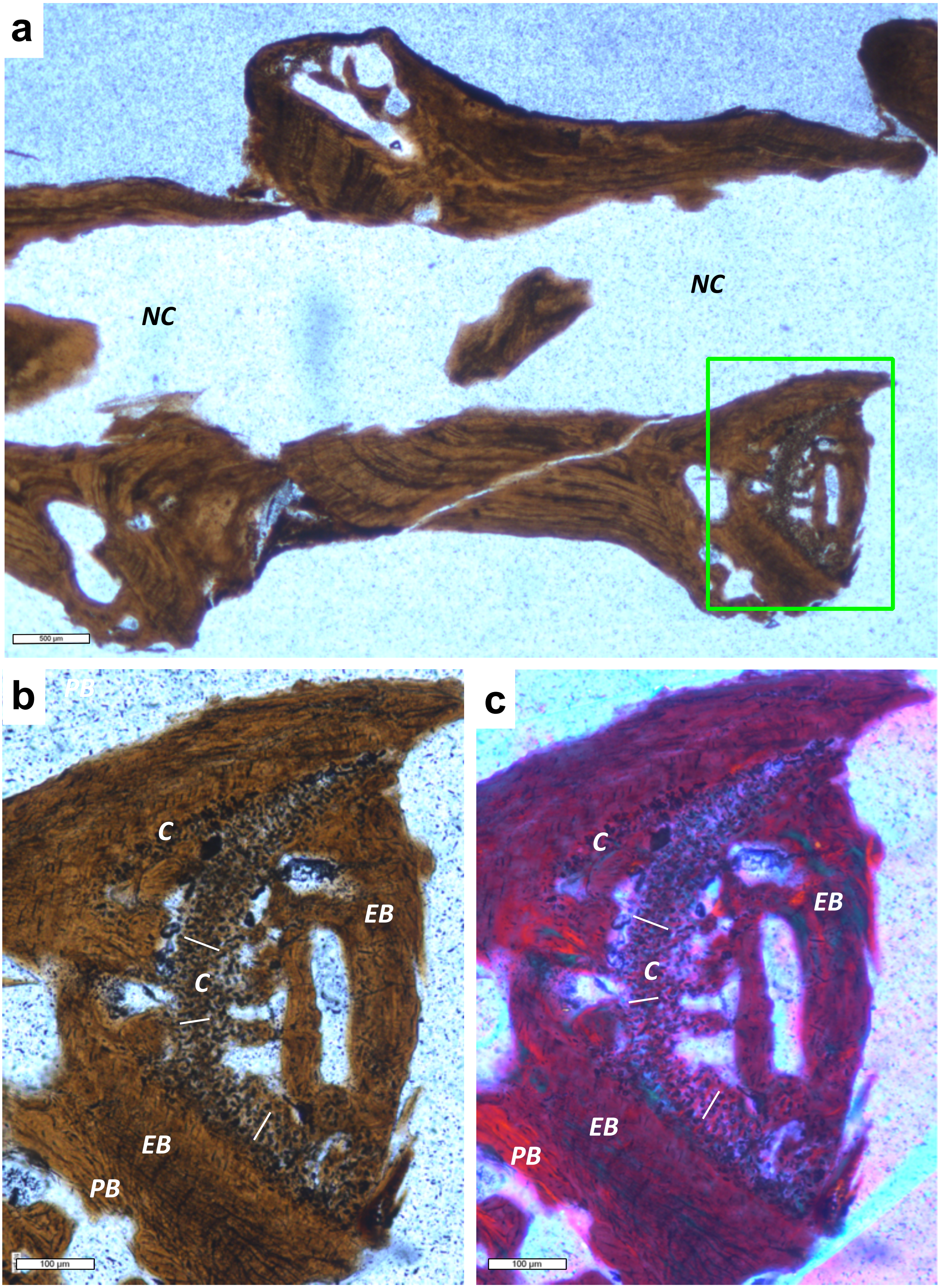

Figure S17. Histology of Ophidia indet. HLMD ME 7624b, synovial ball-and-socket joint. a Sagittal section of three articulated vertebra, very large neural canal is to the top. Image in plane polarised light. **b** Enlargement of right joint (green box) in plane polarised light. The joint space was closed over during fossilisation. Note the well preserved hyaline cartilage with the chondrocyte lacunae arranged in regular files perpendicular to the surface (white lines). **c** Same as previous but in cross-polarised light and lambda filter. C, cartilage; EB, endochondral bone trabeculae; NC, neural canal; PB, periosteal bone.

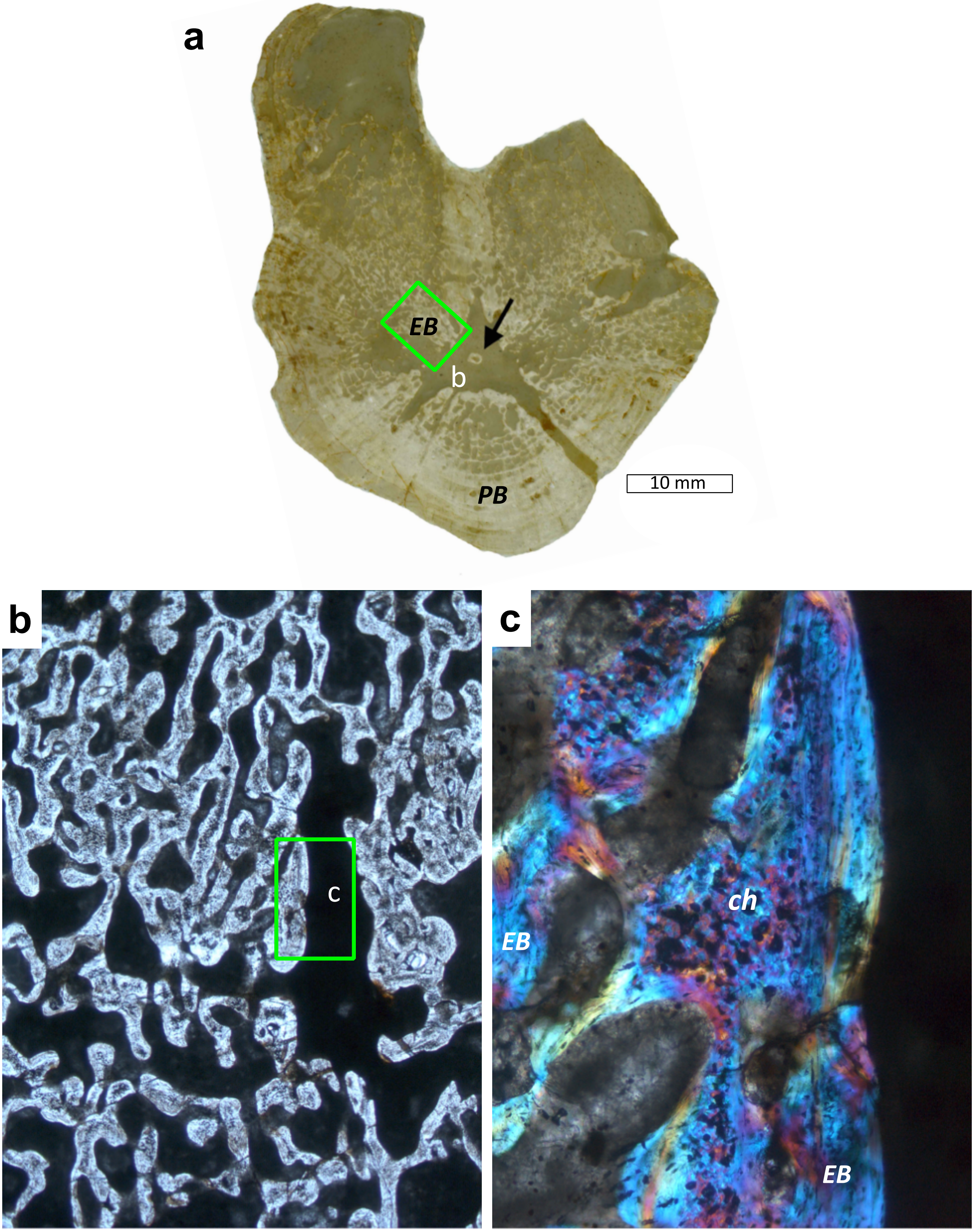

Figure S18. Histology of *Pladocus gigas* IGPB R 86, joint with IVD. a Transverse section of an isolated vertebra, neural canal is to the top. Image in plane polarised light. **b** Enlargement of middle part of articular surface (area b) showing endochondral trabeculae in cross polarised light. **c** Close-up of area c in cross-polarised light and lambda filter. Note the abundant chondrocyte lacunae, some of which are calcified, in the core of the trabeculae. ch, chondrocytes; EB, endochondral bone trabeculae; PB, periosteal bone.

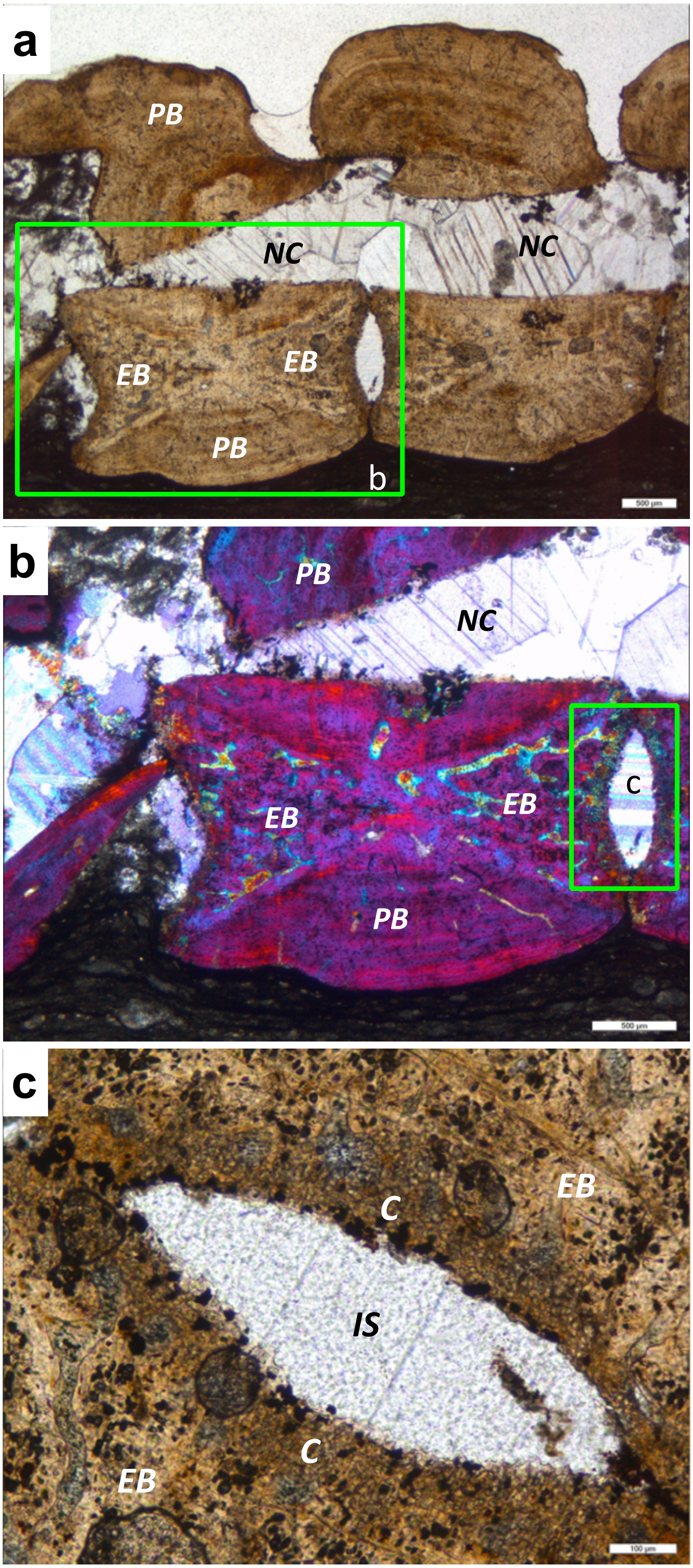

Figure S19. Histology of *Neusticosaurus peyeri* PIMUZ T 3768, joint with IVD. a Sagittal section of four articulated vertebrae, neural canal and spine is to the top. Image in plane polarised light. **b** Enlargement of one vertebral centrum articulating with two others in cross-polarised light and lambda filter. Note the platycoelous shape and the clear separation of the periosteal and endochondral domains. **c** Close-up of **b** showing the joint area of two articulated vertebral centra in plane polarised light. Note the cartilage layer on the articular surface of both vertebrae. C, cartilage; EB, endochondral bone; IS, intervertebral space; NC, neural canal; PB, periosteal bone.

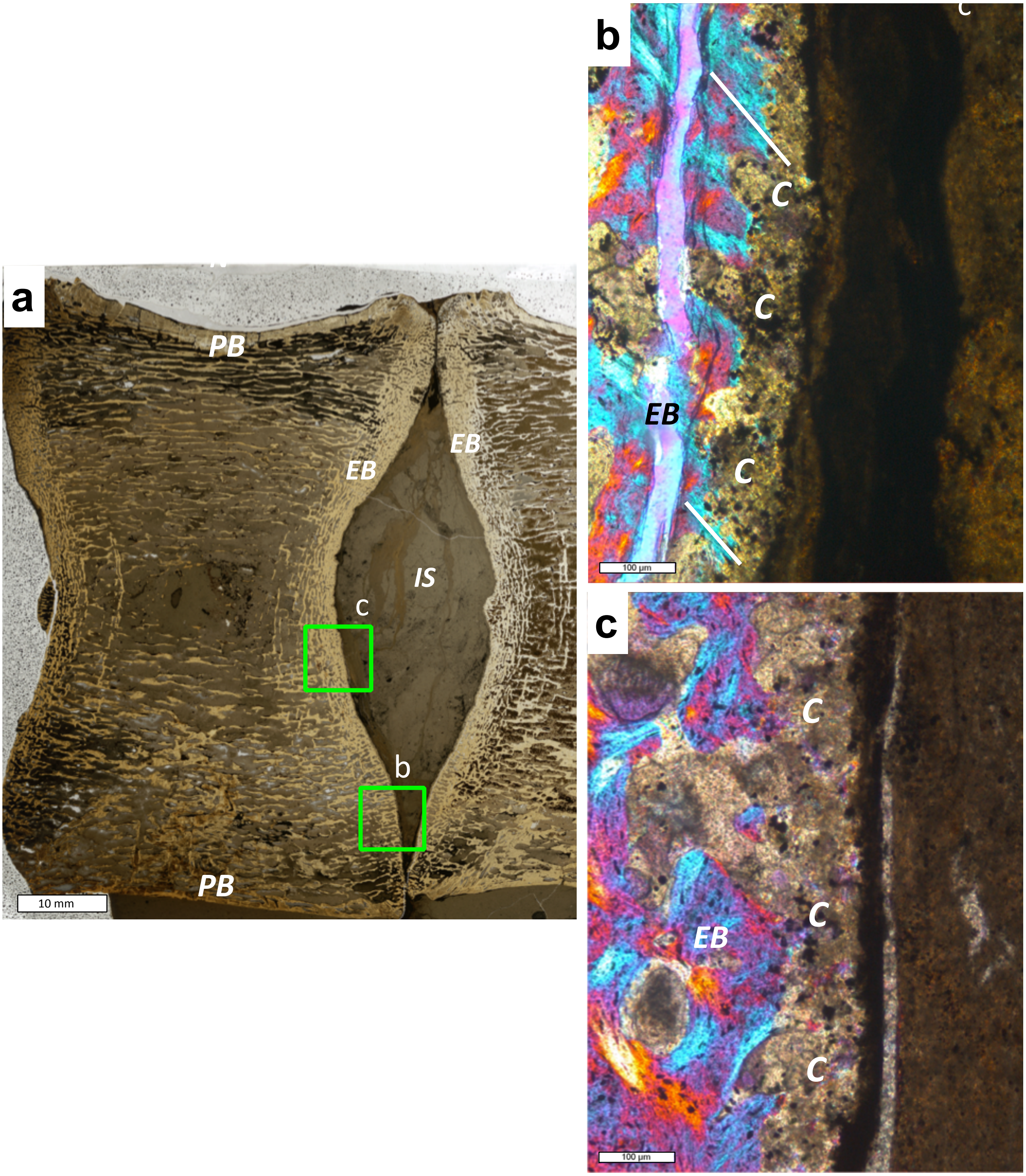

Figure S20. Histology of *Plesiosaurus dolichodeirus* IGPB R 88, joint with IVD. a Sagittal section of a two articulated vertebral centra, neural canal is to the top. Image in plane polarised light. **b** Enlargement of peripheral part of articular surface (area b) in cross polarised light and lambda filter. Note the clear separation of bone and cartilage revealing the peripherally inclined chondrocyte files and the insertion of fibres (white lines) into the bony areas in between. **c** Enlargement of middle part of articular surface (area c). Note the loose and irregular arrangement of the chondrocyte lacunae and the lack of fibre insertions. Image in cross-polarised light. C, cartilage; EB, endochondral bone trabeculae; IS, intervertebral space; PB, periosteal bone.

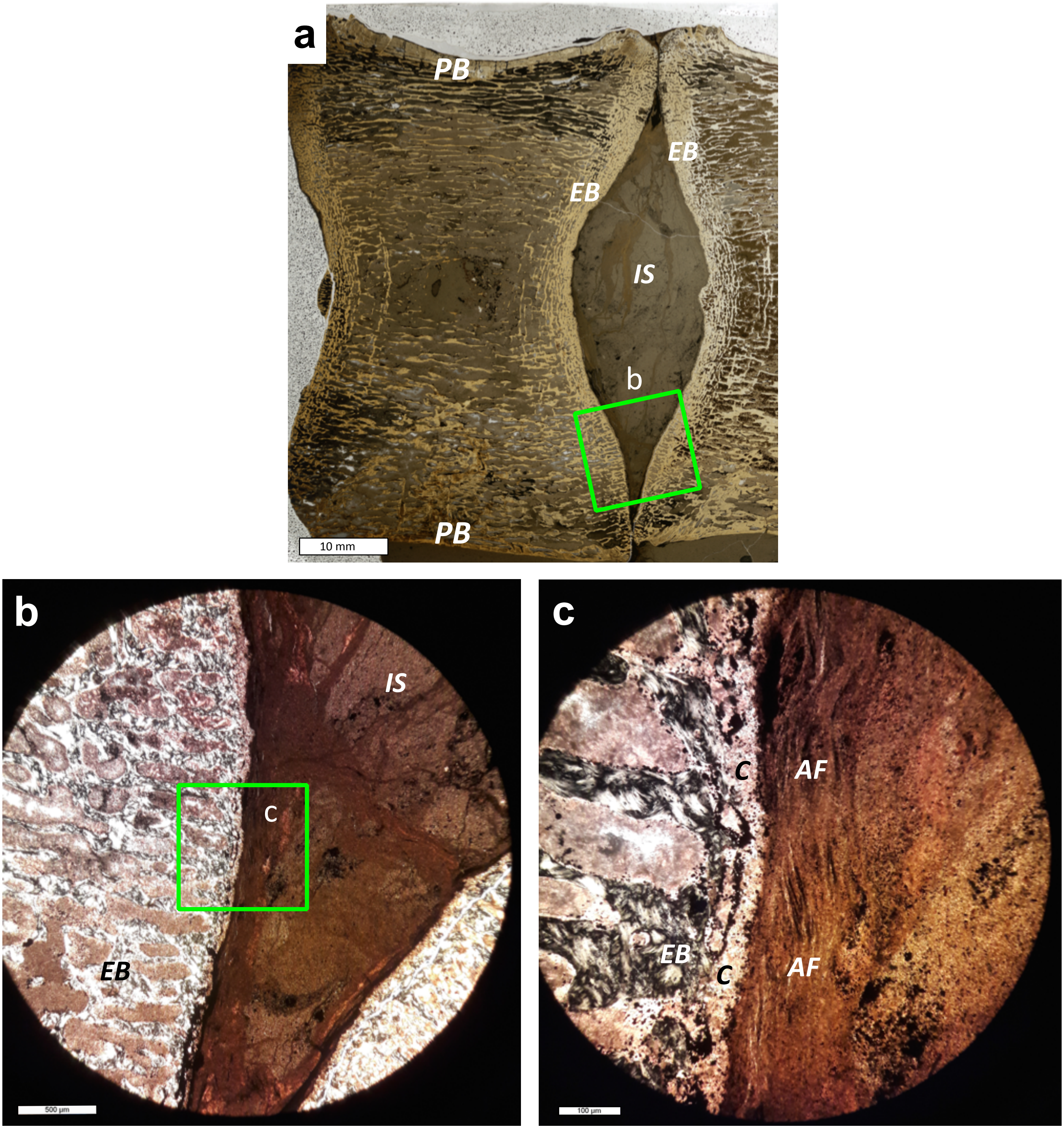

Figure S21. Histology of *Plesiosaurus dolichodeirus* IGPB R 88 continued. a Sagittal section of a two articulated vertebral centra, neural canal is to the top. Image in plane polarised light. **b** Ventral part of the intervertebral space showing probable altered non-mineralized tissues of the IVD. **c** Enlargement of area c. Note the fibrous layer overlying the cartilage on the articular surface. The fibrous layer probably represent the AF of the IVD. Images in **b** and **c** in cross-polarised light. AF, probable annulus fibrosus; C, cartilage; EB, endochondral bone trabeculae; IS, intervertebral space; PB, periosteal bone.

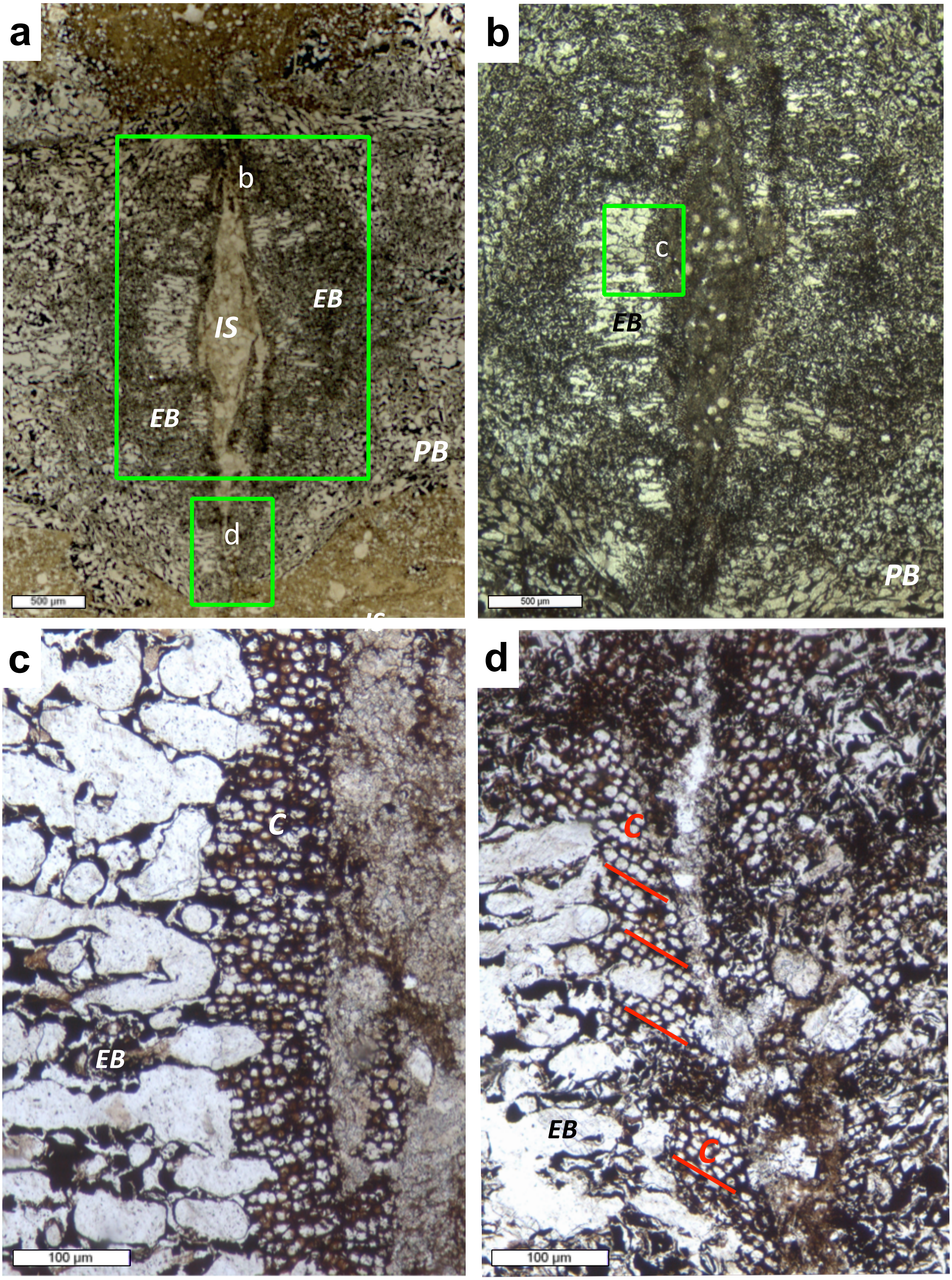

Figure S22. Histology of *Xinpusaurus* HFUT GL 17006, joint with IVD. a Sagittal section of a two articulated vertebral centra, neural canal is to the top. **b** Enlargement of central part of intervertebral joint. Despite the poor preservation of the bone tissue histology, bone and cartilage can be distinguished, revealing the peripherally inclined chondrocyte files and the insertion of fibres into the bony areas in between. **c** Enlargement of area c. Note the partially mineralized files of chondrocyte lacunae oriented perpendicular to the articular surface.These are overlain by unmineralised and less well organised chondrocytes. **d** Enlargement of peripheral part of articular surface (area d). Note the peripherally inclined chondrocyte files (red lines). All images in plane polarised light. C, cartilage; EB, endochondral bone trabeculae; IS, intervertebral space; PB, periosteal bone.

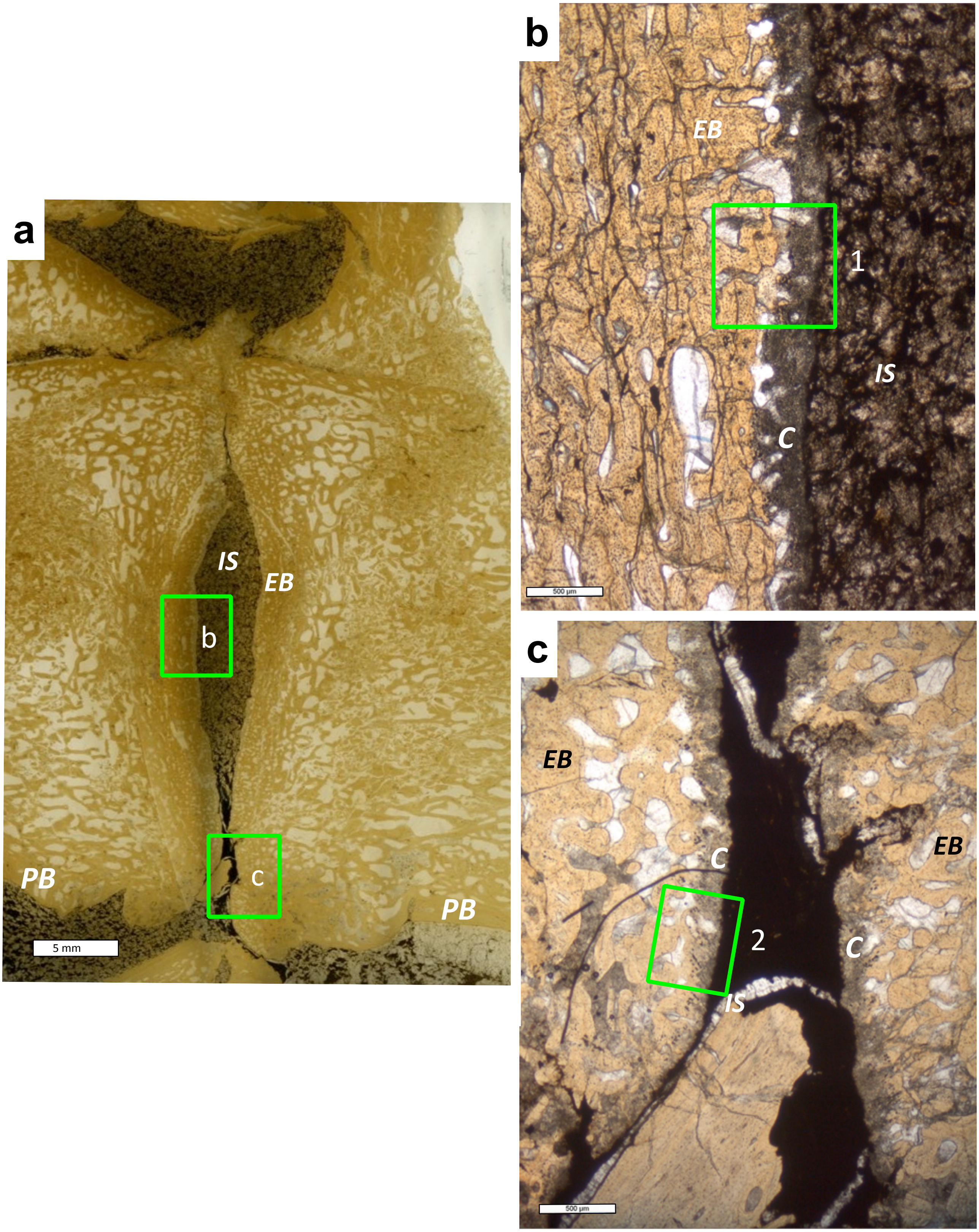

Figure S23. Histology of *Steneosaurus* IGPB R 663, joint with IVD. a Sagittal section of a two articulated vertebral centra, neural canal is to the top. Image in plane polarised light. **b** Enlargement of middle part of articular surface (area c). Note the clear separation of bone and cartilage and the loose and irregular arrangement of the chondrocyte lacunae and the lack of fibre insertions. Image in cross-polarised light. **c** Enlargement of peripheral part of articular surfaces (area b) in cross polarised light. Note the clear separation of bone and cartilage revealing the peripherally inclined chondrocyte files and the insertion of fibres into the bony areas in between. See following figure for close-ups. C, cartilage; EB, endochondral bone; IS, intervertebral space; PB, periosteal bone.

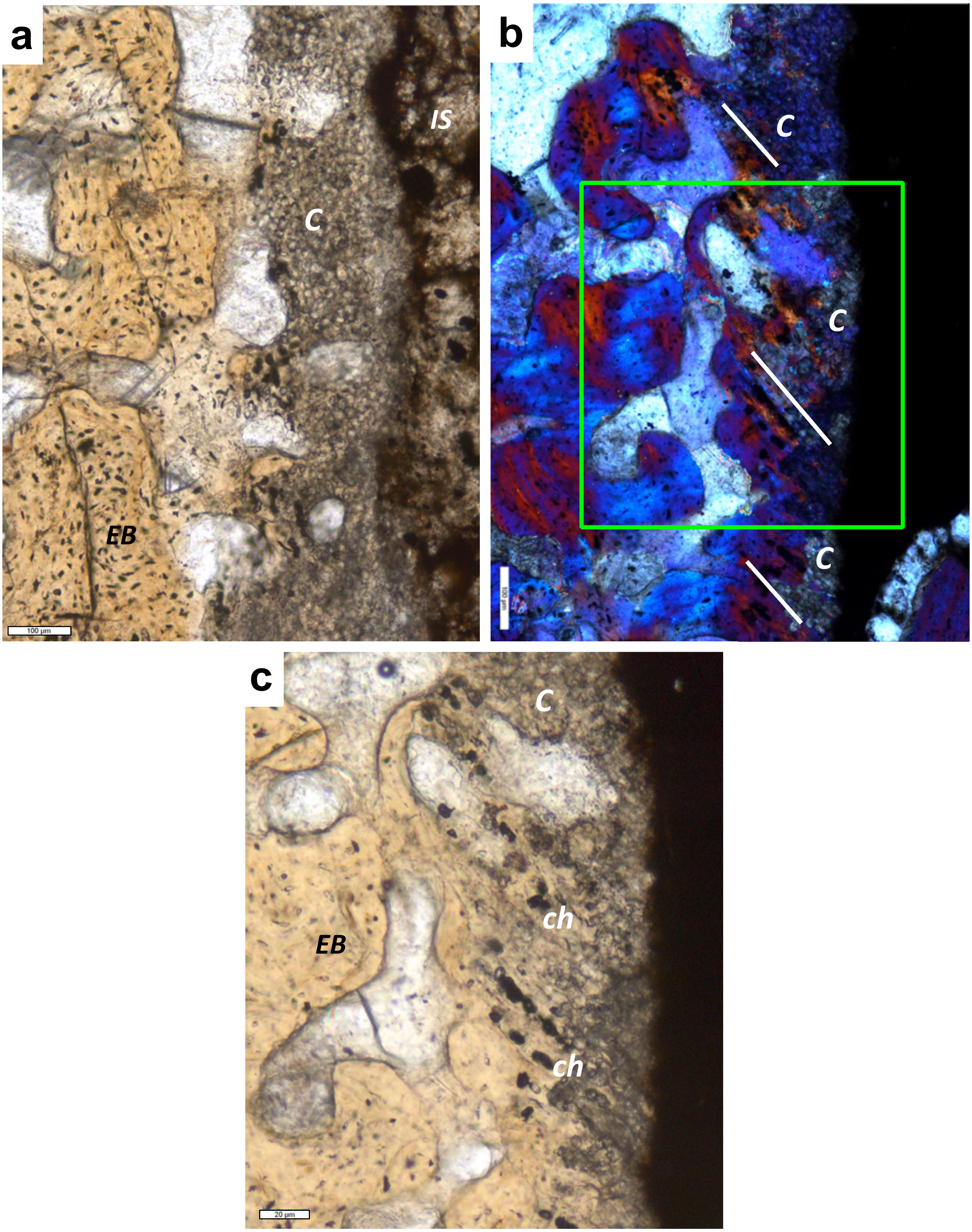

Figure S24. Histology of *Steneosaurus* IGPB R 663 continued. a Close-up of area 1 in previous figure, cross-polarised light. **b** Close-up of area 2 in cross-polarised light and lambda filter. Note the oblique orientation of the chondrocyte files (white lines) flanked by insertion fibres in the endochondral bone. **c** Close-up of **b** in normal light. Note the distinctive chondrocyte lacunae in the endochondral bone. C, cartilage; EB, endochondral bone; IS, intervertebral space; PB, periosteal bone.

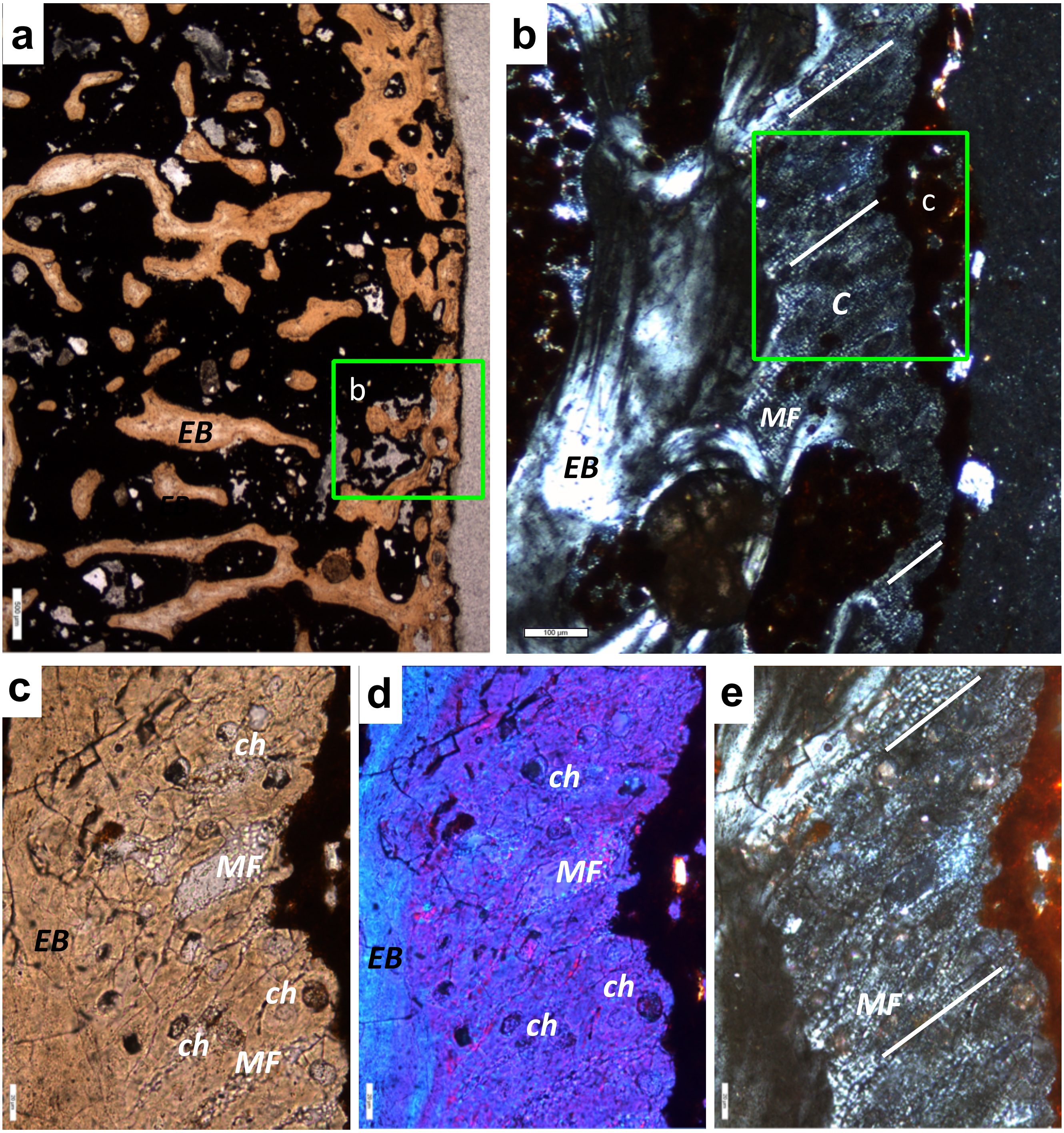

Figure S25. Histology of Hadrosauridae indet UACVP 59650, joint with IVD. a Sagittal section of the peripheral part of the articular surface of an isolated vertebral centrum in plane polarised light. **b** Enlargement in cross-polarised light. Note the outwardly inclined mineralised fibres (white lines) . **c** Close-up showing mineralised fibers and scattered chondrocyte lacunae (large globular bodies). **d** Same in in cross-polarised light and lambda filter. **e** Same in cross-polarised light. C, cartilage; ch, chondrocyte lacunae; EB, endochondral bone; MF, mineralised fibres; PB, periosteal bone.

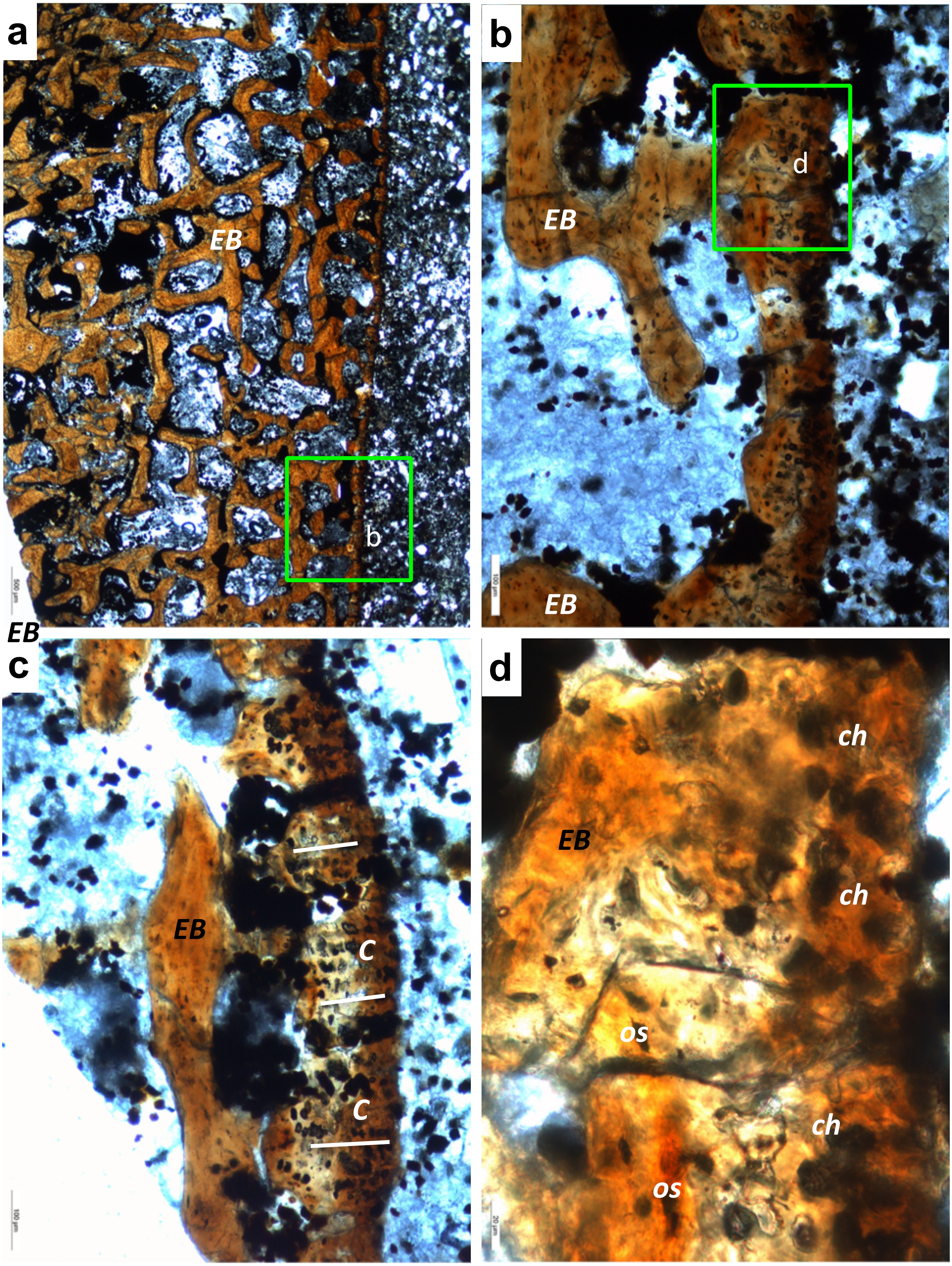

Figure S26. Histology of Ornithomimosauridae indet. UACVP 59651, joint with IVD. a Sagittal section of the articular surface of an isolated vertebral centrum in cross-polarised light. **b** Enlargement showing transition from zone of irregular chondrocyte lacunae to zone with files of chondrocyte lacunae (white lines) oriented perpendicular to the articular surface. **c** Other area of files of chondrocyte lacunae oriented perpendicular to the articular surface. **d** Close-up of irregular chondrocyte lacunae in **b**. All image in cross-polarised light. C, cartilage; ch, chondrocyte lacunae; EB, endochondral bone trabeculae; os, osteocyte lacunae.

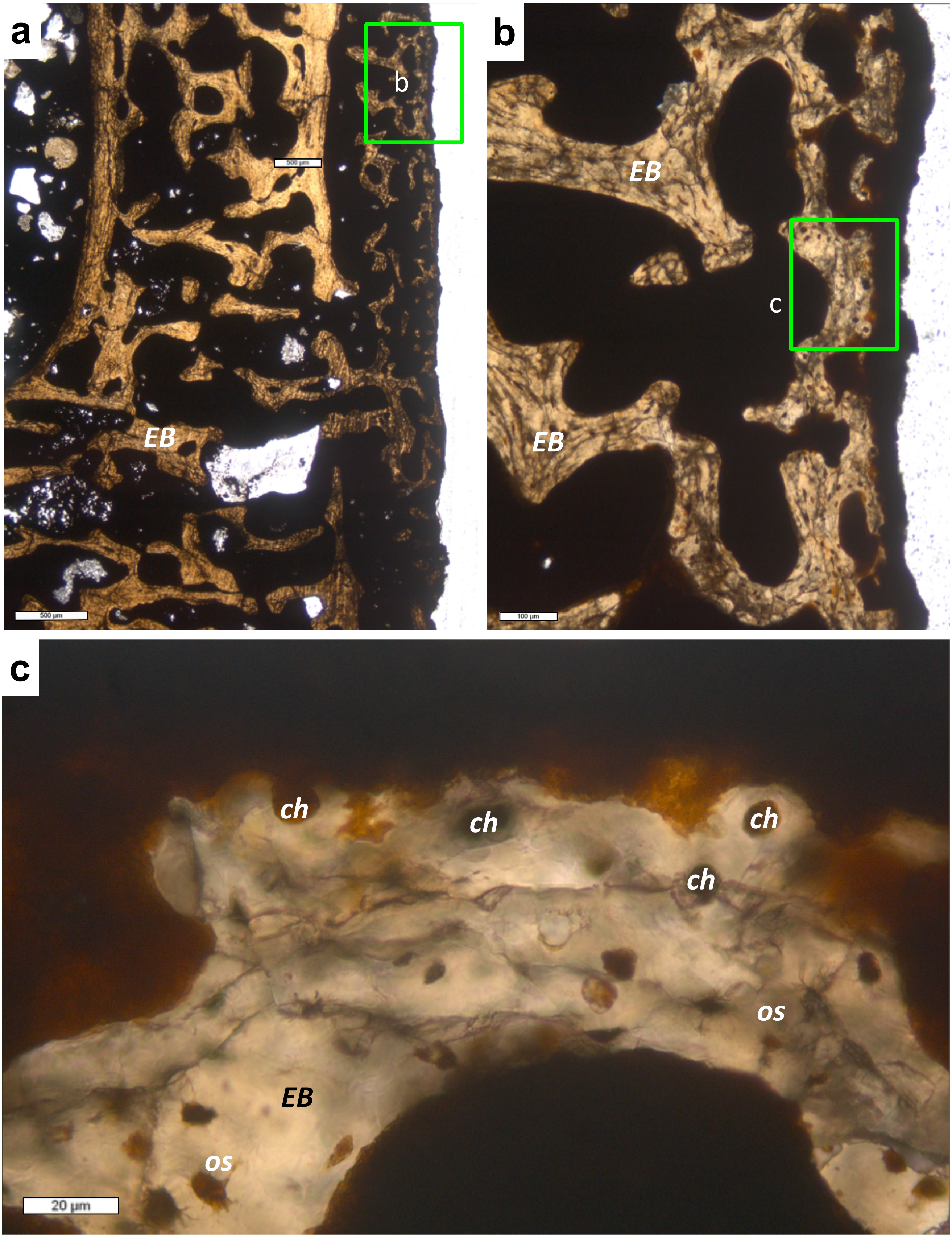

Figure S27. Histology of Dromaeosauridae indet. UACVP 56952, fibrous cartilage joint. a Sagittal section of the articular surface of an isolated vertebral centrum. **b** Enlargement of **a**. Note the apparent lack of any cartilage. **c** Closeup of **b**. Note the scattered chondrocyte lacunae and the bony trabeculae with osteocyte lacunae (bottom left). All images in normal light. ch, chondrocyte lacunae; EB, endochondral bone trabeculae; os, osteocyte lacunae.

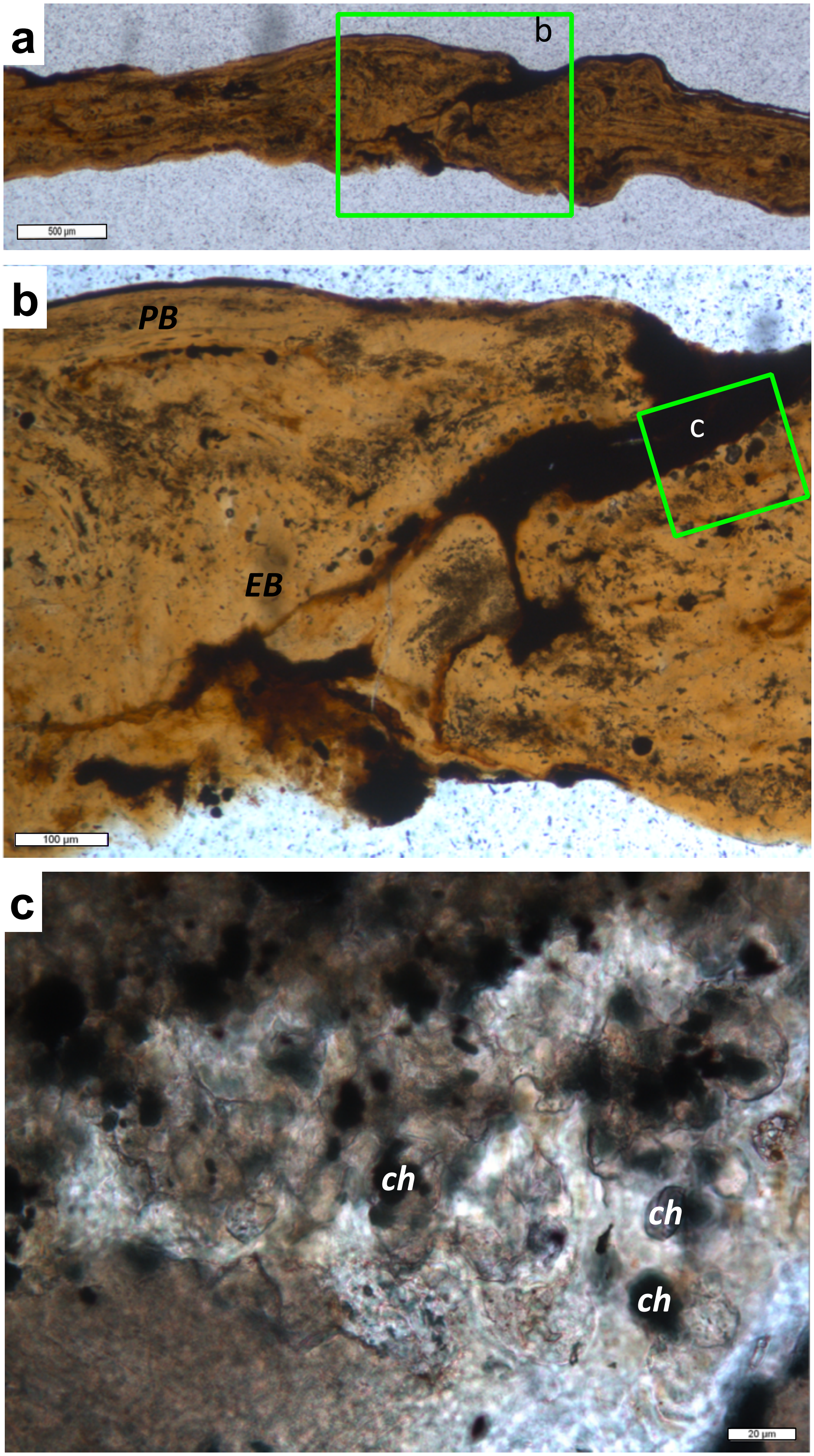

Figure S28. Histology of Aves indet. HLMD ME 13019 from Messel, fibrous cartilage joint. a Sagittal section of two articulated vertebral centra. Image in plane polarised light. **b** Enlargement of joint (green box) in plane polarised light. The joint space appears closed over during fossilisation. **c** Close-up of **b**. Note the well scattered chondrocyte lacunae. Image in cross-polarised light. ch, chondrocyte lacunae; EB, endochondral bone; PB, periosteal bone.

Wintrich et al. Supplementary Figures

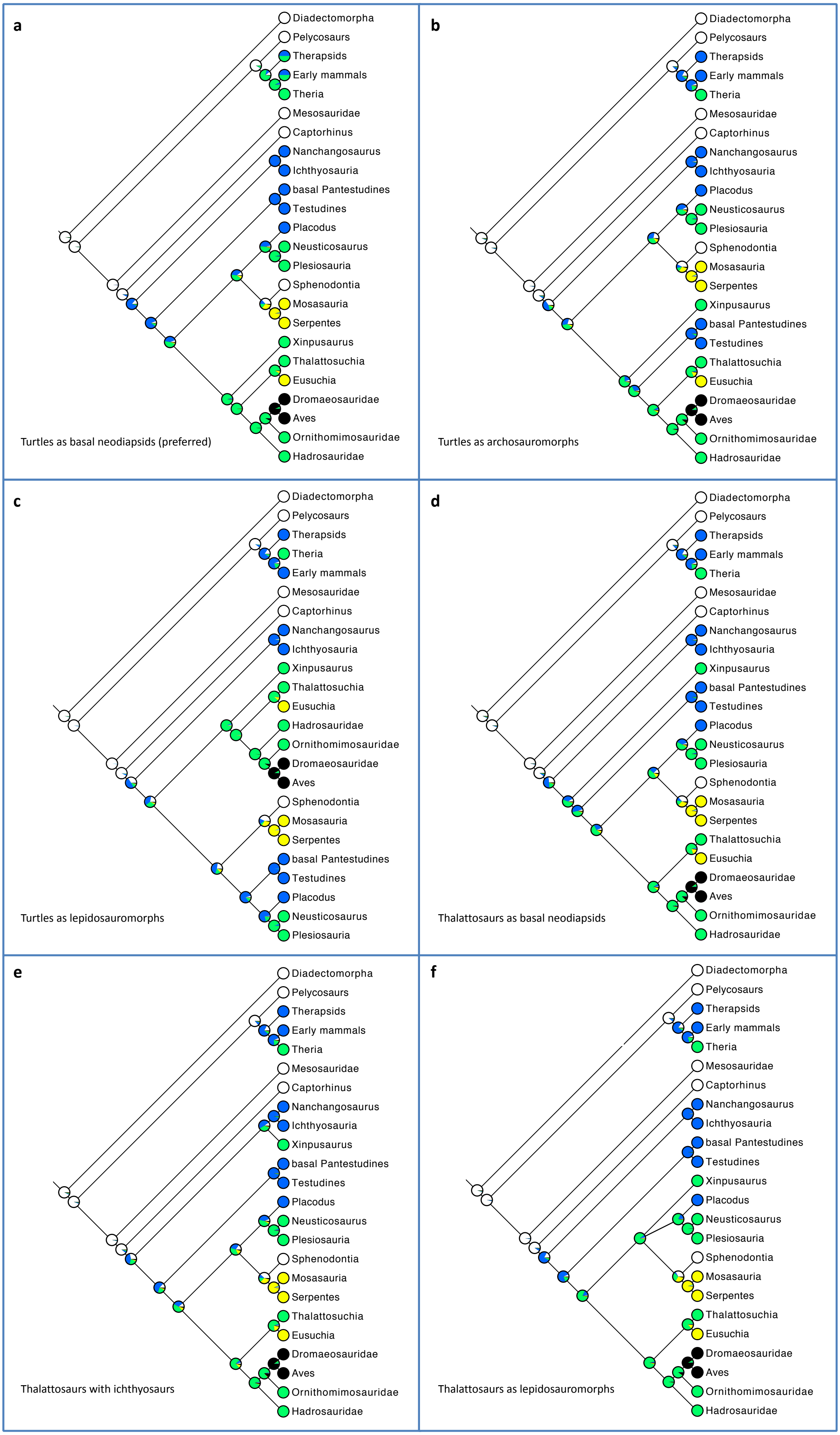

Figure S29. Ancestral state reconstructions of intervertebral articulation in amniotes. Three different hypotheses about turtle relationships and three different hypotheses of thalattosaur relationships were tested (see Methods). The optimality criterium is maximum likelihood. Colour coding of character states: White, persisting notochord; blue, IVD between amphicoelous centra; green, IVD between platycoelous centra; yellow, synovial joint with hyaline cartilage in between procoelous or opisthocoelous centra; black, fibrous cartilage joint. **a**, Turtles are basal neodiapsids. This is the preferred hypothesis, also shown in Fig. 3. **b**, Turtles are archosauromorphs. **c**, Turtles are lepidosauromorphs and form a clade with sauropterygians. **d**, Thalattosaurs are basal neodiapsids. **e**, Thalattosaurs form a clade with ichthyosaurs. **f**, Thalattosaurs are lepidosauromorphs. Note that the different phylogenetic hypotheses do not fundamentally influence the result that the IVD evolved convergently at least twice.
