# Supplementary material for: Palaeontological evidence reveals convergent evolution of intervertebral joint types in amniotes
Source: Sci Rep. 2020 Aug 24;10:14106. doi: 10.1038/s41598-020-70751-2 (PMC7445751; doi:10.1038/s41598-020-70751-2)
Supplement: Supplementary file 2 [file 41598_2020_70751_MOESM2_ESM.docx]

Supplementary Information

for

**Palaeontological evidence reveals convergent evolution of intervertebral joint types in amniotes**

Tanja Wintrich^1,2*^, Martin Scaal^3^, Christine Böhmer^4^, Rico Schellhorn^1^, Ilja Kogan^5^, Aaron van der Reest^6^, P. Martin Sander^1,7^

^1^ *Section Paleontology, Institute of Geosciences, University of Bonn, Nussallee 8, 53115 Bonn, Germany*

^2^ *Institute of Anatomy, University of Bonn, Nussallee 10, 53115 Bonn, Germany*

^3^ *Institute of Anatomy II, University of Cologne, Joseph-Stelzmann-Str. 9, 50937 Cologne, Germany*

^4^ *UMR 7179 CNRS, Muséum national d’Histoire naturelle, Département Adaptations du Vivant, case postale 55, 57 rue Cuvier, F-75231 Paris cedex 05, France*

^5^ *Department of Palaeontology and Stratigraphy, Geological Institute, TU Bergakademie Freiberg, Bernhard-von-Cotta-Str. 2, 09596 Freiberg, Germany* and *Institute of Geology and Petroleum Technologies, Kazan Federal University, Kremlyovskaya Str. 4/5, 420008 Kazan, Russia*

^6^ *Department of Biological Sciences, University of Alberta, Edmonton, Alberta, Canada, T6G 2E9, Canada*

^7^*Dinosaur Institute, Natural History Museum Los Angeles County, 900 Exposition Boulevard, Los Angeles, CA 90007, USA*

* Address for correspondence (Tel: +49 (0) 228 60055; E-mail: [tanja.wintrich@uni-bonn.de](mailto:tanja.wintrich@uni-bonn.de)

Supplementary Text

1. Background information on amniote intervertebral joints

1.1 Disparity of vertebral centrum shape and types of connecting joints

The vertebral column is one of the synapomorphies of vertebrates. During the evolution of the vertebral column from basal vertebrates to amniotes, the structure and morphology changed in different directions and under different selection pressures in different environments. In amniotes, we traditionally distinguish four principal designs of the vertebral bodies or centra (*1*). First, the amphicoelous notochordal vertebral centrum which can be seen, for example, in basal synapsids such as the iconic sail-backed *Dimetrodon*. This vertebral centrum has an hourglass cross section, with a funnel-shaped indentation at the cranial and caudal ends which are connected by a small foramen or canal for the notochord. This condition with the continuous notochord is seen in *Sphenodon* and some geckoes (see below). Second, the non-notochordal amphicoelous centrum in which bone replaced the notochord in the middle of the centrum, such as in ichthyosaurs (*23*). This type of centrum is extinct. Third, the platycoelous vertebral centrum which has a flat surface at the cranial and caudal ends. This structure is seen in mammals and in extinct reptiles, e.g., in most non-avian dinosaurs and in most sauropterygians. In mammals, platycoelous vertebrae are connected by an intervertebral disc (IVD). The fourth vertebral body shape includes procoelous and opisthocoelous. This shape resembles a ball and socket shape, with a bulging surface in the one vertebral centrum and a matching excavation in the following vertebral centrum. In procoelous vertebrae, the socket is anterior and the ball is posterior, whereas in opisthocoelous vertebrae, the ball is anterior and the socket is posterior. However, in both cases they are part of a synovial ball-and-socket joint. An intervertebral ball-and-socket joint is seen in extant reptiles such as most lizards, all snakes, all crocodiles, and the neck vertebrae of turtles. Birds also have ball-and-socket joints that articulate via fibrocartilage. The shape of the vertebral centrum and the nature of the joint is important for the extent and the mode of flexibility of the vertebral column. It has long been recognised that ball-and-socket joints evolved convergently in the different reptile groups, but the IVD has been considered to be unique to mammals.

Joint movement commonly involves coupling of motion around different axes, and the nature and extent of individual joint motions are determined by the joint structure and, specifically, by the shape and direction of the joint surfaces (*11*). In the vertebral column, the range of joint motion, the stability of the joint, and the ultimate functional strength of the joint depends on the geometry of the intervertebral joint surfaces and the surrounding ligamentous capsule constraints (*11*).

1.2 Centrum morphology and development in non-tetrapods

Fishes exhibit a wide variety of vertebral designs. The notochord persists throughout life in cyclostomes, ostracoderms, placoderms, acanthodians, most chondrichthyans and several osteichthyans. While there is no evidence for centra formation in jawless vertebrates and acanthodians, ossified vertebral centra occur in a few placoderms (*48* and references therein), several fossil sarcopterygians, numerous fossils and most extant actinopterygians. Within chondrichthyans, holocephalians exhibit a persistent notochord with cartilaginous arcualia and no centra, whereas in most neoselachians, cartilage cells from the arcualia invade the fibrous sheath of the notochord, giving rise to cartilaginous chordacentra that expand to form hourglass-shaped vertebrae. Accurate age estimates are often possible due to calcified growth zones that mark annual growth stages, at least unless the growth lines converge and become difficult to discern in very old individuals (*e.g., 49*). The notochord becomes constricted and sometimes incised intravertebrally, but occupies the large intervertebral space between the amphicoelous centra (*50*).

In contrast to extant coelacanth and lungfishes that do not possess vertebral centra, amphicoelous or slightly opisthocoelous centra with a small perforation for the notochord were present in some Palaeozoic dipnoans (*48*). Among extinct sarcopterygian groups, vertebral centra are known in certain porolepiforms, osteolepiforms and elpistostegalians. Despite the diversity of vertebral morphologies within these groups, the general patterns are the most widespread aspidospondylous (arcocentral) vertebrae consisting of paired basidorsals (neural arches), paired interdorsals (pleurocentra) and paired ventral arches (intercentra), and holospondylous (ring-like) vertebrae known in *Megalichthys*, *Ectosteorhachis* and a few other genera (*48*, *51*). Centrum morphology can vary along the body (*48*). In any case, the notochord retains its general shape and stabilising function in most non-tetrapod sarcopterygians.

In fossils of early actinopterygians, vertebral structures are often covered by scales. Most non-neopterygians exhibit a notochord with cartilaginous or ossified arcualia, and there are only few taxa with chorda- or arcocentral vertebrae in the shape of half-rings or rings. Notable exceptions are the enigmatic Carboniferous fish *Tarrasius*, which shows amphicoelous vertebrae in a tetrapod-like pattern of axial regionalisation (*52*), and polypteriforms that possess hourglass-shaped centra with an ontogenetically increasing notochord constriction in the middle (*53*).

A variety of vertebral morphologies is also observed in neopterygians. Pycnodonts, for instance, had a persistent notochord with expanded arcualia but no centra. Vertebrae of many fossil halecomorphs are of arcocentral type, i.e. centrum ossification starts from the dorsal and ventral arcualia. In the recent bowfin *Amia calva*, the notochord is completely excluded from the centra and reduced to intervertebral spaces, leading Bartsch (*53*) to use the term *discus intervertebralis* for this structure. Most fossil ginglymodians do not have ossified centra, which otherwise appear as thick perichordal cylinders with no significant notochord constriction (chordacentra?) in macrosemiids; as expanded arcocentra forming half-rings in some lepidotids; or, most uniquely, as solid opisthocoelous vertebrae in gars, and their fossil relatives (*54*). In gars, the notochord is excluded from the intervertebral joint, but it is retained in an intravertebral space (*53*). Finally, all teleosts below the phylogenetic level of *Leptolepis coryphaenoides* have vertebral centra of arcocentral or ring-like chondracentral type, while more advanced teleosts have amphicoelous centra of autocentral type, with or without chordacentra, strongly constricting or incising the notochord (*55*). The intervertebral space is filled by notochord-derived fibrocartilage (*56*).

1.3 Development of the IVD

During embryonic development, the NP is formed at an early ontogenetic stage and is derived from notochord cells, whereas the fibrocartilaginous AF is derived from sclerotomes (*3, 24*). Sclerotomes are the ventral compartments of the somites, the primary mesodermal segments of the vertebrate embryo. The sclerotomes are formed by mesenchymal cells which are proliferative and which migrate as expansive cell populations to their destination in the embryo. Within the sclerotome, at least four subcompartments can be distinguished according to their location in the somite: ventral, central, lateral, and dorsal sclerotome. The ventral sclerotome gives rise to the vertebral centrum and the fibrocartilaginous AF (*10*, *24*), the central sclerotome gives rise to the pedicles of the vertebral arches and the heads of the ribs, whereas the dorsal sclerotome forms the lamina of the neural arch and the lateral sclerotome forms the more distal parts of the ribs (*10, 24, 60*).

During sclerotome development, the process of resegmentation takes place, in which the primary segmentation pattern of the somites is replaced by the secondary segmentation pattern of the vertebrae, which are offset by one segment half. The border between prospective vertebrae, and thus the location of the prospective IVD, is visible already in the intact sclerotome as a fine cleft, which is known as von Ebner’s fissure. The AF of the IVD forms from the sclerotomal cells close to von Ebner’s fissure, which are derived from the somitocoel of the early somites which has been shown to give rise to vertebral joints and is therefore called arthrotome (*58, 59*). Furthermore, during development, the NP is derived from the notochord, a rod-shape embryonic structure in amniotes (*10, 60*).

Mammals differ from most other amniotes in the fact that their long bones have secondary centers of ossification, resulting in bony epiphyses that fuse to the metaphysis upon skeletal maturity. Some mammals also have such secondary centers of ossification in the vertebral centra. In more recent publications dealing with ossification timing in mammals, the pattern is observed that neural arches start to ossify earlier than vertebral centra (*61, 62*), but no statement is given about the presence of epiphyses in vertebrae. There are only few publications describing the presence of bony vertebral centrum epiphyses. Some publications depict rugose vertebral centra surfaces pointing to the presence of bony epiphyses, for example in the kangaroo or the dolphin (*63,* Supplementary Figs 5b and 11b).

1.4 Mammalian intervertebral joints and the bony epiphyses

Mammalian dorsal vertebral centra have secondary centers of ossification, also known as bony epiphyses or just epiphyses that are part of the endochondral domain of the centrum. The soft tissues of the IVD proper connect to theses epiphyses, whereas the connective tissue of the joint capsule inserts in the periosteal domain surrounding the epiphyses. In the albino rat, all vertebral centra have epiphyses at both ends, except atlas and axis, and the last fourteen caudal vertebrae (*64*). Dawson (*65*) draws a slightly different picture with bony epiphyses on both ends only in posterior lumbar vertebrae, and all of the caudal vertebrae. Cervical and anterior thoracic vertebrae lack bony epiphyses, while posterior thoracic vertebrae and anterior lumbar vertebrae possess epiphyses only on the posterior ends of the vertebral centra (*65*). Dawson (*65*) furthermore suggests "that Strong (1925) in his account must have mistaken calcified cartilage for bone". The clarification of the discrepancy between these two authors awaits further investigation. Bony epiphyses are also present for instance in the centra of the European mole (*Talpa europaea*, IGPB M7003), the European water vole (*Arvicola terrestris*, IGPB M1047), and the European hedgehog (*Erinaceus europaeus*, IGPB M7611).

In the reindeer (*Rangifer tarandus*, IGPB M47), the vertebral column shows bony epiphyses at the centra from the posterior end of the axis to the anterior end of the sacrum. The cervical and the lumbar vertebral epiphyses close the sutures with the centra earlier than the thoracal vertebrae. The cervical centra are opisthocoelous, while all other centra are platycoelous. As depicted by Danowitz and Solounias (*66,* fig. 4), extant giraffes (*Giraffa camelopardalis* and *Okapia johnstoni*) also show opisthocoelous cervicals and (at least) posterior epiphyses. Opisthocoelous neck vertebrae can also be found in rhinos (e.g., the fossil *Stephanorhinus etruscus*, IGPB M2828). The degree of concavity of the posterior neck vertebra surface is comparable in giraffes and rhinos, and less concave in the reindeer. The distribution of bony vertebral centra epiphyses in the wild boar (*Sus scrofa*, IGPB M56) is as in the reindeer from the posterior end of the axis to the sacrum. Contrary to reindeer, giraffes, and rhinos, the wild boar cervical vertebrae show platycoelous centra, as do small mammals like hedgehog, vole, and mole. Kemp (*67*) lists this character of the platycoelous vertebral centrum for Therapsida. For Neotherapsida, he furthermore lists the character "epiphyses on atlas vertebra" (*67*) which suggest the presence of bony vertebral centra epiphyses in Neotherapsida or even more primitive synapsids.

In humans the situation is slightly more complex. As Dar et al. (*65*) summarise(*68*) summarize, there is a cartilaginous apophysis (incorrectly called an 'epiphyseal ring'), which ossifies itself, but is not a true epiphysis, because it does not contribute to vertebral centrum growth. The lack of true bony vertebral centrum epiphyses in humans might be a result of our bipedal locomotion.

1.5 Cartilaginous tissues in intervertebral joints and their relationship to bone

The background information provided in this and the next section is textbook knowledge that is necessary, however, for understanding our inferences on soft part anatomy in the fossils. Recommended references are Francillion-Vieillot et al. (*34*) and Hall (*2*).

Cartilaginous tissue can be differentiated into three types which all contain chondrocytes and which vary with respect to the composition of the extracellular matrix (ECM). The first is the hyaline cartilage (HC), second the elastic cartilage (EC), and third the fibrocartilage (FC). Cartilaginous cells are derivatives of mesenchymal cells, which differentiate into chondroblasts and finally chondrocytes. Here we discuss only the HC and the FC because the EC, which is characterised by the presence of elastic fibers, is not relevant for IVDs and other intervertebral joints.

Hyaline cartilage is seen, e.g., in joint articular surfaces, in the rib cartilage and in the tracheal cartilage. In diarthroses, which represent true articulations of bone with an intra-articular space, the articular facet is covered by HC. For HC, synovial fluid is essential because of the avascularity of HC. HC extracellular matrix contains collagen type II, proteoglycans, and water. The interactions of different components can be described by the Benninghoff-Arkaden module, which explains the architecture of the HC as an elastic spring and therefore makes the HC pressure elastic, which means that the HC is reversibly compressible. This fact is important for a diarthrosis joint.

Except for the diarthrosis joint, HC is also the product of the perichondrium which surrounds all areas of cartilage except for joint surfaces. The perichondrium may be differentiated into two different layers, first the stratum fibrosum which is a collagen-fiber connective tissue providing resistance of the cartilage against shear stress, and second a layer called the stratum chondrogenicum which contains mesenchymal cells which can differentiate into chondroblasts. The perichondrium is the source of appositional growth in length of skeletal elements.

In humans, the fibrocartilaginous tissue is seen in the annulus fibrosus (AF) of the IVD, the pubic symphysis, in articulation discs and in the meniscus of the knee joint. The difference between HC and FC is that fibrous cartilage contains collagen I fibers in addition to cartilage-specific collagens like collagen II, whereas hyaline cartilage does not. In the IVD we see that the fibers of the AF extend in spiral torsion from one vertebra to the next vertebra. Between the subchondral lamella of a vertebra and the AF there is a layer of HC. The joint between two vertebrae in mammals is a synarthrosis as it is lacking an articular cavity. The IVD also contains the nucleus pulposus (NP) which is surrounded by the AF.

The AF and NP are specific relationships with the cartilaginous endplate and the bony endplate which overlie each other and are both part of the endochondral domain of the vertebral centrum. The AF must be deeply anchored into the vertebral endplate (bony or cartilaginous) because they take up the tensional forces exerted on the IVD. Thus, the fibres of the AF insert into the bony endplate at the angle and orientation they have in the IVD. This is accomplished by insertion into the serial cartilage of the endplate and into the bony spicules that separate the files of serial cartilage. This has been described in numerous histological studies of IVDs, and good illustrations can be found in Shu et al. (*42*, fig. 2) for sheep and Onishi et al. (*43*, fig. 7) for mice. This histological situation is preserved in fossils whereas the AF itself is not. It thus is used in this study to infer the location and angle of insertion of the AF in fossils. Note that the anchoring fibers of the AF are different from Sharpey’s fibres which by definition the insertion of ligaments and tendons into periosteal bone.

The NP as the ‘cushion’ in the IVD structures only takes up compressional forces and thus does not requiring anchoring by fibers. Accordingly, the endplate adjacent to the NP, i.e., in the center of the articular surface of the vertebral centrum, lacks a fibrous connection with the NP. The cartilage in this regions typically consists of poorly organised chondrocytes that lack the strict linear arrangement of those in the region of AF insertion (see Shu et al. (*42*, fig. 2) and Onishi et al. (*43*, fig. 7). Thus, these patterns of cartilage cell and bone arrangement in thin sections of fossil vertebral centra can be used to infer the presence and shape of an IVD. The patterns we observed are very consistent between different taxa.

1.6 Bone tissues of the amniote vertebral centrum

Ossification of the amniote vertebral centrum starts by replacement of embryonic cartilage by bone. Specifically, cartilage is resorbed and bone-forming cells (osteoblasts) secrete bone tissue in its place (endochondral bone formation). However, at the anterior and posterior end of the embryonic centrum, new cartilage continues to be formed by cartilage-forming cells (chondroblasts), producing a growth front (growth plate) that underlies the joint between adjacent centra. The part of the centrum that results from this process is the endochondral domain. The process of endochondral bone formation continues until growth of the centrum stops. In some mammals, the process of endochondral ossification also takes place towards the joint, resulting in a secondary center of ossification or bony epiphysis (see section 1.3.1). When growth ceases, the secondary center of ossification fuses with the rest of the centrum. The surface of the endochondral bone, forming the articular surface in vertebral centra and long bones, is typically rough or pitted, reflecting the cartilage cover of the bone. Endochondral bone surfaces are commonly called “unfinished” in paleontology (*1*).

The cartilage growth front consists of a differentiation part, a proliferation part, and a hypertrophication part. Chondroblasts differentiate from more generalised cells in the differentiation part and divide in the proliferation part, forming characteristic files of cartilage cells (chondrocytes) that are oriented roughly in the direction of growth. Older chondrocytes increase in size and mineralise gradually a hydroxyapatite ECM, providing a local source for the components of the bone matrix that will replace them. This tissue of enlarged chondrocytes in the hypertrophication zone commonly is called “calcified cartilage”. This has led to confusion in the past with cartilaginous tissues that are more highly and regularly mineralised and are never replaced by bone, such as in the endoskeleton of chondrichthyans (see above). True calcified cartilage is rare in amniotes and was not observed in our study. The cartilage differentiation zone is also the source of the hyaline cartilage of the amniote joint (but not the NP). The fibrocartilage of the joint inserts in the endochondral domain. The tissue showing files of chondrocytes is also called “serial cartilage”.

Returning to the early growth of the vertebral centrum, soon after the onset of endochondral ossification, the embryonic cartilage core will be surrounded by a bone-producing connective tissue containing osteoprogenitor cells and osteoblast, the periost. Bone produced by the periost does not have a cartilage precursor but is deposited layer by layer, producing the growth in girth of the vertebral centrum. The ligaments of the joint capsule and intervertebral ligaments exclusively insert in the periosteal bone. The insertion sites remain visible in the bone tissues as Sharpey’s fibers. The different mechanisms of tissue formation between the endochondral bone and the periosteal bone result in a sharp border between the two domains that is preserved until it is obliterated by secondary remodeling. Morphogenesis of the vertebral centrum thus consists of the interplay between growth in the endochondral domain and the periosteal domain. Long bones of amniotes show the same growth mechanism and domains. The surface of the periosteal bone is typically smooth and only pierced by vascular foramina. Periosteal bone surfaces are commonly called “finished” in paleontology (*1*).

2. Specimen descriptions

*2.1. Diadectomorpha*

2.1.1. *Diadectes sideropelicus*, IGPB A 169, Lower Permian, Texas, USA (Figure S1, S2)

2.1.1.1 Morphological observations

The stem amniote *Diadectes* has deeply amphicoelous vertebral centra with a distinctive foramen connecting the anterior and posterior articular facets of the centra. The foramen is generally interpreted as allowing the notochord to pass through. The vertebral centrum is roughly isometric, with a diameter to length ratio of about 1. The articular surface consists of a concave, funnel-shaped central part and a convex outer rim that is sharply set off from the outer, smooth surface of the centrum.

2.1.1.2 Histological observations

The sagittal section of the adult dorsal vertebra confirms the deeply amphicoelus nature of the centrum. However, the section failed to intersect the open notochordal canal because of imprecise processing. The thickness of the endochondral bone underlying the articular surface increases gradually from the center to the periphery of the articular surface, seen as four wedges of endochondral bone radiating outward from the growth center of the vertebra. Thus, throughout growth, the relative thickness of each domain of endochondral bone remains constant. The deeply amphicoelous shape of the centra thus is maintained from the onset of ossification until the termination of growth. The amphicoelous shape thus does not result from the limited production of endochondral bone in the articular area, unlike in all but the most basal ichthyosaurs (*23*). While the boundary between the periosteal and endochondral domain forms a straight line, the outer part of the anterior and the posterior surface is convex, forming a rim around the articular surface.

The concave, “funnel” region of the articular surface lacks any indication of chondrocyte lacunae but is covered by a smooth layer of lamellar bone tissue instead. On the convex outer rim of the articular surface, chondrocyte lacunae are arranged in short, loose files, and there are bone spicules with fibers in between the files. Both ventrally and dorsally, the region of the periosteal bone adjacent to the boundary with the endochondral domain is rich in Sharpey’s fibers. These are directed roughly parallel with the domain boundary, intersecting the surface of periosteal bone at a high angle.

2.1.1.3 Interpretation

The amphicoelous shape indicates that there was no synovial joint in *Diadectes* because in this type of joint there is a close morphological match of the two articular surface that form the joint. The distinct notochordal canal (not bisected by the sample but seen in the specimen) also is inconsistent with a NP, as is the smooth bone surface of the “funnel”. There must have been some other kind of fill, presumably derived from the notochord. We interpret the remains of cartilage and bony spicules on the convex ring of the articular surface as the anchoring site for fibrocartilage, forming an AF. The direction of the fibers in the periosteal bone is consistent with them forming the insertion of a ligamentous connection between the vertebral centra.

2.1.1.3 Discussion

Based on the phylogenetic position of *Diadectes* as a stem amniote, we infer that it is the intervertebral joint of this taxon from which evolved the other kinds of intervertebral joints in amniotes. The vertebrae were connected by an AF on the outer part of the articular surface and by intervertebral ligaments inserting in the periosteal surface. The notochord persisted throughout ontogeny and continued to increase in girth in the intervertebral space in postembryonic and post-hatching ontogeny.

*2.2. Synapsida*

2.2.1. *Dimetrodon natalis* IGPB R 652a, 653a, 658 Lower Permian, Texas, USA (Figure S3, S4)

2.2.1.1 Morphological observations

As described in detail by Romer & Price (*69*), *Dimetrodon* has deeply amphicoelous vertebral centra with a small foramen connecting the anterior and posterior articular facets of the centra. The foramen is generally interpreted as related to the notochord.

2.2.1.2 Histological observations

Sagittal sections of juvenile and adult dorsal vertebrae (IGPB R 653a and IGPB R 652a) and of two fused sacral vertebrae (IGPB R 658) confirm the deeply amphicoelous nature of the centra and the open notochordal canal. The thickness of the endochondral bone underlying the articular surface increases gradually from the center to the periphery of the articular surface, seen as four wedges of endochondral bone radiating outward from the notochordal foramen. Thus, throughout growth, the relative thickness of each domain of endochondral bone remains constant, making up about one eight of the length of the centrum. The deeply amphicoelous shape of the centra thus is maintained from the onset of ossification until the termination of growth. While the boundary between the periosteal and endochondral domain forms a straight line, the outer part of the anterior and the posterior surfaces are convex, forming a rim around the articular surface.

Even in the juvenile, there is little retention of the original chondrocyte lacunae in the endochondral bone. However, the articular face is covered by a thin layer of chondrocyte lacunae that are arranged in an irregular fashion. These represent the hyaline cartilage cover of the articular surface, i.e., the active chondroblast differentiation zone of the growing vertebra. In the adults, there are no chondrocyte lacunae left except in the notochordal canal and the convex outer rim of the articular surface. There, the lacunae are arranged in short, loose files, and there are bone spicules with fibers in between the files. In the region forming the concave, “funnel” part, the articular surface of the adult actually is covered by a smooth layer of lamellar bone tissue. Both ventrally and dorsally, the region of the periosteal bone adjacent to the boundary with the endochondral domain is rich in Sharpey’s fibers. These are directed roughly parallel with the domain boundary, intersecting the surface of periosteal bone at a high angle.

2.2.1.3 Interpretation

The amphicoelous shape indicates that there was no synovial joint in *Dimetrodon* because in this type of joint there is a close morphological match of the two articular surface that form the joint. The layer with the irregularly arranged chondroblast lacunae represent the hyaline cartilage cover of the articular surface, i.e., the active chondroblast differentiation zone in the juvenile. In the adults, the lack of a layer of cartilage and the smooth bony surface of the concave part of the articular surface suggest that there was no NP yet but some other kind of fill, presumably derived from the notochord. The lamellar bone layer in the “funnel” is particularly striking because it is not observed in any of the other samples*.* We interpret the convex ring of the articular surface as the anchoring site for fibrocartilage, forming an AF. The direction of the fibers in the periosteal bone is consistent with them forming the insertion of a ligamentous connection between the vertebral centra, possibly forming some kind of joint capsule.

2.2.1.4 Discussion

It has been consensus that basal synapsids such as *Dimetrodon* did not possess synovial vertebral joints (*69*). This is confirmed by our observations, in particular the persisting notochord. The intervertebral space of *Dimetrodon* must have looked very similar to that of amphicoelous geckos which was described in detail by Holder (*18*). In these, the notochord is also continuous, increasing in diameter in the intervertebral spaces and decreasing as it passes through the growth center of the centrum. From an ontogenetic perspective, this means that the embryonic notochord has retained its diameter there but continued to grow in girth in the intervertebral space.

2.2.2. *Phoca vitulina* IGPB M 60, recent (Figure S5)

2.2.2.1 Morphological observations

*Phoca vitulina*, the common European seal, is represented in our sample by a thoracal vertebra that is part of a complete skeleton. Although the specimen is an adult, there is still a distinctive suture at either end of the vertebral centrum between the secondary centers of ossification (bony end plates, epiphyses) and the body of the centrum. The specimen is extant and maceration has removed any soft parts. The open epiphyseal suture would indicate immaturity in a terrestrial mammal but is a paedomorphic feature seen in adults of marine mammals.

2.2.2.2 Histological observations

In the sagittal section, the sutures between the epiphysis and the body of the centrum are also distinctive and there is an open slit, not containing any soft parts. The surface facing the suture shows hyaline cartilage of the proliferation zone that then is transformed into bony trabeculae. The same situation is observed on the vertebral centrum facing the suture. The articular surface of the epiphysis is covered by a thin layer of cartilage cells partially embedded in bone matrix. The layer is at most three cells thick over most of the epiphysis. Only towards the perimeter, the layer becomes thicker, and the cartilage cells are arranged in files with collagen fibers in between. The direction of the files and fibers in this region is obliquely outward from the center of the articular surface. The cartilage cells are distinctly irregular in size. The perimetral region extends right up to the epiphyseal suture.

2.2.2.3 Interpretation

As for all therian mammals, it can be assumed that the specimen possessed an intervertebral disc. However, the macerated specimen offers no direct histological evidence for the AF and NP but was included to aid in interpretation of the fossil samples. The thin layer of cartilage cells on the epiphysis is the remainder of the cartilaginous endplate, and the structures seen along the perimeter are the attachment of the fibrocartilage of the AF of the IVD. The direction of the fibers and files is the same as those in the AF, in a circumferential arrangement around the NP, as seen in soft tissue histological sections of mammals. The geometry of the NP with a radius that is significantly smaller than that of the endplate and the geometry of the AF means that the angle of insertion of the fibers of the AF and the inclination of the associated cartilage cell files changes towards the periphery of the end plate. The central area of the cartilaginous endplate that lacks fiber insertions maps out the extent of the NP.

2.2.2.4 Discussion

A histological ground section of a macerated specimen of an extant mammal offers indications of some of the soft parts originally present (i.e., cartilaginous endplate, AF, NP) and their spatial arrangement. We then used this information to infer the types and distributions of intervertebral tissues in fossils.

2.2.3 cf. *Eurohippus* HLMD-ME 103, 106, 7835, middle Eocene, Messel, Germany (Figure S6)

2.2.3.1 Morphological observations

Three articulated specimens of ungulates from the famous Eocene lake deposits of Messel (Germany) were sampled in the hope of finding preserved soft parts, for which the locality is famous for, in the intervertebral space. Two of the three specimens are embedded in an artificial matrix and showed evidence of soft part preservation.

2.2.3.2 Histological observations

Two (HLMD-ME 103, 7835) of the three Messel specimen were extremely crushed, showing a near-complete collapse of the trabecular architecture and complete compaction of the internal vascular spaces. Nevertheless, fragments of the bony endplates were sufficiently preserved to show the same features as the *Phoca* vertebra, in particular the distinction between the inner region with very little cartilage and the perimeter with thicker cartilage, cells arranged in files and fibers parallel to these files. The outward direction of these features is the same as in *Phoca.* However, none of the three Messel mammals show an epiphyseal suture zone. Also, none of the Messel mammal specimens showed any indication of the preservation of soft parts of the intervertebral space such as the AF and the NP.

2.2.3.3 Interpretation

The observed histological features are the same as in the extant mammal, *Phoca*, and suggest that the Messel mammals possessed a mammalian-type IVD. The lack of epiphyseal sutures in the Messel mammals indicates that the sampled individuals were adults.

2.2.3.4 Discussion

Apart from the extreme crushing, the preservation of the Messel mammal samples is similar to the macerated extant mammal in our sample. This is somewhat surprising because the articulated bones of two (HLMD-ME 103, 7835) of the three Messel specimens are partially covered by a black carbonaceous film, typically interpreted as soft part preservation. However, the film may mainly represent integumentary tissue, which is what the soft parts typically reported from Messel (fur, feathers, patagia, ears) consist of. Possibly, internal maceration of the carcass on the lake floor destroyed all intervertebral soft tissues.

*2.3. Mesosauria*

2.3.1. *Stereosternum tumidum* IGPB R 622, Irati Formation, Early Permian, Brazil (Figure S7)

2.3.1.1 Morphological observations

*Stereosternum tumidum* is one of three species of the Mesosauridae that differ in patterns of pachyostosis of the postcranial axial skeleton, i.e., vertebrae and ribs. The centra, neural arches, and ribs of *Stereosternum tumidum* are pachyostotic, as seen specimen IGPB R 622. Although Romer (1956) (*1*) described the centra as “notochordal”, the morphology of the articular surfaces of the centra has not been described in detail before. In *Stereosternum tumidum* IGPB R 622 we observed dorsoventrally flattened vertebral centra that are about as long as wide with amphicoelous articular ends.

2.3.1.2 Histological observations

Sagittal section of an articulated set of four anterior dorsal vertebrae, including three intervertebral spaces, reveal elongate centra with a length to height ratio of 4:1. The sections show that the centra are clearly amphicoelous but that the two “funnels” do not approach each other unlike in all of the other amphicoelous vertebrae in this study. Instead, the “funnels” are connected by a long notochordal canal that reaches 3/4 of the length of the centra. Interestingly, the canal is wider at the center of growth than towards the articular surfaces. The canal is surrounded by endochondral bone with a high density of osteocyte lacunae. The endochondral bone shows a complex, seemingly whorled arrangement of vascular canals. The dorsal part of the central appears lack periosteal bone entirely while this domain is well expressed in the ventral part.

There is little persistence of the original chondroblast lacunae in the endochondral bone, with only a few cartilage islands remaining. However, the articular face is covered by a layer of chondrocyte lacunae that are arranged in an irregular fashion and sometimes connect. In the outer part the articular surface, there are bony spicules with fiber insertions that are directed longitudinally.

The notochordal canal and its exit region into the articular facet contain large, translucent, crystalline matter. However, most of the intervertebral spaces is filled up by opaque matter. In polarised light, this matter is distinctive from both, the fill of the neural canal (which consist of globular bodies resembling fecal pellets) and the sediment matrix. In polarised light, the opaque matter has a brownish rim. The periosteal bone of the centra shows no indication of intercentral connective tissue, neither dorsally nor ventrally.

2.3.1.3 Interpretation

The long and distinctive notochordal canal of the vertebrae of *Stereosternum tumidum* must have housed the notochord, preserved as translucent, coarsely crystalline matter that also fills the central region of the articular facet. The opaque matter may represent the AF, the attachment of which to the centrum is represented by the fiber insertions in the outer part of the articular surface.

2.3.1.4 Discussion

The centra of *Mesosaurus* thus show an unusual combination of amphicoelous articular surfaces and elongate centra with a long notochordal canal. The lack of dorsal periosteal bone is also unusual. The pattern of the notochord persisting in mesosaurs thus is the same as in *Dimetrodon.*

*2.4. Hupehsuchia*

2.4.1 *Nanchangosaurus suni* HFUT YAN-10-02, Early Triassic, Hubei, China (Figure S8)

2.4.1.1 Morphological observations

In the specimen it can be observed that the dorsal neural arches of *Nanchangosaurus* are pachyostotic, as are the ribs. This accords with the description of Chen et al. (*42*). In this way, the taxon resembles the basal sauropterygian *Neusticosaurus* discussed below. The centrum morphology of *Nanchangosaurus* was not described by Chen et al. (*42*), however.

2.4.1.2 Histological observations

In the histological sections, the centra are seen to be deeply amphicoelous but not notochordal. The endochondral domain is thin compared to the periosteal domain. The latter is poorly vascularised by radial canals, and the former consists of thick trabeculae resulting in limited vascular space. The microanatomy thus suggests bone mass increase. The endochondral bone has some interstitial globular bodies representing hypertrophied cartilage. Towards the articular surface, this is followed by a 100 µm thick layer of irregularly arranged globular bodies that lack any evidence of intervening mineralisation. The transition to the remaining intervertebral space is very irregular. The intervertebral space is filled up by granular material that consists of round translucent grains with brown matter in between. The grains are distinctly larger than the globular bodies. In one region of contact between the granular material and the globular bodies, there is a brown patch of seemingly organic material. The brown patch is semitranslucent and contains striations and spindle-shaped cells. Under polarised light, it remains brown and is not birefringent and thus is not bone. The anterior and posterior parts of the thick and unresorbed periosteal cortex show thin SF.

2.4.1.3 Interpretation

The layer of irregularly arranged globular bodies on top of the endochondral bone must represent the proliferation zone of the cartilage, i.e., hyaline cartilage, or possibly fibrocartilage because of the very irregular arrangement and wide spacing of the cells. The granular material could either represent mineral grains of diagenetic origin that replaced the original organic matter or it could represent fibrocartilage. Another option is that it represents altered notochordal material. We prefer this latter option because of the brown patch that is closer in appearance to fibrocartilage than the granular material. In addition, the grains in the granular material are distinctly larger than the chondrocytes of the hyaline cartilage. The coarse granular material thus possibly represents an NP, and the brown patch possibly represents the remains of an AF. Thus, the evidence suggests that *Nanchangosaurus* possessed an IVD with a large NP.

2.4.1.4 Discussion

The intervertebral articulation anatomy and histology of *Nanchangosaurus* thus differs from all of the previously discussed taxa except for the mammals in that the notochord is no longer continuous but forms a large NP.

*2.5. Ichthyosauria*

2.5.1 Cymbospondylidae new taxon A, LACM DI 158109, Middle Triassic, Nevada, USA (Figure S9)

2.5.1.1 Morphological observations

We studied a string of anterior dorsal vertebrae that are from an originally complete and only slightly disarticulated skeleton of a new cymbospondylid ichthyosaur from the Middle Triassic Fossil Hill Member of the Favret Formation, Augusta Mountains, Nevada, USA. All ichthyosaurs have deeply amphicoelous centra but the notochordal foramen is vestigial if at all present (*1*, *23*). Vertebral shape changes in ichthyosaur evolution from vertebral centra that are longer than high in the most basal taxa (*70*), the plesiomorphic condition in reptiles, to disc-shaped centra that may be twice as high as long, or more. The disc-shaped vertebrae are first seen in the cymbospondylids of the Middle Triassic and are present in all Neoichthyosauria.

2.5.1.2 Histological observations

The histology of one of the vertebrae in this string was briefly described and illustrated by Houssaye et al. (*23* fig. 3G,H). The deeply amphicoelous shape results from the limited of production of endochondral bone in the articular area. This is also seen in some other ichthyosaurs, where there is a negative allometry of the relative amount of endochondral bone contribution (*23*). In the new cymbospondylid, the outermost layer of mineralised material surrounds globular spaces that obviously were occupied by chondroblasts in the living animal. The chondroblast spaces are open towards the intervertebral space, suggesting a cover of the articular surface of the vertebra by hyaline cartilage.

The segment of four anterior dorsal vertebrae is of particular interest because of the matter preserved in the intervertebral spaces. There are different types of matter that we interpret as altered intervertebral tissues. The hyaline cartilage described above is overlain by clear sparry calcite that differs from normal calcite in that it appears to have incorporate a fine fibrous matter during crystal emplacement. In the center of this dark matter, there is a round structure consisting of globules of fine-grained material incorporating some irregularly shaped brownish matter. The primary periosteal bone is cancellous but it does contain Sharpey’s fibers closest to the articular surface but not in the region in between.

2.5.1.3 Interpretation

We interpret the fibrous matter in the sparry calcite as altered fibrocartilage, representing the AF, and the central, round structure with the globules as the altered NP. The coarse fabric may reflect the much larger cells of notochordal origin that form the NP. The intervertebral spaces in the vertebral column of the new cymbospondylid ichthyosaur were thus occupied by an intervertebral disc attached to the cartilaginous endplates of the vertebral centra. The endplates consisted of a layer of actively proliferating hyaline cartilage. The intervertebral ligaments or the joint capsule appears to have been weakly developed.

2.5.1.4 Discussion

The Fossil Hill Member is a black shale unit characterised by the preservation of complete marine reptile skeletons (e.g., *71*-*73*) and the preservation of soft parts thus appears possible. The interpretation of the intervertebral matter as altered intervertebral tissue may appear far-fetched at first, and alternative hypotheses need to be examined that would produce the observed pattern. Probably, bacterial activity altered the original intervertebral tissues, but only at the microscopic level, leaving the larger anatomical structures intact. The issue will be examined in more detail in the discussion of the *Stenopterygius* specimen described below.

2.5.2. cf. *Cymbospondylus* sp. IGPB R 660, Lower Triassic, Spitsbergen (Figure S10)

2.5.2.1 Morphological observations

The dorsal vertebrae of cf. *Cymbospondylus* are disc-shaped, i.e., shorter than dorsoventrally tall, and deeply amphicoelous with an even slope towards the growth center. They appear to lack a notochordal foramen, however small.

2.5.2.2 Histological observations

The endochondral domain is thin compared to other early ichthyosaurs (*23*), and there is only a thin layer of hyaline cartilage facing the intervertebral space. The intervertebral space is filled with matrix not any different from the surrounding matrix. However, an isolated patch of brown material with globular holes can be observed in the matrix in the intervertebral space. The histological section confirms the morphological observation that the vertebrae are not notochordal. The anterior and posterior one tenth of the very thin periosteal cortex of the centrum shows Sharpey’s fibers, which are better developed ventrally than dorsally.

2.5.2.3 Interpretation

The globular bodies covering the articular faces are interpreted as hyaline cartilage. The patch of brown material with globular holes is interpreted as altered fibrocartilage. The Sharpey’s fibers suggest the presence of an intervertebral ligament.

2.5.2.4 Discussion

The thin layer of hyaline cartilage is the same as in the previous ichthyosaur samples. The patch of fibrocartilage probably derives from the AF, but no evidence of the NP is preserved. The hypothesis of the preservation of altered soft parts in the intervertebral spaces is amenable to testing by examination of modern carcasses from anoxic environments. In addition, ultrastructural and organic geochemical investigations could be very informative.

2.5.3. *Stenopterygius* sp. IGPB R 661, Posidonienschiefer Formation, Lower Jurassic, Holzmaden, Germany (Figure S11, S12)

2.5.3.1 Morphological observations

We studied a string of anterior dorsal vertebrae that are from an originally complete and only slightly disarticulated skeleton of *Stenopterygius* sp. from the Early Jurassic Posidonienschiefer Formation of Holzmaden, Germany. This is one of the classical ichthyosaur faunas. As all of the other ichthyosaurs studied here, the *Stenopterygius* vertebrae have deeply amphicoelous, disc-shaped centra.

2.5.3.2 Histological observations

In our specimen of *Stenopterygius*, the layer of endochondral bone underlying the articular face of the vertebrae is thin (less than 1 mm) and did not increase in thickness ontogenetically. The outermost layer of mineralised material surrounds globular bodies that obviously were occupied by chondroblasts in the living animal. The chondroblast spaces are open towards the intervertebral space, suggesting a cover of hyaline cartilage of the articular surfaces of the vertebra. In the center of the articular face, the globular bodies are arranged in poorly defined, surface-parallel layers. Towards the perimeter, the globular bodies become increasingly organised into files that diverge increasingly until they are at an angle of 45º to the centrum long axis in the outermost perimeter. The primary periosteal bone is thin but does contain Sharpey’s fibers closest to the articular surface but not in the region in between.

The segment of six anterior dorsal vertebrae is of particular interest because of the matter preserved in the intervertebral spaces. The laminated sediment matrix of the specimen did not enter these spaces. Instead, there are different types of matter that we interpret as altered intervertebral tissues. The hyaline cartilage described above is overlain by dark, fine-grained material which takes up the greater part of the intervertebral space. In the center of this dark matter, there is a round structure of translucent matter that shows a coarser fabric.

2.5.3.3 Interpretation

We interpret the dark matter as altered fibrocartilage, representing the AF, inserting into the inclined files of globular bodies, representing chondrocytes. The central, round translucent structure probably represents an altered NP that was separated by the bony part of the centrum by a layer of surface-parallel cartilage. The coarse fabric probably reflects the much larger cells of notochordal origin that form the NP. The intervertebral spaces in the vertebral column of *Stenopterygius* were thus occupied by an IVD attached to the cartilaginous endplates of the vertebral centra. The endplates consisted of a layer of actively proliferating hyaline cartilage. The intervertebral ligaments or the joint capsule appears to have been weakly developed.

2.5.3.4 Discussion

As noted in the description of the Middle Triassic cymbospondylid LACM 158109, we interpret the intervertebral matter as altered intervertebral tissue. Although localised activity of degrading and scavenging organisms could have caused the observed pattern, the activity of most any other organism than bacteria can be excluded for the Posidonienschiefer Formation because of the laminated, anoxic nature of the sediment (*74*). In fact, the lamination indicates the lack of any metazoan activity. It also appears unlikely that scavenging metazoans would have been able to be active in the intervertebral spaces if the entire surrounding sediment was anoxic. Probably, bacterial activity altered the original intervertebral tissues, but only at the microscopic level, leaving the larger anatomical structures intact.

The comparison with the matter found in the intervertebral spaces of LACM 158109 is important because both specimens show the same pattern, i.e., a ball-shaped structure in the center of the space which is surrounded by different material, setting the “ball” off from the cartilage endplate. This material also completely fills the remaining intervertebral space, close to the margins of the articular surface.

The hypothesis of the preservation of altered soft parts representing an AF and a NP in the intervertebral spaces is amenable to testing by examination of modern carcasses from anoxic environments at the bottom of euxinic lakes and marine basins. In addition, ultrastructural and organic geochemical investigations could be very informative.

2.5.4. *Leptopterygius* sp. IGPB R 235, Lower Jurassic, Lyme Regis, England (Figure S13)

2.5.4.1 Morphological observations

The probable anterior caudal vertebrae are extremely crushed but are similar in size, morphology, and histology to the *Cymbospondylus* ones.

2.5.4.2 Histological observations

The endochondral domain is thin, as in other Jurassic ichthyosaurs (*23*), and there is only a thin layer of hyaline cartilage facing the intervertebral space. One of the vertebrae is intersected in its exact growth center and seen to be notochordal, with an interruption in bone tissue. This area in the growth center of the centrum contains several globular spaces with a dark center.

2.5.4.3 Interpretation

The globular spaces with a dark center may be preserved notochordal cells. They are different from the hyaline cartilage of the articular face.

2.5.4.4 Discussion

The thin layer of hyaline cartilage is the same as in the previous ichthyosaur samples. However, the caudal vertebrae differ from the previous three ichthyosaur samples in the presence of the notochord. Retention of notochordal material is surprising for a derived Jurassic ichthyosaur, especially against the background of the non-notochordal cf. *Cymbospondylus* vertebrae. However, the explanation may lie in the posterior position of these vertebrae. This hypothesis is open to testing by further sampling of caudal vertebrae of other ichthyosaur taxa. At least in non-archosauromorph reptiles, the notochord persists even in the adult tail to facilitate regeneration after autotomy (*31*). This is indicated by the basal reptile *Captorhinus* and extant Lepidosauria. These two forms the phylogenetic bracket of Ichthyosauria. We do not suggest, however, that ichthyosaurs performed caudal autotomy.

*2.6. Sphenodonta*

2.6.1. *Sphenodon punctatus* NHMW 8108:2, New Zealand, recent (Figure 1, Figure S14)

2.6.1.1 Morphological observations

As noted by Romer (*1*), the dorsal vertebrae of *Sphenodon* are amphicoelous and notochordal. A complete skeletal specimen (Collection of the Institute of Anatomy, University of Cologne) confirms these observations which are difficult to make in the partially dissected alcohol specimen (NHMW 8108:2) that we sampled histologically. This type of morphology differs from that of more derived lepidosaurs, i.e., squamates, that have evolved a ball-and-socket joint, specifically the opisthocoelous condition (with the exception of some geckos). We observed small ventrally located intercentra, as already stated by Romer (*1*).

2.6.1.2 Histological observations

Sagittal microtome histological sections of the dorsal vertebrae show the nature of the intervertebral discs and the vertebral centrum well. The spongiosa in the center of the vertebral bodies is trabecular and there is only a thin cortex of periosteal bone. The endochondral bone is covered at its cranial and caudal end by a layer of serial cartilage, i.e., hypertrophied cartilage, but the proliferation zone is not distinct. There is a diffuse boundary with the fibrocartilage of the intervertebral joint. The fibrocartilage forms a ring connecting the margins of the articular surfaces of the centra in a structure similar to the AF of mammals. the notochord forms a diabolo-shaped core structure within the elongate vertebral bodies. In the middle, the notochord ossifies to form an intravertebral septum. Remarkably, in the intervertebral space, the notochord forms a conspicuous cavity called *vesicular centralis* (vesicle of Romer) (*1*). We identified a layer of hyaline cartilage between the fibrocartilage and the bony vertebral centrum. The small intercentra are well visible in the sections and are in functional combination with the fibrocartilage ring.

2.6.1.3 Interpretation and discussion

The *Sphenodon* material is very helpful in interpreting the fossils. The uneven surface of the cartilage cover of the endochondral bone is suggestive of attachment of fibrocartilage which preserves much less frequently in the fossils than the serial and hyaline cartilage. The distribution of soft tissues in the intervertebral space of *Sphenodon* dorsal centra fits the inferences that could have been made for this sample based on hard tissues alone. This supports our inference for fossil vertebrae of similar morphology and histology, e.g., those of *Diadectes*, *Dimetrodon*, and *Captorhinus*. *Sphenodon* thus shows the plesiomorphic amniote condition of the intervertebral joint.

The development of the embryonic vertebral column of *Sphenodon* had already been figured by Howe and Swinnerton (Romer 1956, fig. 116) (*1*), and the adult vertebral histology was described and figured by Wettstein (*75*). Our observations largely accord with his observations and those of Schauinsland (*76*). However, these authors did not report the layer of hyaline cartilage between the fibrocartilage and the endochondral bone. The layer might have escaped the attention of Wettstein for the lack of Azan staining.

*2.7. Squamata*

2.7.1 *Mosasaurus missouriensis* IGPB Goldfuß 1230, Late Cretacous, North Dakota, USA (Figure S15)

2.7.1.1 Morphological observations

Like all Mosasauroidea and their possible sister taxon Ophidia, *Mosasaurus* dorsal vertebral centra articulate via well-developed ball-and-socket joints, with the socket formed by the anterior articular facet, thus representing the procoelous condition. In other vertebrae of the same skeleton, it can be seen that both the ball and the socket have very smooth and dense surfaces, without any pores or rugosities. This is apparent in other mosasaur vertebrae as well.

2.7.1.2 Histological observations

The vertebral column appears to have been preserved in a hard-calcareous concretion, and at the time of the original description by Goldfuß in 1845 (*77*), the vertebrae could only be free from the matrix in a crude way, leading to much damage of the bone surface. However, the concretionary development lead to excellent three-dimensional preservation. Accordingly, the thin sections reveal that both pairs of vertebrae remain tightly articulated, and the intervertebral space is one mm or less wide. Both articular surfaces, the convex (ball) and concave (socket) one shows a continuous, smooth bone layer, as was evident from exposed joint surfaces of the other vertebrae. The smooth surface is seen to consist of compact bone covered by a very thin semi-translucent beige-brown layer with a globular texture. The actual intervertebral space is filled with a dark gray, essentially opaque carbonate matrix that is interrupted by voids set at irregular distance from each other. At high magnification, the layer is evenly translucent without any evidence of mineralised matrix and some of the globular bodies contain small brown spots. The periosteal bone of the centra shows SF near the articular region, paralleled by vascular canals and better developed in the ventral region.

2.7.1.3 Interpretation

Because of its similarity to the extant snake intervertebral joint described below, the interpretation of the mosasaur vertebrae is straightforward. Like in the snake, the mosasaur clearly had a synovial joint. The beige-brown layer must be altered hyaline cartilage of the joint surface. The mineral infilling of the thin intervertebral space would have been occupied by synovial fluid in life. The small brown spots in the chondrocytes may represent cell nuclei fossilised in the process of cell division because they resemble the dark-staining nuclei in the hyaline cartilage of the *Python* vertebrae. Evidence for intervertebral ligaments is stronger in the ventral region.

2.7.1.4 Discussion

While the nature of the intervertebral joint could have been inferred with some confidence from phylogeny and osteological correlates, support from direct observation is important. The specimen is also important because it again shows that intervertebral unmineralised tissues may survive in an altered state, in this case the layer of hyaline cartilage covering the bony joint surface.

2.7.2 *Python* sp. IGPB R 662, recent (Figure S16)

2.7.2.1 Morphological observations

The procoelous vertebrae of snakes were mentioned already, and the specimen we sampled shows this very well in histological microtome sections. In fact, the procoelous condition could only be observed in the section because the studied vertebrae were not dissected out of the soft tissue.

2.7.2.2 Histological observations

Both the sagittal and the frontal section of the snake vertebrae show the clear procoelous morphology with a tightly fitting ball-and-socket joint. The intervertebral space is only a few hundred µm wide or even, in the median plane, not detectable. However, the width of this space may have been altered during microtome sectioning. Both joint surfaces can be seen to be made up of hyaline cartilage of the proliferation zone with the cells arranged in columns perpendicular to the joint surface. There is no even bone surface but irregular bone trabeculae underlying the cartilage, suggesting that the animal was still actively growing. Indeed, endochondral bone trabeculae can be seen, replacing the cartilage. After removal of the cartilage layer, e.g., by soft tissue decay during fossilisation, a very uneven joint surface would result.

The margins of the cartilaginous joint surfaces about the thick joint capsule formed by ligamentous connective tissue. This is clearly visibly by its dark blue color in the Azan staining. The fibers of the joint capsule insert in the periosteal bone of either vertebral centrum.

2.7.2.3 Interpretation

The *Python* intervertebral joint is clearly of synovial type, with a fluid-filled intervertebral space. This space is surrounded by a joint capsule of connective tissue that contained the synovial fluid.

2.7.2.4 Discussion

Soft tissue histology based on decalcified stained microtome sections is able to image the joint morphology of intervertebral joint very well.

2.7.3 Ophidia indet. HLMD ME 7624b, middle Eocene, Messel, Germany (Figure S17)

2.7.1.1 Morphological observations

The specimen is a trunk segment of an indeterminate small snake, preserved as several segments consisting of strings of several articulated vertebrae. The intervertebral joints consist of the ball-and-socket joints typical for snakes. The bone is preserved on an artificial resin matrix and surrounded by dark organic remains.

2.7.1.2 Histological observations

The specimen is the least crushed of all the Messel fossils in this study. The bone of the vertebrae consists of compact, nearly avascular lamellar and parallel-fibered bone and limited areas of cancellous bone, located near the ends of the vertebral centra and in the neural spine. The vertebral centra are opisthocoelous, forming tightly articulating concave/convex joint surfaces. Much like in the extant snake described above, the joint surface is formed by files of hyaline cartilage cells oriented perpendicular to the curved (convex/concave) joint surfaces. At first, the files appear to be continuous across the joint cleft but upon closer inspection, the joint surface can be seen between the two cartilage layer. A similar phenomenon can be seen in the histological section of the extant snake where the joint surfaces appear to touch in places as a preparation artifact.

2.7.1.3 Interpretation and discussion

The Messel snake agrees in most respects with the extant snake in this study, but because of its smaller size, there are fewer files of cartilage cells. It is clear that the fossil snake possessed the same type of synovial joint consisting of hyaline cartilage and a thin synovial cleft as extant snakes. The complete preservation of the hyaline cartilage suggests that the cells were either already somewhat mineralised in life or that this happened during diagenesis.

*2.8. Sauropterygia*

2.8.1. *Placodus gigas* IGPB R 86, Muschelkalk, Middle Triassic, Germany (Figure S18)

2.8.1.1 Morphological observations

The vertebrae of *Placodus* and other placodonts are reported to be of the amphicoelous, non-notochordal type (*1*). The centra have articular surfaces with concave, funnel-shaped center and a weakly concave outer rim.

2.8.1.2 Histological observations

The sample is a transverse thin section through the center of the vertebral centrum. The section confirms that the notochordal foramen is essentially closed, possibly retaining a lumen of ca. 50 µm. In the inner region of the thin section, the endochondral bone trabeculae show extensive unmineralised and mineralised cartilage. In a polished section (not figured), it can be seen that endochondral bone formation was limited (resulting in the amphicoelous shape of the centrum) and, as in *Diadectes* and *Dimetrodon*, the outer concave rim is formed by endochondral bone formation. The concave area shows an irregular surface layer of chondrocyte lacunae and the convex area files of chondrocytes with intervening fibrous bone spicules.

2.8.1.3 Interpretation

The distribution of the cartilaginous tissues and bone with fibers, both endochondral and periosteal, is essentially the same to that in hupehsuchians and ichthyosaurs, suggesting the presence of an IVD. The extensive preservation of cartilage in the core of the trabeculae is a paedomorphic feature attributable to aquatic adaptation.

2.8.1.4 Discussion

The inference that placodonts such as *Placodus* had an IVD is supported by the observation that a highly paedomorphic form (especially in the vertebrae), *Pararcus diepenbroecki* (*78*), lacks notochordal centra despite lacking fusion between the left and right half of the neural arches, the retention of an embryonic state. This suggest that early in placodont ontogeny, the notochord already became discontinuous.

2.8.2. *Neusticosaurus peyeri* PIMUZ T 3768, Meride Limestone, Middle Triassic, Switzerland (Figure S19)

2.8.2.1 Morphological observations

The vertebrae of this small pachypleurosaur are pachyostotic, giving the centra a barrel-shape and the neural arches a swollen appearance (*79*). The dorsal vertebral column is little flexed and appears to have been fairly rigid in life. This is in accordance with distance between adjacent centra being less than 100 µm. Morphological observations of disarticulated *Neusticosaurus* specimens reveals that the articular surface of the centra is slightly sunken in the center and best called platycoelous with a shallow depression. The specimen is a small adult, and its humerus cross section was figured in Sander (*79*, fig. 25k).

2.8.2.2 Histological observations

The ventral side of the specimen is still embedded in the finely laminated sediment. The sediment did not enter the neural canal nor the intervertebral spaces. The neural canal is filled by coarse sparry calcite, with single crystals extending across its lumen. One of the intervertebral spaces is also filled by the same material, but the others contain a grey granular material that interfingers with sparry calcite. The shape of the intervertebral spaces in sagittal section is distinctly lens-shaped.

The articular surfaces of the centra clearly show altered cartilage layers. The superficial layer consists of irregularly arranged globular bodies and is about five globular body diameters thick. This layer is darker beige and less translucent than the underlying layer of serial cartilage, with the globular bodies arranged in files normal to the surface in the center of the articular surface. Towards the margin of the articular surface, the files diverge towards the perimeter and are more highly organised. There are few fibers in between the files in the marginal area and none in the center. The globular space in these files are commonly filled by dark, nearly opaque matter. Deeper into the bone, replacement of the globular bodies by bone tissue and the development of vascular canals is observed. The periosteal bone of the centra shows only a few isolated SF near the articular regions and thus little indication of intercentral connective tissue, neither dorsally nor ventrally.

2.8.2.3 Interpretation and discussion

The layer of darker beige globular bodies clearly represents a layer of actively proliferating, hyaline cartilage, and the globular bodies arranged in files are serial cartilage. The grey granular matter in the intervertebral space is not sufficiently organised to allow inferences as to its original nature. The tight fit of the perimeter of the articular surface combined with the lens-shaped intervertebral space is not consistent with a synovial joint. The proliferating hyaline cartilage is surprising thick for such a small animal. There is no evidence for remains of the notochord. While the cartilage layers are well preserved in the *Neusticosaurus* specimen, the tissue filling up the intervertebral space are not. The pattern of divergence and organisation of the files of serial cartilage suggests the presence of an IVD with a thin AF in *Neusticosaurus*.

2.8.3. *Plesiosaurus dolichodeirus* IGPB R 88, Lower Jurassic, Lyme Regis, England (Figure S20, S21)

2.8.3.1 Morphological observations

The two articulated vertebrae we studied are anterior dorsals, still retaining large intersegmental artery foramina (*80*). Like the much smaller *Neusticosaurus*, the *Plesiosaurus* specimen we studied has only a short distance of <1 mm between the articulating vertebrae in the marginal region, and the two centra are nearly touching at their perimeter. This is also seen in other plesiosaur specimens that are preserved in articulation in the matrix, e.g., the oldest plesiosaur *Rhaeticosaurus* (*81*) and also in the stem pistosauroid *Augustasaurus* (*71*, *82*). The articular surfaces of plesiosaur centra are essentially flat or slightly bowl-shaped, and generally described as platycoelous (*83*). The diameter of the articular surface exceeds that of the main part of centrum, leading to a flared appearance of the former.

2.8.3.2 Histological observations

The sagittal section reveals that the interior of the centrum is largely made up by relatively evenly but widely spaced and longitudinally arranged trabeculae of secondary origin. The primary periosteal cortex is extremely thin, no thicker than the average trabeculae. The articular faces are underlain by an even layer of trabecular bone of endochondral origin that lacks any indication of the retention of notochordal components and of a “notochordal pit”. The flared appearance of the articular surface was caused by the faster growth of the endochondral bone compared to the periosteal bone. The endochondral trabeculae are heavily remodeled and almost completely lack interstitial hypertrophied cartilage (unlike in *Placodus*). The central region of the bony articular face is overlain by a thin layer of poorly organised globular bodies about six to ten bodies thick. In the peripheral region, the globular bodies are arranged in clearly defined files with bony spicules showing fibers in between. Strong and distinctive Sharpey’s fibers are seen in the one fifth of the periosteal bone adjacent to the articular surface.

The intervertebral space contains a fill of variable appearance, with stringers of lighter and darker colors. The neural canal, on the other hand, is filled by carbonate matrix of even appearance. In one region, the material clearly shows a fibrous structure.

2..8.3.3 Interpretation

The layer of globular bodies represents a layer of hyaline cartilage of the proliferation zone. The uneven surface of this layer is in stark contrast with the smooth layer seen in the mosasaur. The roughness of the hyaline cartilage layer in the peripheral zone is consistent with fibrocartilage forming an AF. The strong Sharpey’s fibers in the periosteal bone close to the articular face suggest the presence of strong intervertebral ligaments. The inhomogeneous matrix in the intervertebral space may represent degraded fibrocartilage of the AF and the remains of the NP. In particular the fibrous material is suggestive of fibrocartilage and does not appear to be of microbial or invertebrate origin.

2.8.3.4 Discussion

In addition to the histological correlates suggesting the presence of an NP, this appears highly likely because of functional requirements. A connection only consisting of fibrocartilage would have restricted intervertebral movement too much.

*2.9. Thalattosauria*

2.9.1. *Xinpusaurus suni* HFUT GL 17003, 17006, Xiaowa Formation, early Late Triassic, Guanling, Guizhou, China (Figure S22)

2.9.1.1 Morphological observations

Thalattosaur vertebrae are variously described as amphicoelous or platycoelous in the literature (*84*, *85*). Vertebral morphology cannot be directly observed in our specimen because of matrix cover, but it can be inferred from the histological sections.

2.9.1.2 Histological observations

In general, bone tissue preservation is poor in both samples. The bone matrix appears opaque and has lost all birefringence in polarised light. In addition, the trabecular fabric is crushed in many places. Nevertheless, the histological sections of the two different-sized *Xinpusaurus* individuals reveal platycoelous vertebral centra with a very shallow depression resulting in a flattened lens-shaped intervertebral space, that is only one tenth the diameter of the articular faces. This can be estimated in the larger individual, HFUT GL 17006. The articular area is expanded relative to the body of the centrum, and this expansion is more pronounced than in *Plesiosaurus*. The encasing laminated sediment of the Xiaowa Formation does not seem to have entered the intervertebral space.

Both, the periosteal and the endochondral domain consist of cancellous bone. The trabeculae of the endochondral domain are longitudinally oriented. The entire articular face is covered by a layer of dark brown material in which there are files of globular bodies that are eight to ten layers thick. The files of globular bodies are perpendicular to the articular surface in its center but diverge increasingly towards the perimeter until they are at an angle of 45º to centrum long axis. The content of the globular bodies is translucent. This layer shows an abrupt border towards the center of the intervertebral space with another region of globular bodies. These bodies are much more irregularly arranged and lack the intervening dark-brown matrix. They are more irregular in size and commonly larger than the bodies in the brown matrix. This material appears to largely fill the intervertebral space, except for some globular bodies six to eight times as large as the previous ones. These bodies are translucent and have an indistinct border. They mostly have a circular cross section but some are more oval. Because of the poor preservation, SF cannot be detected in the periosteal bone.

2.9.1.3 Interpretation

The layer with the globular bodies in the brown matrix can confidently be interpreted as serial cartilage of the hypertrophy zone. At least the material adjacent to this layer must represent the proliferation zone. However, since the irregularly arranged bodies fill up the entire intervertebral space, some of it must also represent fibrocartilage or even notochordal cells. The diverging orientation of the files, especially well seen in HFUT GL 17006, in the perimeter is the same as observed in the mammalian AF, suggesting the presence of such a structure. The identity of the large globular bodies is enigmatic. They simply could represent gas bubbles that formed during soft tissue decay and later were filled in by diagenetic minerals. Alternatively, the large globular bodies might represent fecal pellets but this appears unlikely for the reasons discussed in conjunction with the *Stenopterygius* specimen. The intervertebral connection thus appears to have consisted of fibrocartilage in an AF and possible notochordal material, fitting the definition of an IVD.

2.9.1.4 Discussion

The *Xinpusaurus* material offers interesting soft-part preservation despite the poor bone tissue preservation. Taken at face value, *Xinpusaurus* and, by extension, other thalattosaurs possessed an IVDs. They clearly did not possess a synovial joint because the entire intervertebral space is filled by altered cartilaginous material and the layer of hyaline cartilage is very indistinct, especially in comparison with the mosasaur fossil.

*2.10. Crocodylia*

2.10.1. *Steneosaurus bollensis* IGPB R 663, Posidonienschiefer Formation, Lower Jurassic, Dotternhausen, Germany (Figure S23, S24)

2.10.1.1 Morphological observations

We studied an articulated pair of anterior dorsal vertebrae that are from an originally complete but somewhat disarticulated skeleton of the thalattosuchian crocodile *Stenosaurus bollensis* from the Early Jurassic Posidonienschiefer Formation of Dotternhausen, Germany. Thalattosuchian crocodiles are the second most common marine reptiles in this formation after ichthyosaurs. Unlike modern crocodiles, thalattosuchians have platycoelous vertebral centra with only slightly concave articular surfaces. Eusuchian crocodiles, on the other hand, have synovial ball-and-socket joints and procoelous vertebrae.

2.10.1.2 Histological observations

The preservation of the vertebrae suffers from some compaction, and the perimeter of the adjacent articular surfaces show some crushing, apparently shortening the distance between the two centra. The interior of the centra consist of loose trabeculae of secondary bone. Directly underlying the articular surfaces is denser bone tissue, forming a bony endplate about 3 mm in thickness. The center of the articular surface is covered by a layer of poorly organised, translucent globular bodies. The layer is about ten diameters of the bodies thick and in some areas is continuous with the bone tissue. Towards the perimeter, the globular bodies become arranged in files that are inclined outwards from the center of the surface. In-between the files, there are sometime bone spicules containing fibers of the same direction as the files. The intervertebral space is filled by coarse crystal aggregates that seem to have replaced dark organic matter during their growth. The same type of material is also present in the neural canal and adjacent to the vertebrae.

2.10.1.3 Interpretation

The globular bodies are the remains cartilage cells that in the living animal were gradually mineralising away from the articular surface. The inclined files and bone spicules are the attachment area of the fibrocartilage of the AF. The poorly organised cartilage cells in the central region of the articular surface must have underlain an NP that occupied the intervertebral space.

2.10.1.4 Discussion

Unlike extant, eusuchian crocodiles, thalattosuchians with their platycoelous centra apparently had an IVD and not a synovial joint. This is strongly suggested by the distribution of the types of preserved articular cartilage in the specimen studied here.

*2.11. Dinosauria*

2.11.1. Hadrosauridae indet. UALVP 59650, Dinosaur Park Formation, Late Cretaceous, Alberta, Canada (Figure S25)

2.11.1.1 Morphological observations

In general, hadrosaur dorsal vertebral centra have a simple, cylindrical morphology with platycoelous articular surfaces. The specimen we studied is an isolated hadrosaur dorsal vertebra, and because of constraints on section size, the sagittal histological section only covers the outer part of an anterior or posterior articular surface.

2.11.1.2 Histological observations

The interior of the centrum shows loose, longitudinally arranged trabeculae of secondary bone that become denser and more isotropic below the articular surface. The articular surfaces itself is covered by seemingly hollow globular structures set in files but with thick layers (wider than the diameter of the globules) of intervening fibrous material that appears to be mineralised in cross-polarised light. The fibers are an order of magnitude smaller than the globules and densely packed. They appear to be oriented mainly perpendicular to the plane of section, i.e., circumferentially oriented except close to the files, where they are oriented in the direction of the files. In the area covered by the section, all files are inclined towards the perimeter of the articular surface, but inclination increases from <45º to >60º. The globule-and-fiber layer is also preserved in some interstitial spaces of the secondary trabeculae.

2.11.1.3 Interpretation

The arrangement of globular bodies and fibers is unique among the material studied. We interpret this tissue as the mineralised insertion of fibrocartilage into the bony endplate. The increasing inclination of the chondrocyte files towards the margin is consistent with an AF. The observation that secondary bone lamellae replace the globule-and-fiber layer is consistent with its interpretation as mineralised fibrocartilage because secondary replacement explains the lack of a proliferation zone and hyaline cartilage observed in the other samples.

2.11.1.4 Discussion

Since the section does not cover the center of the articular surface, it does not inform on the presence of a NP. However, the orientation of the fibers in the AF is suggestive of it enclosing an NP because otherwise the increasing inclination would be difficult to explain. If there were only fibrocartilage or a synovial joint between the vertebrae in hadrosaurs, the files would be oriented straight across the intervertebral space, as in our squamate samples. The mineralisation and replacement of fibrocartilage by bone may be explained by the large size or an advanced ontogenetic stage of the individual, where chondrogenesis had long ceased

2.11.2. Ornithomimidae indet. UALVP 56951, Dinosaur Park Formation, Late Cretaceous, Alberta, Canada (Figure S26)

2.11.2.1 Morphological observations

Ornithomimosaur dorsal vertebrae are of the platycoelous type with weakly concave articular surfaces (*36*). Our sample only covers part of either the anterior or posterior articular surface, but unfortunately not the peripheral region, nor does it cover the interior of the centrum. The articular surface is still partially covered by sediment.

2.11.2.2 Histological observations

The bony endplate consists of a fairly loose arrangement of bony trabeculae of secondary origin. The articular surface is underlain by a thin layer of bone which is part of this trabecular system. Covering the bone, there is a thin layer of globular structures set in a mineralised matrix. The globular structures are arranged in poorly defined files that are at most eight globules thick and are oriented perpendicular to the surface.

2.11.2.3 Interpretation and discussion

The globular layer in the mineralised matrix represents hypertrophied cartilage, with the proliferation zone not being preserved. Endochondral bone formation had ceased, and a layer of hyaline cartilage must have covered the hypertrophied cartilage in life. The poor definition of the files and their lack of inclination suggest that this was not a region of insertion of fibrocartilage but that a NP was located in the intervertebral space. While the sample only offers incomplete coverage, it is consistent with the interpretation that ornithomimosaurs possessed an IVD.

2.11.3. Dromaeosauridae indet. UALVP 56952, Dinosaur Park Formation, Late Cretaceous, Alberta, Canada (Figure S27)

2.11.3.1 Morphological observations

Dromaeosaur dorsal vertebrae are of the platycoelous type and may have pleurocoels (*36*).

2.11.3.2 Histological observations

The dromaeosaurid dorsal vertebral centrum we sectioned sagittally is nearly twice as tall dorsoventrally than long and has flat articular surfaces. The interior of the centrum has a large empty space that was pneumatised as indicated by its surrounding trabeculae consisting of pneumosteum. Pneumosteum is a peculiar tissue recently described (*86*) in secondary bone trabeculae that formed around pneumatised cavities in the postcranial skeleton of saurischian dinosaurs, including birds. The trabecular architecture of the centrum appears to be controlled by its pneumatisation, with the pneumatised space being surrounded by a clear lining of pneumosteum followed by a loose trabecular network. However, the microanatomical architecture of this trabecular network differs in the two articular surfaces, with the direction of anterior being unknown. Towards one articular surface, the trabeculae condense into a poorly defined bony endplate of discontinuous trabeculae. Towards the other, a continuous layer of bone underlies a similar, poorly defined bony endplate. Only very few and isolated globular bodies are seen in the outermost bone matrix.

2.11.3.3 Interpretation

Endochondral ossification had ceased on the specimen and only a thin and apparently discontinuous layer of hypertrophied cartilage covers the articular surface. The nature of the hyaline cartilage is difficult to infer because it is not preserved. However, the thinness of the preserved hypertrophied cartilage would be consistent with the assumption that the layer of hyaline cartilage was very thin.

2.11.3.4 Discussion

There is no indication of an AF in the dromaeosaur nor of an NP. This dinosaur probably did not have an IVD but already a bird-type intervertebral joint. The meaning of the anteroposterior asymmetry of the trabecular architecture is not clear at present.

2.11.4. Aves indet. HLMD-ME 1022, middle Eocene, Messel, Germany (Figure S28)

2.11.4.1 Morphological observations

The specimen we sampled is a partial skeleton of a medium-sized bird preserving the trunk region. The bones appear crushed and are covered by a dark organic film. The sample covers four dorsal vertebrae and three intervertebral joints.

2.11.4.2 Histological observations

The histologic section reveals that the specimen is heavily crushed, involving both brittle and seemingly also plastic deformation, as seen in the other Messel specimens. The articular surfaces of the centra are affected by this crushing, and their exact morphology is difficult to ascertain, except that there seems to have been a tight fit of curved surfaces. The area between adjacent vertebrae is filled by dark brown amorphous material. The articular surfaces appear to be underlain by dense bone and show only a few globular bodies set in a mineralised matrix. These are scattered near the surface.

2.11.4.3 Interpretation

Despite the heavy crushing, it is clear that the Messel bird did not have much cartilage in its intervertebral joints. The scattered globular bodies represent a thin layer of hypertrophied cartilage. The joint thus appears to have been the same as in other bird dorsal vertebrae.

2.11.4.4 Discussion

Despite the great size difference, there is a distinct similarity between the articular surface histology of the dromaeosaur and the bird. Dromaeosaur dorsals thus appear to have already possessed a bird-type joint.

References

48. Arratia, G., Schultze, H. P., Casciotta, J. Vertebral column and associated elements in dipnoans and comparison with other fishes: development and homology. *Journal of Morphology* 250(2), 101-172 (2001).

49. Goldman, K.J., Cailliet, G.M., Andrews, A.H., Nathanson, L.J. Assessing the Age and Growth of Chondrichthyan Fishes. In *Biology of Sharks and their Relatives* (ed J. Carrier, J. A. Musick, M. R. Heithaus) 423-451 (Boca Raton: CRC Press, 2012).

50. Ridewood, W.G., MacBride, E.W. On the calcification of the vertebral centra in sharks and rays. *Philosophical Transactions of the Royal Society B* 210, 311-407 (1921).

51. Laerm, J. On the origin of rhipidistian vertebrae*. Journal of Paleontology* 53(1), 175-186 (1979).

52. Sallan, L.C. Tetrapod-like axial regionalization in an early ray-finned fish. *Proceedings of the Royal Society B* 279, 3264-3271 (2012).

53. Bartsch, P. Funktionelle Morphologie und Evolution des Axialskelettes und der Caudalis ursprünglicher Knochenfische. *Palaeontographica Abt. A* 204, 117-236 (1988).

54. López-Arbarello A, Sferco E. Neopterygian phylogeny: the merger assay. *Royal Society Open Science*, 5 (3): 172337 (2018).

55. Arratia, G. Morphology, taxonomy, and phylogeny of Triassic pholidophorid fishes (Actinopterygii, Teleostei). *Journal of Vertebrate Paleontology*, 33 (sup1), 1-138 (2013).

56. Laerm, J. The development, function, and design of amphicoelous vertebrae in teleost fishes. *Zoological Journal of the Linnean Society*, 58: 237-254 (1976).

57. Christ, B., Huang, R., Scaal, M. Amniote somite derivatives. *Developmental Dynamics*, 236(9), 2382-2396 (2007).

58. Huang, R., Zhi, Q., Wilting, J., Christ, B. The fate of somitocoele cells in avian embryos. *Anatomy and Embryology (Berlin)* 190, 243-250 (1994).

59. Mittapalli, V. R., Huang, R., Patel, K., Christ, B., Scaal, M. Arthrotome: a specific joint forming compartment in the avian somite. *Developmental Dynamics*, 234(1), 48-53 (2005).

60. Stemple, D. L. Structure and function of the notochord: an essential organ for chordate development. *Development*, 132(11), 2503-2512 (2005).

61. Hautier, L.; Weisbecker, V.; Sánchez-Villagra, M.R.; Goswami, A. Asher, R.J. Skeletal development in sloths and the evolution of mammalian vertebral patterning. *Proceedings of the National Academy of Sciences*, 107(44): 18903-18908 (2010).

62. Hautier, L.; Stansfield, F.J.; Allen, W.R.T. Asher, R.J. Skeletal development in the African elephant and ossification timing in placental mammals. *Proceedings of the Royal Society B: Biological Sciences*, 279 (1736): 2188-2195 (2013).

63. Boszczyk, B.M.; Boszczyk, A.A., Putz, R. Comparative and functional anatomy of the mammalian lumbar spine. *The Anatomical Record* 264 (2): 157-168 (2001).

64. Strong, R.M. The order, time, and rate of ossification of the albino rat (*Mus norvegicus albinus*) skeleton. *American Journal of Anatomy*, 36(2): 313-355 (1925).

65. Dawson, A.B. Further studies on the epiphyses of the albino rat skeleton, with special reference to the vertebral column, ribs, sternum, and girdles. *The Anatomical Record,* 34(5): 351-363 (1927).

66. Danowitz, M., Solounias, N. The cervical osteology of *Okapia johnstoni* and *Giraffa camelopardalis*. *PlosOne*, 10(8): e0136552 (2015).

67. Kemp, T.S. The origin and early radiation of therapsid mammal-like reptiles: a palaeobiological hypothesis. *Journal of Evolutionary Biology,* 19(4): 1231-1247 (2006).

68. Dar, G.; Masharawi, Y.; Peleg, S.; Steinberg, N.; May, H.; Medlej, B.; Peled, N., Hershkovitz, I. The epiphyseal ring: a long forgotten anatomical structure with significant physiological function. *Spine*, 36(11): 850-856 (2011).

69. Romer, A. S., Price, L. W. Review of the Pelycosauria. *Special Paper of the Geological Society of America* 28, 1-538 (1940).

70. Moon, B. C. A new phylogeny of ichthyosaurs (Reptilia: Diapsida). *Journal of Systematic Palaeontology* 17, 1-27 (2019).

71. Sander, P. M., Rieppel, O. C., Bucher, H. A new pistosaurid (Reptilia: Sauropterygia) from the Middle Triassic of Nevada and its implications for the origin of plesiosaurs. *Journal of Vertebrate Paleontology* 17, 526-533 (1997).

72. Fröbisch, N. B., Fröbisch, J., Sander, P. M., Schmitz, L., Rieppel, O. Macropredatory ichthyosaur from the Middle Triassic and the origin of modern trophic networks. *Proceedings of the National Academy of Sciences, USA* 110 1393–1397 (2013).

73. Fröbisch, N. B., Sander, P. M., Rieppel, O. A new species of *Cymbospondylus* (Diapsida, Ichthyosauria) from the Middle Triassic of Nevada and a re-evaluation of the skull osteology of the genus. *Zoological Journal of the Linnean Society of London* 147, 515-538 (2006).

74. Schmid-Röhl, A., Röhl, J., Oschmann, W., Frimmel, A. Der Posidonienschiefer (Lias-epsilon) Südwestdeutschlands: hochauflösende geochemische, palökologische und sedimmetologische Untersuchungen. *Zentralblatt für Geologie und Paläontologie, Teil I (1997)*, (7-9), 989-1004 (1999).

75. Wettstein O v. 1. Ordnung der Klasse Reptilia: Rhynchocephalia. In *Handbuch der Zoologie Sauropsida: Allgemeines. Reptilia. Aves* (eds. W. Kükenthal, T. Krumbach, 1–235 (Berlin:de Gruyter 1931).

76. Schauinsland, H. Die Entwicklung der Eihäute der Reptilien und der Vögel. *Handbuch der Vergleichenden und Experimentellen Entwinklungslehre der Wirbelthiere*, 1 (1902).

77. G. A. Goldfuß, Der Schädelbau des *Mosasaurus*, durch Beschreibung einer neuen Art dieser Gattung erläutert. *Nova Acta Academa Ceasarea Leopoldino-Carolinae Germanicae Natura Curiosorum* 21, 1-28 (1845).

78. Klein, N., Scheyer, T. M. A new placodont sauropterygian from the Middle Triassic of the Netherlands. *Acta Palaeontologica Polonica* 59, 887-890 (2014).

79. Sander, P. M. The pachypleurosaurids (Reptilia: Nothosauria) from the Middle Triassic of Monte San Giorgio (Switzerland) with the description of a new species. *Philosophical Transactions of the Royal Society of London* B 325, 561-670 (1989).

80. Wintrich, T., Scaal, M., Sander, P. M. Foramina in plesiosaur cervical centra indicate a specialized vascular system. *Fossil Record* 20, 279-290 (2017).

81. Wintrich, T., Hayashi, S., Houssaye, A., Nakajima, Y., Sander, P. M. A Triassic plesiosaurian skeleton and bone histology inform on evolution of a unique body plan. *Science Advances* 3, e1701144, 1701141-1701111 (2017).

82. Rieppel, O., Sander, P. M., Storrs, G. W. The skull of the pistosaur *Augustasaurus* from the Middle Triassic of northwestern Nevada. *Journal of Vertebrate Paleontology*, 22(3), 577-592 (2002).

83. Benson, R. B. J., Druckenmiller, P. S. Faunal turnover of marine tetrapods during the Jurassic–Cretaceous transition. *Biological Reviews* 89, 1-23 (2014).

84. Liu, J., Rieppel, O. Restudy of *Anshunsaurus huangguoshuensis* (Reptilia: Thalattosauria) from the Middle Triassic of Guizhou, China. *American Museum Novitates* 3488, 1-34 (2005).

85. Rieppel, O., Liu, J. On *Xinpusaurus* (Reptilia: Thalattosauria). *Journal of Vertebrate Paleontology* 26, 200-204 (2006).

86. Lambertz, M., Bertozzo, F., Sander, P. M. Bone histological correlates for air sacs and their implications for understanding the origin of the dinosaurian respiratory system. *Biology Letters* 14, 1-5 (2018).

Supporting Table 1: Phylogenetically organised list of fossil and extant specimens sampled for this study. The higher clade they belong to, the specimen numbers, the region of the spine represented, the kind of section and the type of joint and character state. Abbreviations for kind of section: GS, ground petrographic section; MS, decalcified and stained microtome section. Abbreviations for kind of specimen: IA, Intervertebral articulation; IV, isolated vertebra. Abbreviations for type of joint: AC, amphicoelous; CN, continuous notochord; PC, platycoelous; IVD, intervertebral disc; SBSJ, synovial joint, ball-and-socket joint; SJS, saddle-shaped; NN, no notochord. Bold indicates observed soft parts. Explanation of character states: 0: CN, AC; 1: IVD, AC; 2: IVD, PC; 3: SBSJ; 4: SJS, AGP. Collections acronyms: HFUT, Hefei University of Technology, Hefei, China; HLMD-ME, Hessisches Landesmuseum Darmstadt, Messel collection, Darmstadt, Germany; NHMW, Naturhistorisches Museum Wien, Vienna, Austria; PIMUZ, Paläontologisches Institut und Museum Universität Zürich, Zurich, Switzerland; PXD, private collection Xaver Donhauser, Stuttgart, Germany; IGPB, Institute of Geoscience, Paleontology Collection, University of Bonn, Bonn, Germany; UALVP, University of Alberta Collection of Vertebrate Paleontology.

| Taxon | Higher Clade | Specimen reference | Thin section reference | Kind of section | Region | Joint type, state |
| --- | --- | --- | --- | --- | --- | --- |
| *Diadectes sideropelicus* | Diadecto-morpha | IGPB A 169 | IGPB A 169 | GS, IV | Dorsal vertebra | 0: CN, AC |
| *Dimetrodon* *natalis* juvenile | Synapsida | IGPB R 653a | IGPB R 653a | GS, IV | Dorsal vertebra | 0: CN, AC |
| *Dimetrodon* *natalis* adult | Synapsida | IGPB R 652a | IGPB R 652b | GS, IV | Dorsal vertebra | 0: CN, AC |
| *Dimetrodon natalis* | Synapsida | IGPB R 658 | IGPB R 658 | GS, IA | Sacral vertebrae | 0: CN, AC |
| non-mammalian Therapsida | Synapsida | Romer 1956, Kemp 2006 |  |  | Dorsal vertebra | 1/2: IVD, AC/PC |
| basal mammals | Synapsida | Kemp 2006 |  |  | Dorsal vertebra | 1/2: IVD, AC/PC |
| *Phoca vitulina* | Mammalia, Carnivora | IGPB M 60 | IGPB M 60 | GS, IV | Anterior dorsal vertebra | 2: IVD, PC |
| *Eurohippus* sp. | Mammalia, Perissodactyla | HLMD-ME 106 | HLMD-ME 106 | GS, IA | Lumbar vertebra | 2: IVD, PC |
| *Eurohippus* sp. | Mammalia, Perissodactyla | HLMD-ME 139 | HLMD-ME 139 | GS, IA | Lumbar vertebra | 2: IVD, PC |
| *Eurohippus* sp. | Mammalia, Perissodactyla | HLMD-ME 7835 | HLMD-ME 7835 | GS, IA | Lumbar vertebra | 2: IVD, PC |
| *Stereosternum tumidum* | Mesosauria | IGPB R 622 | IGPB R 622 | GS, IA | Dorsal vertebrae | 0: **CN**, AC |
| *Captorhinus* sp. | Capthorhinidia | LeBlanc et al. 2018 | Fig. 2E, Extended Data Figure 1g | GS, IV | Caudal vertebrae | 0: **CN**, AC |
| *Nanchango-saurus suni* | Hupehsuchia | HFUT YAN-10-02 | HFUT Uk 1703 | GS, IA | Middle dorsal vertebrae | 1/2: IVD, AC/PC |
| Cymbospondylidae new taxon A | Ichthyosauria | LACM DI 158109 | LACM DI 158109 | GS, IA | Anterior dorsal vertebrae | 1: IVD, AC |
| cf. *Cymbospondylus* | Ichthyosauria | IGPB R 660 | IGPB R 661 | GS, IA | Posterior dorsal vertebrae | 1: IVD, AC |
| *Stenopterygius* sp. | Ichthyosauria | IGPB R 661 | IGPB R 661 | GS, IA | Anterior dorsal vertebrae | 1: IVD, AC |
| *Leptopterygius* sp. | Ichthyosauria | IGPB R 235 | IGPB R 235 | GS, IA | Anterior caudal vertebrae | 1: IVD, AC |
| *Sphenodon punctatus* | Sphenodontia | NHMW 8108:2 | NHMW 8108:2 | MS, IA | Dorsal and sacral vertebra | 0: **CN**, AC |
| *Mosasaurus missouriensis* | Mosasauroidea | IGPB Goldfuß 1230 (Type specimen of *M. maximiliani*) | IGPB Goldfuß 1230 | GS, IA | Posterior dorsal and anterior caudal vertebrae | 3: SBSJ |
| *Python* sp. | Ophidia | IGPB R 662 | IGPB R 662 | MS, IA | Dorsal vertebra | 3: SBSJ |
| Ophidia indet. | Ophidia | HLMD ME 7624b | HLMD ME 7624b | GS, IA | Dorsal vertebra | 3: SBSJ |
| *Placodus gigas* | Placodontia | IGPB R 86 | IGPB R 86 | GS, IV | Dorsal vertebra | 1: IVD, AC |
| *Neusticosaurus peyeri* | Sauropterygia | PIMUZ T 3768 | PIMUZ T 3768 | GS, IA | Dorsal vertebra | 2: IVD, PC |
| *Plesiosaurus dolichodeirus* | Sauropterygia | IGPB R 88 | IGPB R 88 | GS, IA | Anterior dorsal vertebrae | 2: IVD, PC |
| *Xinpusaurus suni* | Thalattosauria | HFUT GL 17003 | HFUT GL 17003 | GS, IA | Anterior dorsal vertebrae | 2: IVD, PC |
| *Xinpusaurus suni* | Thalattosauria | HFUT GL 17006 | HFUT GL 17006 | GS, IA | Posterior dorsal vertebrae | 2: IVD, PC |
| *Steneosaurus bollensis* | Thalattosuchia | PXD | IGPB R 663 | GS, IA | Anterior dorsal vertebrae | 2: IVD, PC |
| *Alligator mississippiensis* | Eusuchia | Wettstein, 1962 |  |  | Dorsal vertebra | 3: SBSJ |
| Hadorsauridae indet. | Dinosauria | UALVP 59650 | UALVP 59650 | GS, IV | Dorsal vertebra | 2: IVD, PC |
| Ornithomimidae indet. | Dinosauria | UALVP 59651 | UALVP 59651 | GS, IV | Dorsal vertebra | 2: IVD, PC |
| Dromaeosaur-idae indet. | Dinosauria | UALVP 56952 | UALVP 56952 | GS, IV | Dorsal vertebra | 4: IVD, PC |
| cf. *Messelornis* | Aves | HLMD-ME 13019 | HLMD-ME 13019 | GS, IA | Dorsal vertebra | 4: SJS |
